# Deriving Organism-Relevant Nanoparticle Safety Exposure Limits from *In Vitro* Toxicity Data

**DOI:** 10.1021/acs.est.6c13254

**Published:** 2026-10-28

**Authors:** Jimeng Wu, Peter Wick, Bernd Nowack

**Affiliations:** † Empa, Swiss Federal Laboratories for Materials Science and Technology, 111825 Nanomaterials in Health Laboratory, Lerchenfeldstrasse 5, 9014 St. Gallen, Switzerland; ‡ Empa, Swiss Federal Laboratories for Materials Science and Technology, Technology and Society Laboratory, Lerchenfeldstrasse 5, 9014 St. Gallen, Switzerland

**Keywords:** nanotoxicology, human health risk assessment, new approach methodologies, *in vitro* *in vivo* extrapolation, Bayesian dose–response modeling, uncertainty propagation

## Abstract

Establishing human safety limits of engineered nanoparticles remains a challenge. *In vitro* assays have become a major toxicity data resource, driven by the demand for new approach methodologies (NAMs) that reduce animal testing. However, robust tools that bridge *in vitro* data with *in vivo* outcomes are still lacking. Here, we present an integrated computational framework using physiologically based pharmacokinetic (PBPK) modeling to derive organism-level BMD_10_-based dose estimates directly from *in vitro* liver toxicity data. For 4 nondissolvable nanoparticles (TiO_2_, SiO_2_, Au, and graphene oxide), we curated *in vitro* toxicity data sets spanning 7 liver-relevant cell systems (5 human and 2 mouse) across 24 assays and estimated *in vitro* effect thresholds using the benchmark dose for a 10% response (BMD_10_). Mouse *in vitro* BMD_10_ values were then reverse-translated into predicted BMD_10_^IVIVE^ values using a validated mouse PBPK model and benchmarked against available mouse-derived *in vivo* effect threshold (BMD_10_^eq^) data to ensure consistency. The resulting nanoparticle-specific BMD_10_^IVIVE^ ranges showed qualitative concordance with animal-derived reference doses, supporting the biological plausibility of these screening-level dose estimates. We further applied the same workflow to human liver cell systems to derive human-relevant BMD_10_-based points of departure. This approach establishes a scalable and prospective animal-free workflow for human nanoparticle risk assessments, overcoming a critical translational gap.

## Graphical abstract

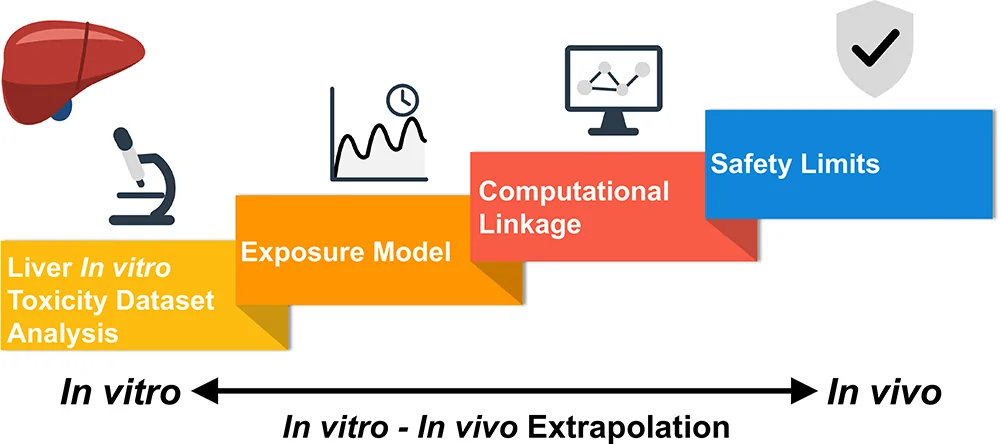

## 1 Introduction

Engineered nanoparticles are used in a large number of different industrial and consumer applications due to their specific functionality and versatility.^1^ Alongside their benefits, evidence also indicates potential human health and environmental risks across the life cycle from production and use to end-of-life.^2,3^ Establishing protective human exposure safety limits for these materials remains a persistent challenge. Conventional risk assessment relies on *in vivo* data that are costly, time-consuming, ethically constrained,^4^ and require interspecies extrapolation. To meet testing demands while following the “replacement, reduction, and refinement” 3R principles,^5^ new approach methodologies (NAMs) have expanded to complement and substitute animal experiments.^6,7^ However, human-based *in vitro* assays—though scalable and mechanistic—require toxicokinetic modeling to translate *in vitro* effective concentrations to human exposure metrics, including deterministic^8^ or probabilistic approaches.^9^ With the proposal of the Safe and Sustainable by Design (SSbD) framework,^10^ there is a pressing need for easy-to-apply and reproducible approaches that convert *in vitro* data into human-relevant safety limits with quantified uncertainties.

Physiologically based pharmacokinetic (PBPK) models^11^ mechanistically represent nanoparticle distribution, clearance, and organ retention across tissues, enabling prediction of organ exposures under diverse exposure scenarios, and have been developed for several nanoparticles and applied in risk-relevant contexts. Bachler et al.^12^ developed a human PBPK model for dietary risk assessment of TiO_2_ nanoparticles, converting reported daily dietary intake in the German population into organ-specific internal titanium levels. PBPK-based evaluations of silver nanoparticles have modeled biodistribution of both nanosilver and dissolved silver ions^13^ and illustrate application across multiple exposure scenarios (e.g., dietary/oral intake, dermal absorption, and occupational exposure).

PBPK-enabled *in vitro* to *in vivo* extrapolation (IVIVE) has been established in pharmaceutical development^14,15^ and chemical toxicology^16–18^ to link *in vitro* effective concentrations to *in vivo* exposure profiles. Lin et al.^19^ proposed a general Nano-IVIVE-PBPK framework for extrapolating cellular and tissue dosimetry from *in vitro* to *in vivo*. Several studies have implemented specific elements of this approach. Examples include a probabilistic human risk assessment for gold nanoparticles^20^ integrating *in vitro* cell viability data with a PBPK model to estimate human equivalent external doses for points of departure after intravenous injection. Laomettachit et al.^21^ developed a two-step modeling approach for TiO_2_ combining intravenous injection-based PBPK predicted liver concentrations with a cell response model, relating *in vitro* effect levels to corresponding tissue exposure to explain liver cell viability caused by TiO_2_ nanoparticle exposure. Computational tools have also been used to estimate TiO_2_ nanoparticle deposition in the respiratory tract.^22^ Other studies have advanced complementary aspects of the nano-IVIVE landscape. Chou et al.^23^ developed a multiroute Bayesian PBPK model for gold nanoparticles with quantitative structure–activity relationship (QSAR)-based parameter prediction, showing that traditional route-to-route extrapolation from small molecules does not apply to nanoparticles. Dubaj et al.^24^ parametrized a PBPK model for PEGylated gold nanoparticles validated against rat biodistribution. Beyond PBPK-based frameworks, Huang et al.^25^ applied a data-mining approach to predict *in vivo* chronic lung toxicity from cytotoxicity data across multiple metal oxide nanoparticles. These approaches remain largely nanoparticle- and scenario-specific and rarely provide an uncertainty-aware workflow applicable across heterogeneous *in vitro* data sets to derive safety limits for multiple nanoparticles.

Although PBPK models are generally developed for the whole body, occupational and inhalation IVIVE-PBPK models often focus on the lung,^26–28^ given its importance in nanoparticle uptake and occupational exposure.^29^ Other organs were also important. The liver is a particularly relevant target organ for toxicity assessment, given its central role in xenobiotic detoxification^30,31^ and its frequent involvement in nanoparticle uptake and retention following systemic exposure.^32^ Liver-derived cells (e.g., HepG2 and primary hepatocytes),^33^ macrophage models,^34^ and lung epithelial cells^35^ are frequently used in *in vitro* nanotoxicity testing.^36^ While three-dimensional (3D) spheroids might better capture tissue architecture, liver cell metabolisms, and diffusion gradients,^37–39^ incorporating them into IVIVE frameworks requires additional dosimetry characterization. Moreover, these more advanced models are still under development^40,41^ and not yet widely applied to nanotoxicity assessment. The available data remain insufficient for harmonized database construction based on advanced models and are therefore not included.

Here, we present an integrated, uncertainty-aware framework that translates *in vitro* nanotoxicity data to organism-level BMD_10_-based dose estimates by combining *in vitro* effective concentrations with PBPK modeling. Our aim is a standardized workflow applicable across heterogeneous literature-derived data sets, placing multiple nanoparticles on a common, comparable safety scale for early-stage hazard screening and prioritization under SSbD. We curate and quality-control dose–response data sets across human and mouse liver cell systems. Mouse data allow validating the IVIVE pipeline against available mouse *in vivo* benchmarks, whereas the human data represent the ultimate target of the workflow, deriving human-relevant BMD_10_-based points of departure, for which limited comparable *in vivo* benchmarks exist. We focus on the liver in this study and use *in vitro* two-dimensional (2D) cell line data, given data availability and the coherence between *in vitro* and *in vivo*. We assume equivalence between nominal *in vitro* concentrations and unbound *in vivo* liver tissue concentrations, a simplification whose plausibility is tested by benchmarking the IVIVE-translated mouse doses against independently derived *in vivo* reference values. Since the nanoparticle PBPK parametrization and *in vivo* benchmarks are most available for injection-based dosing, we develop the workflow for injection exposures as a tractable first step toward route-specific extensions. We standardize concentration–response modeling to derive BMD_10_ metrics, explicitly addressing flat or inconsistent curves and propagating parameter uncertainty. We define BMD_10_^IVIVE^ as the PBPK translated dose estimate derived from *in vitro* data sets and benchmark it against an *in vivo*-derived BMD_10_^eq^ (converted from reported mouse *in vivo* endpoints) for mice. Equivalent human BMD_10_^IVIVE^ values from *in vitro* human cell toxicity data were also calculated and reported here.

Together, the harmonized data set, the Bayesian dose–response estimation workflow, and the mechanistic IVIVE coupling operationalize a reproducible route from bench measurements to human-relevant screening-level points of departure for hazard assessment and prioritization, supporting SSbD objectives.

## 2 Methodology and Data

### 2.1 Data Collection

We compiled nanoparticle *in vitro* toxicity data sets from peer-reviewed literature (2010–2022) for both mice and humans, with an emphasis on liver-derived cells and primary cell lines. *In vitro* studies cover nondissolvable nanoparticles (TiO_2_, SiO_2_, Au, Fe_2_O_3_, graphene oxide (GO) (with reduced graphene oxide (rGO))) across multiple cell types and assays. Notably, all compiled TiO_2_ *in vitro* experiments were conducted under standard dark incubator conditions, therefore reflect intrinsic particle toxicity, and do not account for potential photocatalytic enhancement of reactive oxygen species (ROS) generation. *In vitro* studies were retained given the following criteria: (i) when they reported ≥3 valid dose–response points including the negative control, and (ii) showed a concentration-dependent and biologically plausible cytotoxic effect trend, to support reliable dose–response curve fitting. All collected data sets include a negative control, and the extracted dose–response data represent mean values from replicate measurements (*n* ≥ 3 per concentration), as reported in the original publications.

The endpoint inclusion was determined by data availability in the curated literature and by suitability for dose–response curve fitting. The retained assay modalities included cytotoxicity (including LDH), metabolic cell stress and organ function damage, cytokine readouts, and genotoxicity endpoints, which constitute the most frequently reported toxicity outcomes for the nanoparticles considered. We grouped them by biological output type to apply harmonized modeling strategies (Table 1). The compiled raw data sets are provided in Table S1 (mouse) and Table S2 (human).

**Table 1:** Classification of *In Vitro* Assays Used for Dose–Response Modeling, Grouped by Biological Function, with Associated Metrics and Endpoint Normalization Strategies

class	category	assay(s)	biological role	endpoint type	key interpretation metric
cytotoxicity	cell viability	MTT, Alamar Blue, EZ-Cytox, MTS, WST-1, Trypan Blue, NRU, ATP, CCK8	measure mitochondrial or metabolic activity of viable cells	% viability	BMD_10_ (effective concentration with min and max limit as 0 and 1)
	membrane integrity	LDH	detects loss of membrane integrity via enzyme leakage	% cytotoxicity	BMD_10_ (effective concentration with min and max limit as 0 and 1)
metabolic cell stress	oxidative stress	DCFDA, MDA, GSH, GGT	assess ROS production and antioxidant enzyme response	fold change or raw activity (e.g., U/mg)	BMD_10_ (effective concentration, if fitting possible)
	tissue injury	ALT, AST, ALP, TBIL, protein concentration	indicators of hepatocellular, cholestatic, or kidney damage	fold change or absolute activity (U/L)	BMD_10_ (effective concentration, if fitting possible)
genotoxicity	DNA damage	alkaline comet assay	detects single-strand DNA breaks and alkali-labile sites	% tail intensity, % tail DNA, tail moment, tail length	BMD_10_ (effective concentration, if fitting possible)
cytokine/inflammatory	inflammatory/cytokine	TNF-α, IL-6, IL-8, IL-1b	markers of proinflammatory signaling and immune activation	concentration or fold change	dose at which significant elevation occurs (2-fold change compared to negative value)

As for *in vivo* data for validating IVIVE results, we collected mouse animal studies reporting liver-related toxicity outcomes (e.g., AST, SOD, CD68, liver granuloma number and size, IL-6, and IL-8 expression) as well as mortality studies. Owing to data scarcity, we additionally recorded selected reproductive toxicity endpoints. We limited inclusions to injection-administered studies, consistent with the nanoparticle PBPK model^42^ used in this framework and the predominance of injection-based dosing in the curated animal studies.

Literature searches were performed in Web of Science with supplementary searches in Google Scholar, using combinations of terms: (“nanoparticle” or “nanomaterial”) and (“TiO_2_” or “SiO_2_” or “gold” or “graphene oxide”) and (“liver” or “hepa” or “liver toxicity”) and (“cell” or “in vitro” or “mouse”).

### 2.2 *In Vitro* Data Processing and Dose–Response Modeling

*In vitro* assays were categorized by biological output type and expected response profiles, allowing harmonized processing across mechanistically related endpoints, into four classes: cytotoxicity, metabolic cell stress responses, genotoxicity, and cytokine markers.

Cytotoxicity assays, including MTT, Alamar blue, Cell Titer-blue, CCK, and LDH assays, quantify cell viability or membrane integrity and typically exhibit a monotonic decrease (opposite for LDH) in signal with increasing dose. These assays possess a biologically interpretable response range—from 100% viability (untreated control) to 0% (complete cytotoxicity)—facilitating sigmoidal dose–response modeling using the three-parameter Hill equation with a fixed upper asymptote.

1 $$y=a+\left(1-a\right)\times \left(x^{d}/\left(b^{d}+x^{d}\right)\right)$$

where *x* is the nanoparticle dose, *y* is the normalized cell viability, *a* is the baseline viability, the maximal response was fixed as 1, *b* is the dose at half-maximal effect, and *d* is the slope parameter.

Metabolic cell stress endpoints capturing oxidative balance such as DCFDA transformation (reactive oxygen species (ROS) detection), lipid peroxidation (e.g., malondialdehyde (MDA)), and antioxidant enzyme activities, e.g., reduced glutathione (GSH), γ-glutamyl transferase (GGT), exhibited monotonic increases in response to increasing doses, reflecting redox imbalance and oxidative stress. The signal could theoretically increase with higher dosage, meaning the measured maximum signal may not represent the theoretically maximum value. These assays therefore lack a biologically fixed maximum and exhibit experiment-specific baselines. Genotoxicity endpoints were modeled analogously as they lack a biologically fixed maximum, but are retained as a mechanistically distinct category in Table 1 and Figure 3. Baselines and maximal values vary across experiments. We therefore used a four-parameter Hill function,^43^ allowing both the lower and upper asymptote to be estimated.

2 $$y=a\times \left[1+\left(c-1\right)\times \left(x^{d}/\left(b^{d}+x^{d}\right)\right)\right]$$

where *x* is the nanoparticle dose, *y* is the measured response on its assay-specific scale, *a* and *c* are the minimum and maximum response parameters, *b* is the potency parameter, and *d* is the slope.

Cytokine measurements, such as TNF-α, IL-6, and IL-1β, frequently show nonmonotonic (e.g., bell-shaped^44^) or threshold-triggered release profiles, and are often reported as fold change versus control. For these endpoints, classical sigmoidal fits were not imposed; instead, the toxicity threshold was reported as the lowest concentration producing a statistically significant change from control relative to a predefined benchmark, set here as a 2-fold change. The criterion was selected as a pragmatic threshold consistent with the positive-response criteria applied in the Nanotechnology Characterization Laboratory for nanoparticle immunotoxicity assessment^45^ and with common practice in cytokine-based evaluation.^46,47^

The classification of assay types and associated modeling strategies is summarized in Table 1. For endpoints modeled with Hill functions, points of departure were chosen using the benchmark dose (BMD), defined as the concentration differing by a specified percentage from the negative control response.^48^ Compared to the traditional NOAEL (no observed adverse effect level) approach used in risk assessment, the BMD method offers greater statistical robustness and better incorporates dose–response uncertainty.^49,50^ Prior studies have suggested that BMD for a 10% response (BMD_10_) is a suitable default indicator that avoids signal-to-noise ambiguities.^51^ We therefore choose BMD_10_ as the translated indicator, owing to its statistical stability and widespread use in screening-level risk assessment. Monte Carlo in R was used in the dose–response fitting process, with convergence assured using the common principle with r-hat smaller than 1.2.^52^ Fitting performance for each dose–response curve was measured between the data points and fitting curve through *R* ^2^.

Individual observations with biologically implausible values (e.g., cytotoxicity >100%) were removed prior to statistical analysis. Data sets were further screened for the following criteria: (i) response value filtering: at least one response shall be larger than 10% of change compared to negative control to ensure a reliable BMD_10_ value; (ii) informative sufficiency checking: data sets with inconsistent or noninformative response patterns were removed; (iii) excessive uncertainty: experiments with estimated BMD_10_ uncertainty spanning >3 orders of magnitude (e.g., median to lower credible bound exceeding 10^3^) were removed. This endpoint-specific strategy yields biologically interpretable, uncertainty-aware BMD_10_ estimates suitable for subsequent IVIVE translation.

To test whether experimental variables other than nanoparticle identity influenced the derived points of departure, extracted BMD_10_ values were stratified by cell line and exposure duration within each nanoparticle-assay combination, and an exploratory meta-regression was fitted on log_10_ BMD_10_ with cell line, assay category, and exposure duration as predictors.

### 2.3 PBPK Models and Benchmark Dosage Derivation

For translating *in vitro* doses to corresponding *in vivo* exposures, we used the previously developed mouse PBPK model,^42^ which treats the liver tissue as a single, homogeneous compartment and computes delivery efficiency (DE) over up to 168 h (fraction of the administered dose that reaches the organ). As a simplifying assumption, the *in vitro* dosage of nanoparticles was set equal to the unbound *in vivo* concentration in liver tissue. The plausibility of this assumption is evaluated by benchmarking IVIVE-translated mouse doses against independently derived *in vivo* reference values.

To match each *in vitro* experiment to a PBPK parameter set, we applied a nearest-neighbor approach. Entries were first filtered by nanoparticle core material, and then the entry with the smallest absolute difference in hydrodynamic diameter (or primary diameter when hydrodynamic size unavailable) was selected. This matching procedure was applied identically to both mouse and human *in vitro* experiments.

For the mouse, the matched PBPK parameter set directly provides a distribution of liver DEs. A corresponding human PBPK model was derived by substituting species-specific physiological parameters,^59–84^ while retaining kinetic-related parameters using the median value. The human PBPK model yields the median DE. We then derive the human DE percentiles by proportional scaling: the ratio between the human and mouse median DE was applied to the mouse percentiles to obtain the corresponding human percentiles. Detailed matching results for each *in vitro* experiment are provided in Tables S5 and S6, for mouse and human, respectively.

Assuming equivalence between *in vitro* concentration and *in vivo* liver tissue concentration, the dosage required to achieve the same liver concentration as observed *in vitro* can be expressed as

3 $$in\;vivo\;d\mathrm{osage}={\mathrm{Conc}}_{\mathrm{liver}}\times \frac{V_{\mathrm{liver}}}{{\mathrm{DE}}_{\mathrm{liver}}}={\mathrm{Conc}}_{\mathrm{cell}}\times \frac{V_{\mathrm{liver}}}{{\mathrm{DE}}_{\mathrm{liver}}}$$

where dosage is the input dosage to the organism (mg), *V*_liver_ is the liver volume (cm^3^), and DE_liver_ is the deposited fraction inside the liver given the input dosage (%). *In vitro* BMDs could be converted to equivalent *in vivo* administered doses (BMD_10_^IVIVE^) via eq 3 and then compared against BMD_10_^eq^ translated from the mouse *in vivo* data set.

To propagate uncertainty, we performed a two-stage Monte Carlo procedure. For each experiment, log-normal distributions were reconstructed from the 5th and 95th percentile quantiles of the Bayesian BMD_10_ posterior and the MCMC-derived DE_liver_ posterior of the matched PBPK experiment. Ten thousand independent draws were sampled from each distribution and combined via eq 3 to produce the joint BMD_10_^IVIVE^ distribution. Reported values are the median and the 50% (25th–75th) and 95% (2.5th–97.5th) credible intervals. For cytokine endpoints where LOEC-based point estimates were used instead of Bayesian fitting, the BMD_10_ was held constant and all propagated uncertainty derives from DE_liver_.

To assess the relative importance of each uncertainty source, a variance decomposition was performed by computing the variance of the log_10_-transformed BMD_10_ and DE_liver_ samples separately and expressing each as a percentage of their sum.

### 2.4 Mouse *In Vivo* Data Translation Procedure

The mouse *in vivo* toxicity data were compiled from multiple studies reporting heterogeneous toxicity threshold descriptors. To enable comparability across studies and alignment with *in vitro* IVIVE outputs, all endpoints were converted to BMD_10_ equivalents (BMD_10_^eq^) using a descriptor-specific uncertainty factor.^59^

4 $${\mathrm{BMD}}_{10}^{\mathrm{eq}}=\frac{\mathrm{reported}\;\mathrm{descriptor}}{\mathrm{uncertainty}\;\mathrm{factor}}$$

For each *in vivo* record, we assigned an uncertainty factor based on the reported descriptor, summarized in Table S17, and computed the BMD_10_^eq^ via the equation above.

The uncertainty factors in Table S17 reflect the expected quantitative relationship between each descriptor and BMD_10_. EC_10_, LC_10_, and IC_10_ correspond directly to a 10% effect magnitude (factor = 1). NOEC and HONEC represent the highest tested dose without a statistically significant effect, whose empirical comparisons have been shown to approximate the EC_10_ on average (factor = 1). LOEC denotes the lowest dose at which a significant effect was observed and typically falls between the EC_10_ and EC_25_. A factor of 2 was applied following ECHA guidance. For EC_25_–EC_50_ and LD_50_ values, a factor of 10 was applied based on empirical dose–response analyses showing that the ratio between EC_50_ and EC_10_ typically spans approximately 1 order of magnitude for sigmoidal concentration response curves.

This harmonization expresses diverse *in vivo* endpoints on a common scale across different studies, enabling direct comparison between *in vivo* and IVIVE-translated *in vitro* values.

To quantitatively assess the concordance between BMD_10_^IVIVE^ and BMD_10_^eq^, we computed three agreement metrics for each nanoparticle type: (i) the geometric mean (GM) fold difference, defined as the ratio of the GM BMD_10_^IVIVE^ to the GM BMD_10_^eq^ (values >1 indicate *in vitro* overestimation; <1 indicate more conservative *in vitro* predictions); (ii) coverage, the percentage of individual BMD_10_^IVIVE^ estimates falling within a factor of 10 and a factor of 3 of the *in vivo* BMD_10_^eq^, range; and (iii) the overall log_10_ root-mean-square deviation (RMSD) as a summary measure of average prediction deviation on a logarithmic scale.

Three dose metrics are distinguished in this study as follows throughout. BMD_10_ refers to the *in vitro* benchmark dose concentration (μg/mL) at which a 10% response change from the negative control is estimated from dose–response modeling. BMD_10_^IVIVE^ denotes the corresponding organism-level administered dose (mg/kg body weight) obtained by reverse translation of the *in vitro* BMD_10_ through the species-specific PBPK model. BMD_10_^eq^ refers to the harmonized *in vivo* benchmark dose equivalent, which serves as the comparator against which IVIVE predictions are evaluated.

## 3 Results

### 3.1 *In Vitro* Toxicity Data Set

For human cell studies, we identified a total of 138 dose–response cases from the literature. Based on study documentation and basic eligibility checks, 38 cases were excluded prior to dose–response modeling due to insufficient dose–response data points or the absence of a discernible toxicity trend, leaving 100 for modeling. Following dose–response modeling, post hoc quality control removed a further 30 cases in 3 successive steps: response filtering excluded 3 cytotoxicity data sets and 1 cytokine data set with all responses <10% change from the negative control (100 → 96); uncertainty checking excluded 13 data sets (1 metabolic cell stress, 12 cytotoxicity) with excessively wide BMD_10_ credible intervals (96 → 83); and informative sufficiency screening excluded 12 metabolic cell stress data sets with near-flat responses and 1 experiment with inconsistent response pattern (83 → 70).

For mouse cell studies, 93 mouse experiments were evaluated, of which 49 met basic eligibility after data cleaning. Postmodeling screening removed 11: response filtering excluded 4 cytotoxicity data sets (49 → 45); uncertainty checking excluded 4 cytotoxicity data sets (45 → 41); and informative sufficiency screening excluded 3 cytokine data sets (41 → 38). Overall, 70 human and 38 mouse data sets were retained for the subsequent analyses, and the full screening workflow with data set counts is summarized in Figure S1. A detailed breakdown of all compiled experiments, including case identifiers and the specific filtering criterion applied at each stage, is provided in Tables S8 and S9 for mouse and human. Fe_2_O_3_ was included in the initial data collection but did not survive the quality-control workflow; therefore, it was excluded from all subsequent analyses.

The human data included 26 metabolic cell stress, 29 cytotoxicity, 3 cytokine, 3 genotoxicity assays, and 9 LDH assays. The mouse data comprised 16 cytotoxicity, 20 cytokine, and 2 metabolic cell stress assays. Retained experiments were unevenly distributed across the nanoparticle types. The human data set was dominated by SiO_2_ (*n* = 34) and TiO_2_ (*n* = 19), with smaller representation from Au (*n* = 13) and GO (*n* = 4). Similarly, the mouse data set was heavily skewed toward TiO_2_ (*n* = 23) and SiO_2_ (*n* = 13), with only two experiments evaluating GO. A full cross-tabulation by nanoparticle type and assay category is provided in Table S10.

### 3.2 *In Vitro* Dose–Response Curve Generation and *In Vitro* BMD_10_ Estimation

Across mouse and human cell systems, the fitted dose–response models showed good agreement with the observed data, as shown in Figure 1, with experiment *R* ^2^ ranging from 0.73 to 0.99 for mouse and 0.58 to 0.99 for human data sets (reported in Tables S3 and S4). One example of a fitted dose–response curve is provided in Figure 1A; the remaining ones are provided in the Supporting Information from Figures S2–S10 for human *in vitro* experiments and Figures S11–S15 for mouse *in vitro* experiments. Convergence was confirmed for every experiment using the calculated r-hat values (Table S7).

**Figure 1:**
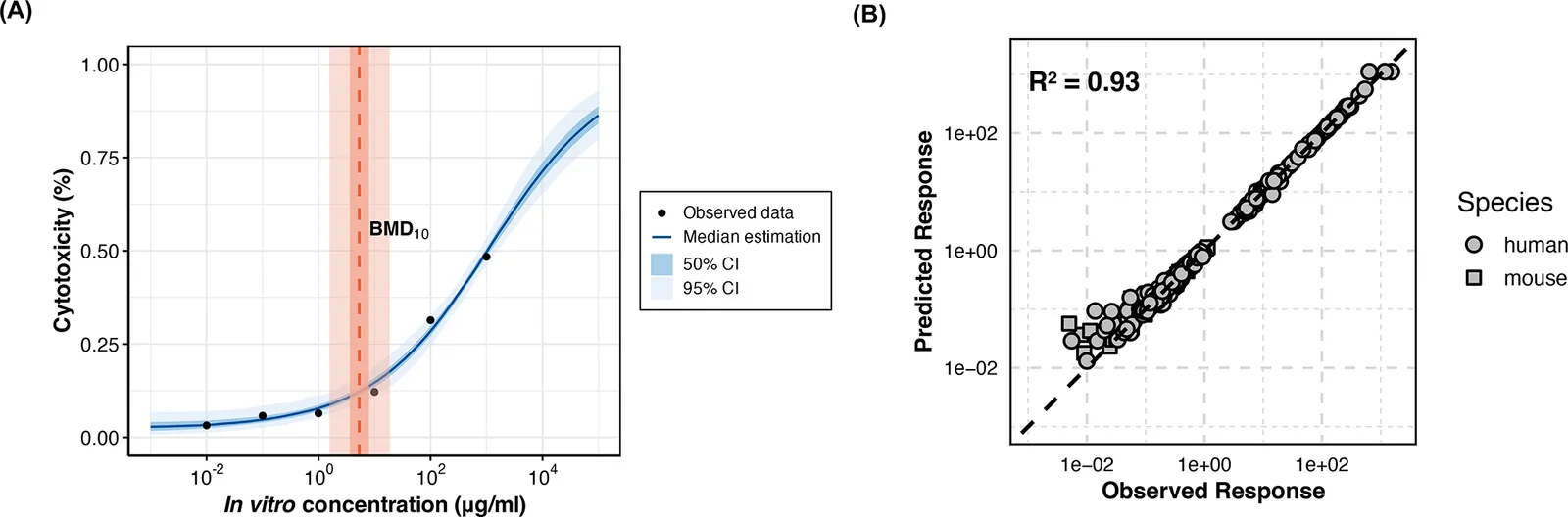
(A) A mouse cytotoxicity experiment (case 13) as an example showing the dose–response model fitting, with the 50 and 95% credible intervals for the fitted curve and the corresponding BMD_10_ shown as a ribbon. The median fitted value is represented by a line, and dots represent the observed data points. (B) Goodness-of-fit evaluation for dose–response models fitting across all *in vitro* toxicity experiments in human and mouse cell systems.

BMD_10_ values extracted from each experiment are provided in Tables S3 and S4 for mouse and human data sets, and for cytokines, Tables S11 and S12. The human and mouse BMD_10_ values are shown in Figure 2, stratified by experiment identity (nanoparticle core, assay, and cell line) and colored by assay category, with box-whisker plots summarizing the distribution of experiment-level medians. The spread covers both differences in experimental heterogeneity (e.g., hydrodynamic size, exposure duration) as well as endpoint type (LDH, NRU, MTT, etc.). Individual experiment-level credible intervals are provided in Figure S16 (human) and Figure S17 (mouse). Cytokine outcomes are shown in the plot as well for completeness, where classical dose–response curve fitting was unsuitable, we used the minimum effective concentration (i.e., LOEC-based equivalent) in place of the BMD_10_.

**Figure 2:**
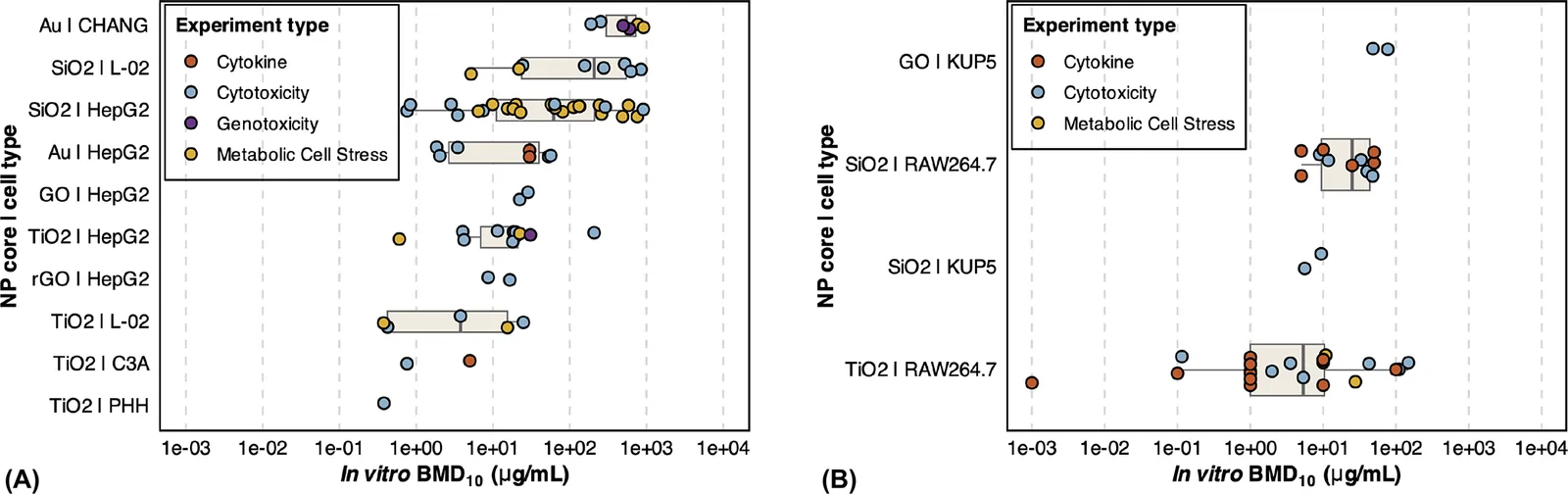
Distribution of BMD_10_ values (μg/mL) across nanoparticle types in (A) human cell-based experiments and (B) mouse cell-based experiments. Each point represents a median BMD_10_ estimate from a fitted dose–response curve for an individual experiment, colored by assay category. Boxes summarize the distribution of these experiment-level medians within each group (box = interquartile range, central line = median, whiskers = most extreme value within 1.5× interquartile range). Groups with fewer than three experiments are shown as points only.

The overall ranges in mouse cell systems overlap those observed in human systems, with material- and assay-dependent shifts evident for several nanoparticles. Across nanoparticles, TiO_2_ exhibits lower BMD_10_ values than those of several other nanoparticles, indicating higher apparent toxicity. However, we did not observe a clear distinction between different cell types in Figure 2.

### 3.3 *In Vitro* BMD_10_ Analysis

To evaluate particle size as a determinant of *in vitro* toxicity, we analyzed the relationship between nanoparticle size and BMD_10_. Metabolic cell stress readouts were excluded due to heterogeneous measurement scales across assays. Meanwhile, cytotoxicity endpoints were considered here since metrics are the same as cell viability and directly comparable across assays. We focused on this combination (TiO_2_ in HepG2 cytotoxicity experiments), which provides the largest data set (eight experiments). These eight experiments derive from three independent publications, each of which used a single particle size (hydrodynamic diameter 57.3 nm, *n* = 3 experiments; 192.5 nm, *n* = 4; 701 nm, *n* = 1), differing in exposure duration or assay type. Figure 3 shows experiment-level BMD_10_ against particle hydrodynamic size on a log_10_ scale, with each point identified by its source publication and the number of experiments annotated per size. We observe an upward trend in BMD_10_ with increasing size, consistent with smaller TiO_2_ particles exhibiting greater toxicity (slope of log_10_ BMD_10_ on diameter = 0.0023 per nm; nominal 95% CI 0.0013–0.0033; nominal *p* = 0.002; *R* ^2^ = 0.83; Spearman ρ = 0.73), a direction also reported in dedicated experimental studies.^65^ However, because nanoparticle size cannot be separated from study identity at this data set size, these data are consistent with, but do not establish, an association between particle size and BMD_10_. No inference about size as a causal determinant of toxicity can be drawn from this compilation, and the analysis is presented as hypothesis-generating.

**Figure 3:**
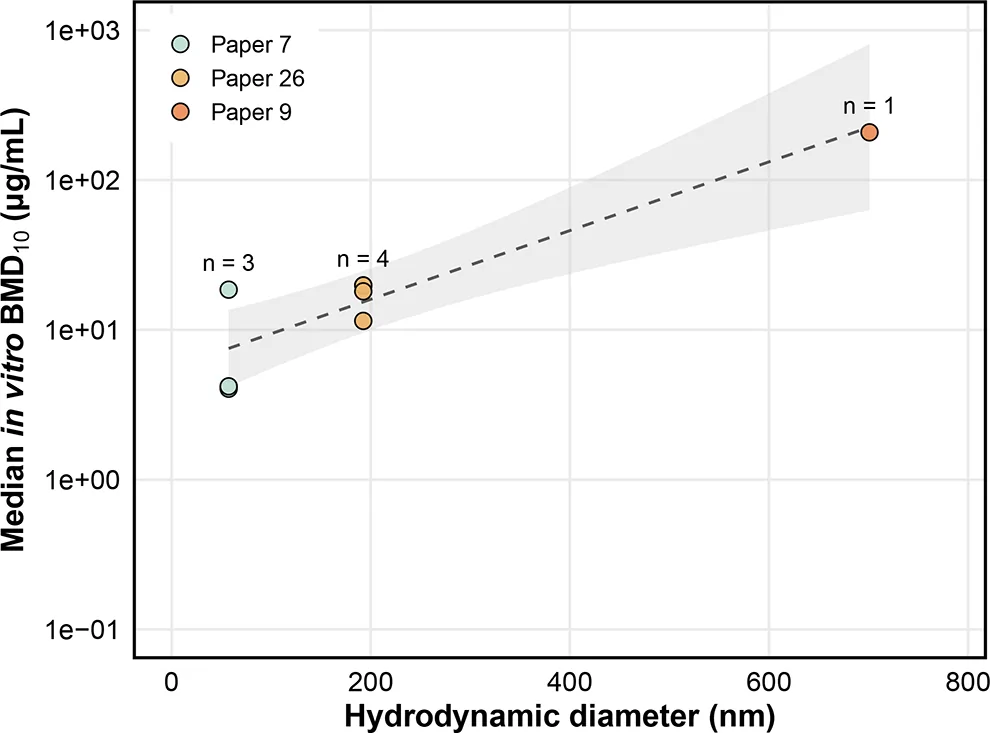
Relationship between nanoparticle size and *in vitro* BMD_10_ values in experiment sets with TiO_2_, cytotoxicity, and HepG2 cells (*n* = 8 experiments from three publications). Scatter plot showing the association between average nanoparticle size and BMD_10_. The regression line (dashed black) with 95% confidence interval (gray band) is fitted to log_10_-transformed BMD_10_ values versus nanoparticle diameter. Each point represents a single experiment and corresponds to the median BMD_10_ obtained from Bayesian dose–response modeling; colors denote the source publication (paper IDs and full references in Table S2).

A parallel SiO_2_–HepG2 cytotoxicity data set had the same nominal sample size but contained only two reported sizes (70 and 1200 nm), insufficient for bin-wise comparison, but showed the same qualitative pattern (larger particles lead to higher BMD_10_) with smaller size having BMD_10_ below 10 and larger size having BMD_10_ with 10^2^ magnitudes.

Stratification by cell line and exposure duration showed no consistent directional differences between cell models (Figure S20, Table S13). In the exploratory meta-regression (Table S14), neither cell line (all *p* > 0.09) nor assay category (all *p* > 0.28) was a significant predictor of BMD_10_; only exposure duration (*p* = 0.007) and CHANG liver cells (*p* = 0.004, *n* = 6) reached significance, and the explanatory power was limited (adjusted *R* ^2^ = 0.37). This reflects the covariation of cell line, particle size, exposure duration, and assay type in a literature-compiled data set, where these factors cannot be varied independently. The observed variability in BMD_10_ should therefore be interpreted as an inherent feature of cross-study compilation rather than being attributed to any single experimental factor.

### 3.4 Mouse *In Vivo* Toxicity Data Translation as IVIVE Benchmark

We compiled mouse *in vivo* toxicity studies from the literature and summarized study characteristics in Table S18. Most *in vivo* studies reported acute administered doses or lethal metrics (LD_50_) rather than a full concentration–response series. Extracted endpoints included hepatic metabolism changes (AST, SOD), immune marker (CD68), cytokines (IL-6, IL-8) released in the liver, and mortality. Owing to the limited availability of liver-related endpoints, we also included reproductive outcomes.

Reported administered metrics were classified by the reported descriptor as NOEC (no observed effect concentration), LOEC (lowest observed effect concentration), LD_50_ (lethal dose at 50), and HONEC (highest observed no-effect concentration). We used LOEC to denote studies in which only a single dose was tested, and an effect was observed, precluding categorization as neither NOEC nor a conventional LOEC. To enable comparability with IVIVE-derived toxicity thresholds (BMD_10_^IVIVE^), all descriptor types were converted into *in vivo* BMD_10_^eq^ using uncertainty factors (Table S17). In total, 27 *in vivo* data points were harmonized to BMD_10_^eq^ and are reported in Table S18.

### 3.5 Dosing Conversion from Mouse *In Vitro* and IVIVE Pathway Validation

We converted mouse *in vitro* BMD_10_ values (μg/mL) into organism-level doses BMD_10_^IVIVE^ values (mg/kg) by the PBPK-derived delivery efficiency and compared these BMD_10_^IVIVE^ with *in vivo* BMD_10_^eq^ values as the validation for our IVIVE pathway (details reported in Table S5).

As shown in Figure 4, the BMD_10_^IVIVE^ generally align with BMD_10_^eq^ across materials, supporting the consistency of the IVIVE translation from *in vitro* concentrations to *in vivo* equivalent doses. In general, *in vitro* BMD_10_^IVIVE^ values derived from cytokine or metabolic cell stress endpoints tended to be lower than those from cytotoxicity assays, whereas the cytotoxicity-based translated value aligned more closely with *in vivo* BMD_10_^eq^.

**Figure 4:**
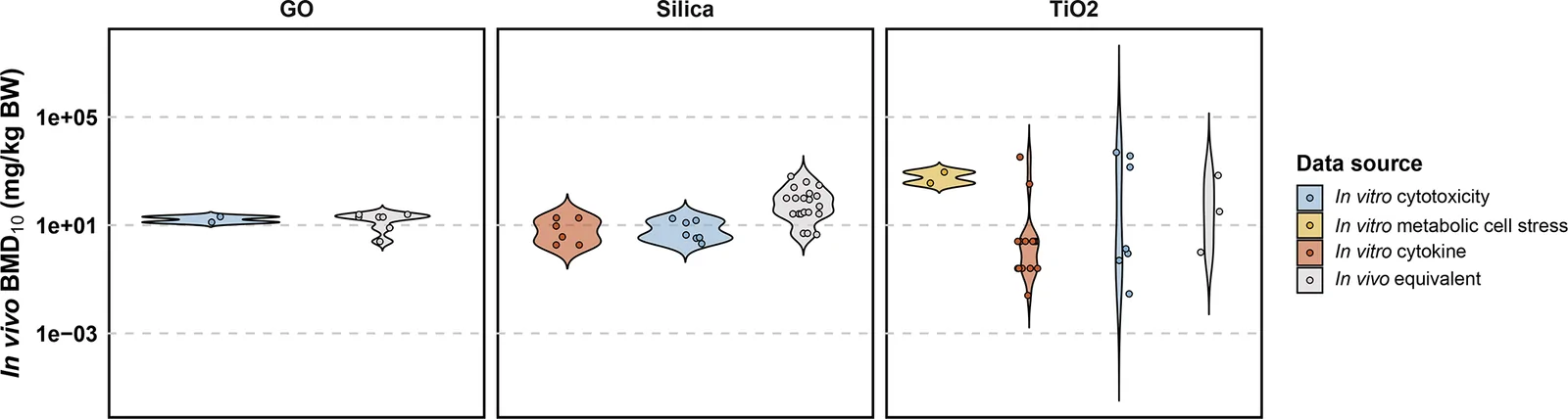
Distribution of *in vivo* BMD_10_ from different data sources by nanoparticle type. Small circles indicate individual experiments. Gray color represents translated BMD_10_^eq^ from the literature *in vivo* studies, while the other colored points represent *in vivo* BMD_10_^IVIVE^ translated from *in vitro* BMD_10_ using the PBPK model.

To quantify the agreement, we computed GM fold differences and coverage metrics for each nanoparticle (Table S18). GO showed close agreement with a GM fold difference of 1.3. For SiO_2_ and TiO_2_, BMD_10_^IVIVE^ were approximately 8-fold lower than *in vivo* references, indicating that the *in vitro*-based pathway yields more conservative estimates, consistent with the predominance of more sensitive endpoints (cytokine, metabolic stress) *in vitro* compared to mortality-based endpoints *in vivo*. Despite these shifts, all GO (2/2) and SiO_2_ (13/13) BMD_10_^IVIVE^ estimates, and 87% (20/23) of TiO_2_ estimates, fell within 1 order of magnitude of the *in vivo* BMD_10_^eq^ range.

GO exhibits the closest agreement between BMD_10_^IVIVE^ and BMD_10_^eq^, which could be explained by the reason that *in vitro* and *in vivo* GO particle sizes both clustered around ∼500 nm. The larger discrepancies were observed for SiO_2_, where *in vivo* translated BMD_10_^eq^ yields higher values. This divergence can be explained by mismatched size distributions between data sources (*in vitro* predominantly <20 nm vs *in vivo* spanning ∼20–1000 nm) and differences in assays (*in vivo* mostly focusing on mortality while *in vitro* data were cytokine and metabolic cell stress endpoints). A similar pattern was observed for TiO_2_.

A variance decomposition of the total BMD_10_^IVIVE^ uncertainty (Tables S15 and S16) shows that the relative contributions of *in vitro* dose–response and PBPK delivery efficiency uncertainty are primarily determined by endpoint type. For human experiments, the two sources contributed approximately 59 and 41% on average; for mouse experiments, PBPK delivery efficiency dominated overall (∼100%), reflecting the higher proportion of cytokine endpoints for which LOEC-based point estimates carry no posterior spread. When restricted to cytotoxicity endpoints, both species showed BMD_10_ uncertainty as the dominant contributor.

Overall, the pattern supports qualitative concordance between *in vitro*-translated and *in vivo*-derived effective dosages, with the IVIVE pathway tending toward conservative estimates. Residual gaps are attributable to particle size distributions and endpoint composition, underscoring the value of matched characterization and assay context for quantitative IVIVE.

### 3.6 Predicted Safety Limits for Human

We calculated the human BMD_10_^IVIVE^ (mg/kg) by translating human *in vitro* experiments through the human PBPK model, as shown in Figure 5. Individual experiment-level credible intervals of the translated safety limits are provided in Figure S18, faceted by nanoparticle and cell type. The corresponding values are summarized in Table S6. GO estimates cluster around 10 mg/kg, but this material is represented by only two experiments, and the apparent clustering should therefore be interpreted with caution, given the limited sample size. In contrast, SiO_2_ and TiO_2_ span a substantially wider range of predicted BMD_10_^IVIVE^ values (around 10^–1^–10^3^ mg/kg), consistent with the greater heterogeneity of the underlying *in vitro* experiments, including diverse cell types and a broad span of particle sizes.

**Figure 5:**
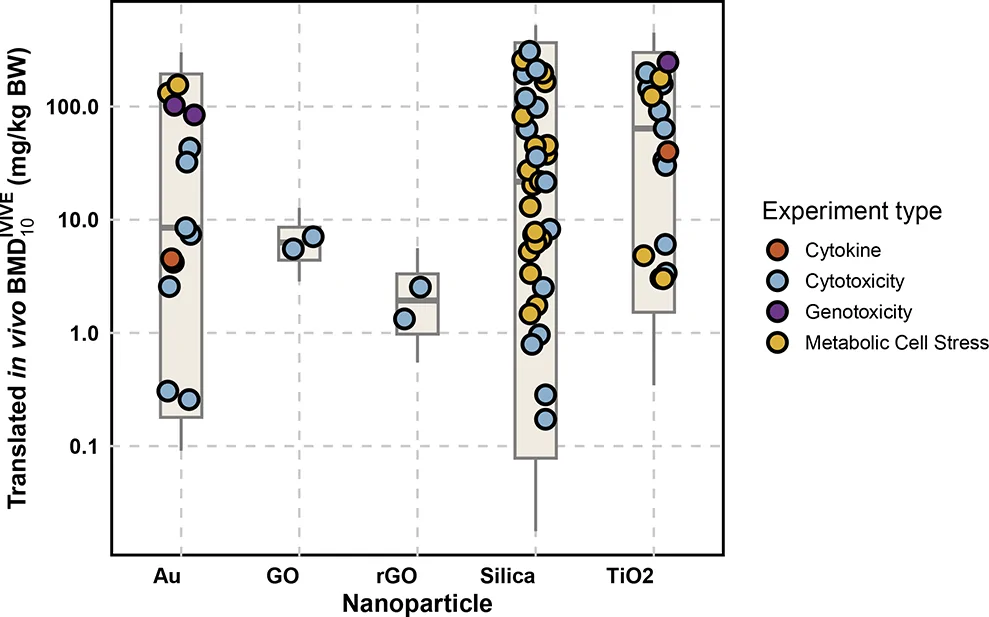
Estimated human *in vivo* BMD_10_^IVIVE^ values (mg/kg BW (body weight)) derived from *in vitro* experiments for different nanoparticles. Points represent the median estimated dose for each experiment, the box spans the 25th–75th percentile, and the vertical line marks the median of the distribution of estimates across all experiments, with whiskers extending to the 5th and 95th percentiles.

For Au, three low-end estimates are tightly clustered around 0.25 mg/kg and overlap visually in Figure 5, corresponding to particle diameters of 10–40 nm. Higher Au BMD_10_^IVIVE^ estimates are associated with larger particles (e.g., 160 nm hydrodynamic diameter). For TiO_2_, the highest values, particularly within cytotoxicity assays, correspond to larger diameters (200 and 700 nm). A similar size dependence is observed for SiO_2_, where elevated BMD_10_^IVIVE^ estimates are primarily linked to the largest particles (1245 nm). These trends are consistent with the size–toxicity relationships observed in the *in vitro* data set.

We surveyed the literature to identify human studies that could contextualize plausible systemic exposure levels for nanoparticles. For gold, clinical prostate cancer studies of AuroShell (gold-silica nanoshells, a 120 ± 12 nm silica core with a 12–15 nm gold shell) report intravenous dosing 22–36 mg/kg BW^83^ without serious adverse events. Although AuroShell is a composite material rather than pure Au nanoparticles, and the study reports general tolerability rather than liver-specific endpoints, this material is of comparable scale to the particles in our data set, similar in magnitude to our largest Au particles (∼160 nm hydrodynamic diameter) and within our SiO_2_ size range (20–200 nm), and given its composite structure, it is relevant to both nanoparticle types. The reported dose falls within the range of our estimated value for Au and silica nanoparticles.

For the other nanoparticles considered here, we did not identify peer-reviewed reports providing human injection doses expressed as particle mass. Human intravenous studies exist, but doses are typically reported as radioactivity,^84,85^ and other clinical and consumer-relevant applications use oral delivery.^86,87^ We found no peer-reviewed reports of human injection dosing for TiO_2_ nanoparticles. And the most relevant human evidence for graphene oxide is a first-in-human controlled inhalation study (200 μg/m^3^ for 2 h in 14 healthy volunteers).^88^

Although direct validation against human data is not possible for these materials, regulatory reference values do exist. For TiO_2_, the EFSA identified a NOAEL of 2250 mg/kg body weight per day based on an oral rat study for E171 (food-grade TiO_2_).^89^ For occupational inhalation exposure, NIOSH recommends a limit of 0.3 mg/m^3^ for ultrafine TiO_2_ (<100 nm) and 2.4 mg/m^3^ for fine TiO_2_.^90^ Our injection-derived BMD_10_^IVIVE^ values for TiO_2_ span approximately 1–10^3^ mg/kg, which, as expected, are substantially lower than oral reference doses since intravenous administration bypasses absorption barriers.

## 4 Discussion

This work establishes an uncertainty-aware IVIVE workflow that translates liver *in vitro* nanotoxicity data to safety dosages for organisms using PBPK modeling. We curated and quality-controlled a cross-species *in vitro* data set (70 human and 38 mouse toxicity experiments) and harmonized heterogeneous mouse *in vivo* evidence to a common benchmark metric, enabling systematic comparison across data sources. Endpoint-specific dose–response models yielded *in vitro* BMD_10_ values with Bayesian uncertainty propagation. These values were then mapped to liver tissue concentrations using species-specific PBPK models and translated into corresponding external safety dose estimates for organisms (BMD_10_^IVIVE^), providing a “from plate to organism” route consistent with nonanimal methods and SSbD principles.

Analysis of the *in vitro* experiments revealed several recurring patterns. First, TiO_2_ generally showed lower BMD_10_ values than other nanoparticles, indicating higher *in vitro* toxicity. Second, particle size covaried with *in vitro* toxicity across the few available studies. In the TiO_2_–HepG2 cytotoxicity experimental data set, the BMD_10_ increased with size, consistent with smaller particles exhibiting greater toxicity. A comparable SiO_2_–HepG2 data set lacked sufficient size resolution for bin-wise analysis but showed the same trend. This trend should be interpreted cautiously, given the small sample size and potential confounding by covarying physicochemical properties. We did not observe a consistent ranking of apparent sensitivity by cell system or species. This likely reflects the dominance of liver-relevant cell systems (e.g., HepG2, L-02, Kupffer cells), which share detoxification and defense functions and are commonly used in hepatoxicity assessment,^91^ thereby potentially reducing systematic differences to cell identity alone. However, a limitation is the low representation of primary human hepatocytes (single experiment), precluding isolation of cell-type effects due to covariation with experimental parameters. However, as nanoparticle toxicity here is largely metabolism-independent, differences in cellular metabolic capacity should minimize overall impact.

When converted to organism-level doses using PBPK-derived delivery efficiencies, mouse *in vitro*-translated BMD_10_^IVIVE^ values generally aligned with harmonized *in vivo* reference points (BMD_10_^eq^). The agreement was strongest for graphene oxide, where *in vitro* and *in vivo* size ranges closely matched (around 500 nm). Larger discrepancies for silica could be traced to mismatches in size distributions (*in vitro* predominantly <20 nm and *in vivo* spanning 20–1000 nm). Stratifying data sets by diameter (≤50 vs >50 nm; Figure S19) showed closer agreement with size-comparable particles, supporting size as a primary driver of the discrepancy and consistent with the TiO_2_ size–toxicity relationship (Figure 3).

A key challenge lies in the fragmentation and incompleteness of the available literature data. In the collected data set, 33 of 72 human *in vitro* records lacked hydrodynamic size, and we needed to use primary diameter as a replacement; ζ potential and detailed dose-rate information were often missing. These gaps necessitated the nearest-neighbor matching to estimate PBPK delivery efficiency rather than regenerating a full PBPK model using the multilinear regression as stated in the previous work.^42^ The PBPK parameter library currently contains only 18 entries, with as few as two for some core materials (e.g., TiO_2_, Fe_2_O_3_), and only core material and diameter are used as matching criteria due to incomplete physicochemical reporting in the literature. LOEC-based surrogate conversions enable harmonization to a common scale but inherently carry a wider uncertainty than direct BMD estimates. On the benchmarking side, the mouse *in vivo* data set comprised only 27 data points across four nanoparticle types, differing in size, surface modification, endpoint, and dosing protocol, whose harmonization used uncertainty factors.

Model assumptions introduce further uncertainty. The liver was treated as a single well-mixed compartment in our model, without explicit representation of suborgan heterogeneity. Besides, the model equates nominal *in vitro* well concentrations with unbound *in vivo* liver concentrations. This assumption neglects delivered dose mechanics (could be addressed by tools like ISDD^92^ or DG models,^93^ but require effective-density and agglomeration data) alongside protein corona differences between culture media and plasma, and potential ion-dissolution contributions. Furthermore, restricted to acute *in vitro* exposures (6–72 h) and single-dose simulations (≤168 h), the framework misses long-term tissue accumulation under realistic chronic scenarios. Lastly, hormetic or biphasic responses are not captured by the Hill model and could lead to underestimation of low-dose effects.

These constraints suggest clear opportunities for strengthening the IVIVE pathways. On the data side, adopting minimal reporting standards,^94–96^ covering primary size, hydrodynamic size, ζ-potential under assay conditions, exposure duration, and relatively resolved delivered dose range, would reduce data removal and shrink uncertainty. On the modeling side, extending the PBPK structure to include liver subcompartments and explicit treatment of protein corona dynamics and dissolution kinetics, along with calibration against cross-study data sets, would improve generalizability and predictive confidence. Extension to inhalation or oral routes could be implemented through addition of a route-specific absorption module (e.g., MPPD^97^ or a gastrointestinal model^98,99^ ) upstream of the systemic PBPK model, leaving downstream IVIVE translation unchanged.

In summary, our integrated dosimetry-PBPK IVIVE workflow delivers an uncertainty-aware translation from *in vitro* experiments to organism-relevant exposure guidance. Its practical value lies in standardization and scalability: rather than a single material-specific demonstration, it provides a reproducible route to place heterogeneous *in vitro* data sets on a common BMD_10_^IVIVE^ scale. The derived values represent points of departure rather than definitive regulatory limits, but they offer practical entry points for SSbD decision making, comparative ranking of candidate materials during early-stage design, flagging of low BMD_10_^IVIVE^ materials for closer scrutiny or reformulation, and screening-level hazard banding within tiered assessment schemes. Translating them into formal safety limits would require additional assessment factors, route-specific PBPK extensions, and alignment with regulatory frameworks, which remain as directions for future work. Nevertheless, by providing a reproducible, uncertainty-aware route from bench data to organism-relevant dose estimates, the framework offers a practical step toward reducing the reliance on new animal testing in nanoparticle hazard assessment.

## Supplementary Material





















## Data Availability

The analysis code supporting this study, including the dose–response fitting, Bayesian model implementation, and *in vitro* *in vivo* extrapolation routines, is openly available at https://github.com/jimengwu/In-vitro-in-vivo-extrapolation-for-liver-toxicity.git. All underlying *in vitro* and *in vivo* toxicity data are provided in the Supporting Information.

## Notes

Cross-tabulation of retained experiments by nanoparticle type, assay category and species (Table S10); cytokine LOEC values under 1.9- and 2.0-fold change criteria (Tables S11 and S12); human BMD_10_ summary by nanoparticle, assay, cell type and exposure duration (Table S13); multiple linear regression coefficients for human BMD_10_ (Table S14); variance decomposition of BMD_10_^IVIVE^ uncertainty (Tables S15 and S16); uncertainty factors for conversion of dose descriptors to BMD_10_^eq^ (Table S17); compiled mouse *in vivo* toxicity data set for injection exposure (Table S18); quantitative agreement between mouse BMD_10_^IVIVE^ and *in vivo* BMD_10_^eq^ (Table S19); screening and quality-control workflow (Figure S1); fitted dose–response curves for retained and excluded human and mouse *in vitro* experiments (Figures S2–S9 and S11–S14); cytokine endpoint data (Figures S10 and S15); experiment-level BMD_10_ credible intervals for human and mouse data sets (Figures S16 and S17); experiment-level human BMD_10_^IVIVE^ credible intervals (Figure S18); *in vivo* BMD_10_ distributions stratified by primary diameter (Figure S19); and subgroup analysis of human *in vitro* BMD_10_ (Figure S20) (PDF)
Raw *in vitro* toxicity data for mouse liver cells (Table S1) (XLSX)
Raw *in vitro* toxicity data for human liver cells (Table S2) (XLSX)
Mouse BMD fitting results after postmodeling filtering (Table S3) (XLSX)
Human BMD fitting results after postmodeling filtering (Table S4) (XLSX)
Translated *in vivo* BMD_10_ doses derived from mouse *in vitro* fits by *in vitro* *in vivo* extrapolation (Table S5) (CSV)
Translated *in vivo* BMD_10_ doses derived from human *in vitro* fits by *in vitro* *in vivo* extrapolation (Table S6) (CSV)
MCMC convergence diagnostics for the Bayesian dose–response models (Table S7) (CSV)
Screening summaries for all compiled mouse experiments (Table S8) (CSV)
Screening summaries for all compiled human experiments (Table S9) (CSV)
