# Supplementary material for: Deriving Organism-Relevant Nanoparticle Safety Exposure Limits from In Vitro Toxicity Data
Source: Environ Sci Technol. 2026 Oct 28;60(44):31382–93. doi: 10.1021/acs.est.6c13254 (PMC13757938; doi:10.1021/acs.est.6c13254)
Supplement: Supplementary file 1 [file es6c13254_si_001.pdf]

Deriving organism-relevant nanoparticle safety exposure limits from *in vitro* toxicity data

Jimeng Wu[a,b], Peter Wick[a], Bernd Nowack[b*]

[a] Empa, Swiss Federal Laboratories for Materials Science and Technology, Nanomaterials in Health Laboratory, Lerchenfeldstrasse 5, 9014 St. Gallen, Switzerland

[b] Empa, Swiss Federal Laboratories for Materials Science and Technology, Technology and Society Laboratory, Lerchenfeldstrasse 5, 9014 St. Gallen, Switzerland

* Corresponding author, nowack@empa.ch

Supporting Information

Supporting information table caption (uploaded separately as xls files)

Tables S1. Raw *in vitro* toxicity data for mouse liver cells compiled from the literature. Columns include Paper_ID, DOI, nanoparticle core, hydrodynamic and primary size ranges, shape, zeta potential, exposure time, cell type, culture medium, tested concentration amout, assay, and measured outcomes.

Table S2. Raw *in vitro* toxicity data for human liver cells compiled from the literature. Column definitions are the same as in Table S1.

Table S3. Benchmark dose (BMD) fitting results for mouse datasets after post-modelling filtering. Results from dose–response curve fitting are provided and include Paper_ID, nanoparticle core, assay, hydrodynamic diameter, exposure time, shape, average diameter, zeta potential, cell type, medium type, fitted model parameters, fitted BMD estimates, and R^2.

Table S4. BMD fitting results for the human datasets after post-modelling filtering. Column definitions are the same as in Table S3.

Table S5. Translated *in vivo* BMD_{10} doses from mouse *in vitro* fitted BMD results. Columns include CASE_ID, Paper_ID, nanoparticle properties, and the estimated *in vivo* dosage corresponding to extracted *in vitro* BMD_{10} value, reported as the 5th, 25th, 50th, 75th, and 95th percentiles.

Table S6. Translated *in vivo* BMD_{10} doses from human *in vitro* fitted BMD results. Column definitions are the same as in Table S5.

Table S7. MCMC convergence diagnostics ($\hat{R}$, ESS_bulk, ESS_tail) for each parameter of the Bayesian dose–response model for human and mouse *in vitro* experiments.

Table S8. Summary of all compiled mouse *in vitro* experiments with case identifiers, nanoparticle type, cell line, assay category, and the screening stage at which each dataset was retained or excluded. Filtering criteria correspond to the workflow described in Figure 1: pre-modeling (sufficient data points) and post-modeling (response filtering, uncertainty checking, and informative sufficiency).

Table S9. Summary of all compiled human *in vitro* experiments with case identifiers, nanoparticle type, cell line, assay category, and the screening stage at which each dataset was retained or excluded. Filtering criteria correspond to the workflow described in Figure 1: pre-modeling (*sufficient* data points) and post-modeling (response filtering, uncertainty checking, and informative sufficiency).

Table S10. Breakdown of the retained *in vitro* dose–response experiments by nanoparticle type, assay category and species. Numbers indicate the experiments passing the complete screening and quality-control workflow, reported separately for mouse and human cell systems. Fe_2O_3 is not shown, as no dataset survived the quality-control workflow.

Species	Assay category	TiO_2	SiO_2	Au	GO	Total
**Mouse**	Cytotoxicity (incl. LDH)	7	7	–	2	16
	Metabolic cell stress	2	–	–	–	2
	Cytokine	14	6	–	–	20
	Genotoxicity	–	–	–	–	–
	Subtotal	**23**	**13**	**–**	**2**	**38**
**Human**	Cytotoxicity (incl. LDH)	13	14	7	4	38
	Metabolic cell stress	4	20	2	–	26
	Cytokine	1	–	2	–	3
	Genotoxicity	1	–	2	–	3
	Subtotal	**19**	**34**	**13**	**4**	**70**

Table S11. Cytokine LOEC values derived with 1.9-fold and 2.0-fold change criteria for the mouse *in vitro* experiments.

Study	LOEC_1.9	LOEC_fold_1.9	LOEC_2	LOEC_fold_2	Note
1	25	2	25	2	
2	50	2.028	50	2.028	
3	no_toxic	NA	no_toxic	NA	
4	50	4.57	50	4.57	
5	5	2.231	5	2.231	
6	10	2.752	10	2.752	
7	5	2.47	5	2.47	
8	0.001	1.951	10	2.296	No intermediate concentrations between 0.001 and 10
9	no_toxic	NA	no_toxic	NA	
10	no_toxic	NA	no_toxic	NA	
11	100	2.06	100	2.06	
12	0.1	2.292	0.1	2.292	
13	1	4.364	1	4.364	
14	1	3.651	1	3.651	
15	10	3.735	10	3.735	
16	10	2.954	10	2.954	
17	10	4.103	10	4.103	
18	1	2.847	1	2.847	
19	1	3.385	1	3.385	
20	1	1.905	10	5.556	No intermediate concentrations between 1 and 10
21	10	4.739	10	4.739	
22	10	3.277	10	3.277	
23	1	2.216	1	2.216	

Table S12. Cytokine LOEC values derived with 1.9-fold and 2.0-fold change criteria for the human *in vitro* experiments.

Study	LOEC_1.9	LOEC_fold_1.9	LOEC_2	LOEC_fold_2	Note
1	2.5	1.9	5	2.2	No intermediate concentrations between 2.5 and 5
2	80	1.9	no_toxic	NA	No concentration exceeds 2-fold threshold
3	30	2.4	30	2.4	
4	30	2.1	30	2.1	

Table S13. Human *in vitro* BMD_{10} summary by nanoparticle, assay category, cell type and exposure-duration bin. Values report the number of contributing experiments, the range of primary and hydrodynamic particle diameters (nm), the number of experiments with measured hydrodynamic size, and the median experiment-level BMD_{10} (μg/mL) within each group.

NP	general_experiment_type	Cell_type	exposure_bin	n_experiments	size_median	size_min	size_max	HD_median	HD_min	HD_max	n_HD_available	BMD10_median
Au	Biochemical	CHANG	≤24 h	2	55	55	55	160	160	160	2	844.59
Au	Cytokine	HepG2	25-48 h	2	17.5	10	25	NA	Inf	NA	0	30
Au	Cytotoxicity	CHANG	≤24 h	2	55	55	55	160	160	160	2	221.45
Au	Cytotoxicity	HepG2	25-48 h	5	25	10	40	NA	Inf	NA	0	3.47
Au	Genotoxicity	CHANG	≤24 h	2	55	55	55	160	160	160	2	550.47
GO	Cytotoxicity	HepG2	≤24 h	2	40	40	40	297.1	297.1	297.1	2	25.48
SiO2	Biochemical	HepG2	>48 h	10	20	20	20	1245	1245	1245	10	133.48
SiO2	Biochemical	HepG2	≤24 h	8	80	21.1	199.9	87.8	37.8	203.3	8	19.17
SiO2	Biochemical	L-02	≤24 h	2	51.7	51.7	51.7	71.7	71.7	71.7	2	13.51
SiO2	Cytotoxicity	HepG2	>48 h	5	20	20	59.7	1245	70.1	1245	4	63.56
SiO2	Cytotoxicity	HepG2	≤24 h	3	59.7	40.3	81	70.1	70.1	70.1	1	0.84
SiO2	Cytotoxicity	L-02	≤24 h	6	48.6	21.2	86.4	71.7	71.7	71.7	1	399.9
TiO2	Biochemical	HepG2	25-48 h	1	42.3	42.3	42.3	NA	Inf	NA	0	22.34

TiO2	Biochemical	Hep G2	>48 h	1	50	50	50	701	701	701	1	1.77
TiO2	Biochemical	Hep G2	≤24 h	1	50	50	50	192.5	192. 5	192. 5	1	0.6
TiO2	Biochemical	L-02	≤24 h	2	6.1	3.8	8.5	NA	Inf	NA	0	7.92
TiO2	Cytokine	C3A	≤24 h	1	240	240	240	287	287	287	1	5
TiO2	Cytotoxicity	C3A	≤24 h	1	240	240	240	287	287	287	1	0.76
TiO2	Cytotoxicity	Hep G2	25-48 h	3	50	30	50	192.5	57.3	192. 5	3	11.47
TiO2	Cytotoxicity	Hep G2	>48 h	2	40	30	50	379.1	57.3	701	2	106.23
TiO2	Cytotoxicity	Hep G2	≤24 h	3	50	30	50	192.5	57.3	192. 5	3	19.78
TiO2	Cytotoxicity	L-02	≤24 h	3	3.8	3.8	8.5	NA	Inf	NA	0	3.79
TiO2	Cytotoxicity	PHH	≤24 h	1	240	240	240	287	287	287	1	0.38
TiO2	Genotoxicity	Hep G2	≤24 h	1	50	50	50	192.5	192. 5	192. 5	1	30.79
rGO	Cytotoxicity	Hep G2	≤24 h	2	40	40	40	36.4	36.4	36.4	2	12.63

Table S14. Coefficients of the multiple linear regression relating human *in vitro* BMD_{10} (μg/mL) to nanoparticle type, assay category, cell type, exposure duration and particle size.

term	estimate	std.error	statistic	p.value	conf.low	conf.high
(Intercept)	-0.886	1.151	-0.77	0.444	-3.191	1.419
NPGO	0.826	0.63	1.31	0.195	-0.436	2.087
NPrGO	0.501	0.63	0.796	0.429	-0.76	1.763
NPSiO2	0.668	0.408	1.638	0.107	-0.149	1.485
NPTiO2	-0.02	0.39	-0.052	0.959	-0.801	0.76
general_experiment_typeCytokine	0.614	0.577	1.064	0.292	-0.541	1.769
general_experiment_typeCytotoxicity	-0.025	0.217	-0.116	0.908	-0.459	0.409
general_experiment_typeGenotoxicity	0.559	0.514	1.088	0.281	-0.47	1.587
Cell_typeCHANG	2.612	0.872	2.994	0.004	0.865	4.358
Cell_typeHepG2	0.753	0.691	1.09	0.28	-0.63	2.137
Cell_typeL-02	1.332	0.783	1.701	0.094	-0.236	2.899
Cell_typePHH	-0.39	0.95	-0.411	0.683	-2.292	1.512
exposure_time_num	0.016	0.006	2.805	0.007	0.005	0.028
log10_size	0.214	0.346	0.62	0.538	-0.478	0.906

Table S15. Variance decomposition of $\mathrm{BMD}_{10}^{\mathrm{IVIVE}}$ uncertainty for mouse in vitro experiments (n = 38). For each experiment, the $\log_{10}$-scale variance of the Monte Carlo BMD_{10} and DE_liver samples was computed separately and expressed as a percentage of their sum. Results are summarized as median [min–max] and grouped by assay category, nanoparticle core material, and overall. Cytokine endpoints show 100% DE_liver contribution because LOEC-based point estimates carry no posterior spread.

Grouping	Category	n	bmd_col	de_col
By general experiment type	Biochemical	2	67.5 [53.9-81.1]	32.5 [18.9-46.1]
By general experiment type	Cytokine	20	0 [0-0]	100 [100-100]
By general experiment type	Cytotoxicity	16	80.1 [5.3-98.4]	19.9 [1.6-94.7]
By NP	GO	2	80.1 [78.3-81.8]	19.9 [18.2-21.7]
By NP	Silica	13	5.3 [0-93.7]	94.7 [6.3-100]
By NP	TiO2	23	0 [0-98.4]	100 [1.6-100]
Overall	All	38	0 [0-98.4]	100 [1.6-100]

Table S16. Variance decomposition of $\mathrm{BMD}_{10}^{\mathrm{IVIVE}}$ uncertainty for human in vitro experiments (n = 76).

Grouping	Category	n	bmd_col	de_col
By general experiment type	Biochemical	26	50.2 [2-95.2]	49.8 [4.8-98]
By general experiment type	Cytokine	3	0 [0-0]	100 [100-100]
By general experiment type	Cytotoxicity	44	83 [2.7-99.5]	17 [0.5-97.3]
By general experiment type	Genotoxicity	3	29.5 [26.6-85.8]	70.5 [14.2-73.4]
By NP	Au	13	38.1 [0-93.4]	61.9 [6.6-100]
By NP	GO	2	80.8 [78.1-83.5]	19.2 [16.5-21.9]
By NP	Graphene quantum dots (GQD)	6	90.6 [49.1-97.6]	9.4 [2.4-50.9]
By NP	Silica	34	56.5 [2.7-99.5]	43.5 [0.5-97.3]
By NP	TiO2	19	89.1 [0-99]	10.9 [1-100]
By NP	rGO	2	51.1 [45.5-56.6]	48.9 [43.4-54.5]
Overall	All	76	58.8 [0-99.5]	41.2 [0.5-100]

Table S17. Uncertainty factors used to convert dose descriptors into $\mathrm{BMD}_{10}^{\mathrm{eq}}$. Abbreviations: EC_{25} and EC_{50}, effective concentration producing a 25% to 50% response of the maximal; LOEC, lowest observed effect concentration; LC_{10}/ EC_{10}/ IC_{10}, concentration producing a 10% lethal, effect, or inhibitory response; NOEC, no observed effect concentration; HONEC, highest observed no-effect concentration, the highest tested concentration at which no effect is observed.

**Dose descriptor**	**Uncertainty factor**	**Reference**
EC_{25} – EC_{50}	10	[1, 2]
LOEC	2	[3]
LC_{10}, EC_{10}, IC_{10}	1	[4, 5]
HONEC, NOEC	1	Equal to NOEC

Table S18. Mouse *in vivo* toxicity dataset with injection route.

ID	Material	Particle primary diameter [nm]	Endpoint type	Endpoint value (mg/kg)	Experiment type	BMD_{10}^{eq}	Reference
1	GO	500	LOEC-	16	Liver inflammatory (CD68)	8	[6]
2	GO	400	LOEC-	5	Liver inflammatory (ALT)	2.5	[7]
3	Silica	20	LOEC-	10	Liver inflammatory (mononuclear inflammatory)	5	[8]
4	Silica	80	LOEC-	10	Liver inflammatory (mononuclear inflammatory)	5	[8]
5	Silica	110	LOEC	240	Liver inflammatory (SOD)	120	[9]
6	Silica	110	LOEC	800	Mortality	400	[10]
7	Silica	64.43	LOEC	177.5	Liver inflammatory (CD68)	88.75	[11]
8	Silica	64.43	LD50	262.45	Mortality	26.245	[11]
9	Silica	50	LOEC	200	Mortality	100	[12]
10	Silica	50	LOEC	300	Mortality	150	[12]
11	Silica	200	HONEC	30	Mortality	30	[12]
12	Silica	200	HONEC	30	Mortality	30	[12]
13	TiO_2	42.30	LOEC	1387	Mortality	693.5	[13]
14	GO	20	HONEC	20	Liver inflammatory (ALT, AST, ALP)	20	[14]
15	Silica	70	LD50	45	Mortality	4.5	[15]
16	Silica	300	HONEC	100	Mortality	100	[15]
17	Silica	1000	HONEC	100	Mortality	100	[15]
18	Silica	100	LOEC	100	Mortality	50	[15]
19	Silica	110	LOEC	1280	Mortality	640	[16]
20	Silica	110	LOEC	500	Liver inflammatory (ALT, AST)	250	[16]
21	GO	54.9	NOEC	25	Reproductive toxicity	25	[17]
22	GO	237.9	HONEC	25	Reproductive toxicity	25	[17]
23	Silica	148.18	HONEC	300	Liver inflammatory (ALT, AST)	300	[18]

24	GO	23	HONEC	20	Mortality	20	[19]
25	GO	650	HONEC	20	Mortality	20	[20]
26	TiO_2	35	LOEC	64	Fetal growth	32	[21]
27	TiO_2	40	NOEC	1	Platelet activation	1	[22]

Table S19. Quantitative agreement between mouse *in vitro* $\mathrm{BMD}_{10}^{\mathrm{IVIVE}}$ and *in vivo* $\mathrm{BMD}_{10}^{\mathrm{eq}}$ by nanoparticle type, GM fold-difference: ratio of *in vitro* to *in vivo* GM doses. Within 10-fold: percentage of $\mathrm{BMD}_{10}^{\mathrm{IVIVE}}$ estimates falling within a factor of 10 of the *in vivo* $\mathrm{BMD}_{10}^{\mathrm{eq}}$ range.

Nanoparticle	$n_{in\ vitro}$	$n_{in\ vivo}$	GM fold-difference	Within 10-fold (%)
GO	2	2	1.34	100
SiO_2	13	13	0.119	100
TiO_2	23	20	0.128	87

Code & Results availability. Analysis code is available at: https://github.com/jimengwu/In-vitro-in-vivo-extrapolation-for-liver-toxicity.git.

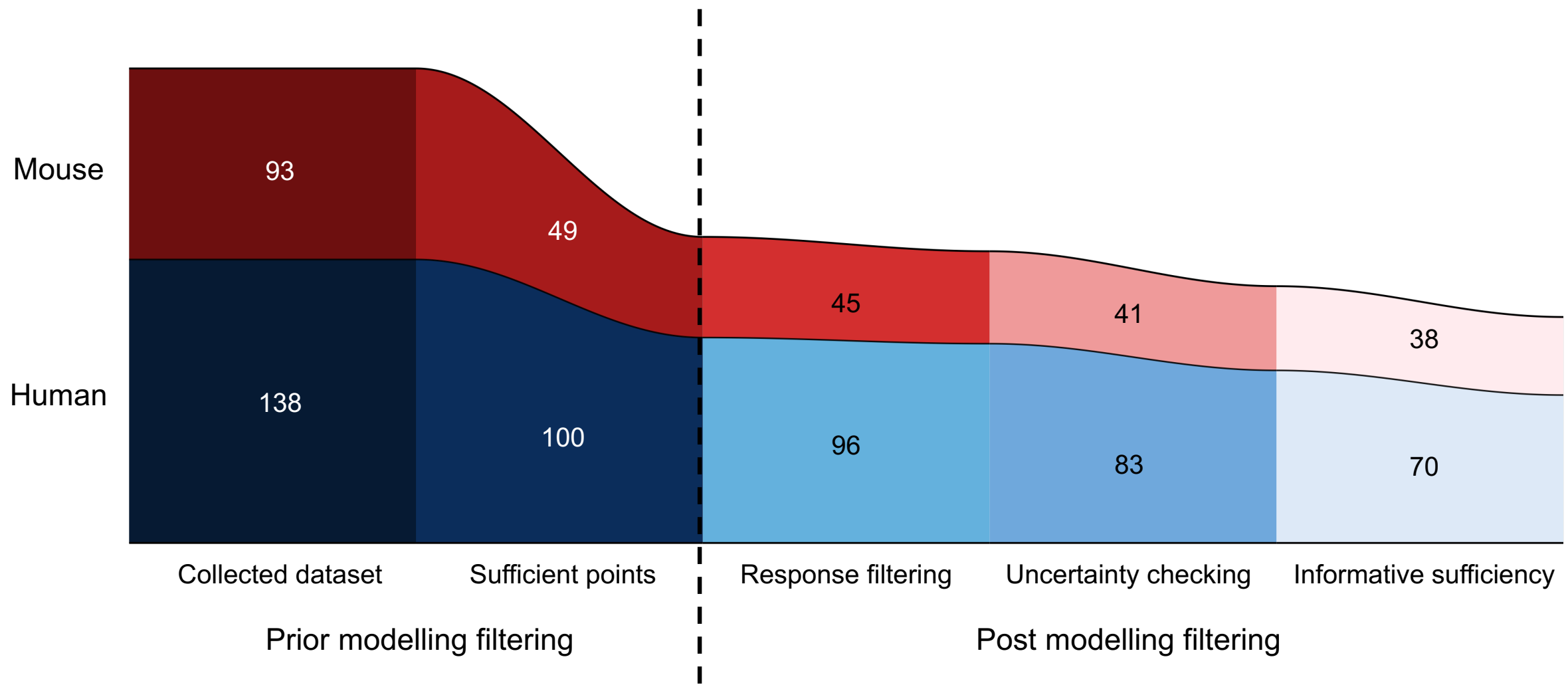

Figure S1. *Screening and quality control workflow for the compiled in vitro toxicity datasets before and after the dose–response modeling phase. Numbers within each segment indicate the number of datasets retained after each stage, reported separately for mice (top) and human (bottom). Pre-modeling filtering (left of the dashed line) retains experiments with a sufficient number of valid dose levels (mouse: 93 → 49; human: 138 → 100). Post-modeling filtering (right of the dashed line) applies three successive criteria: response filtering removes experiments lacking enough response level larger than 10 % for a reliable benchmark dose estimation (mouse: 49 → 45; human: 100 → 96); uncertainty checking excludes experiments with high uncertainty in the estimated benchmark dose (mouse: 45 → 41; human: 96 → 83), and informative sufficiency removes experiments with inconsistent or non-informative response patterns (mouse: 41 → 38; human: 83 → 70).*

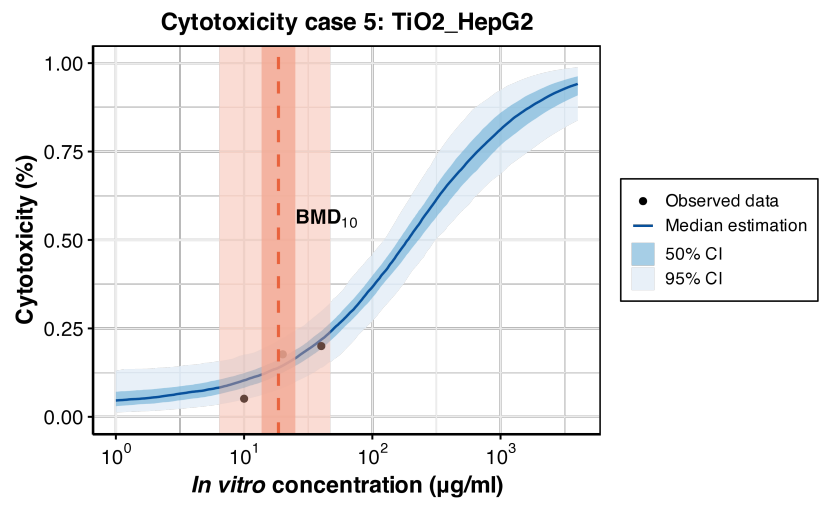
Cytotoxicity case 5: TiO2_HepG2
BMD10
Cytotoxicity (%)
In vitro concentration (μg/ml)
Observed data
Median estimation
50% CI
95% CI

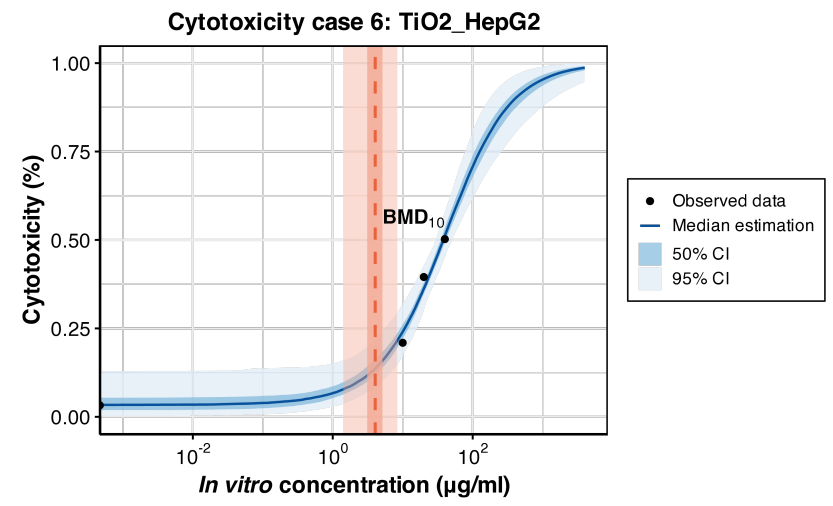
Cytotoxicity case 6: TiO2_HepG2
BMD10
Cytotoxicity (%)
In vitro concentration (μg/ml)
Observed data
Median estimation
50% CI
95% CI

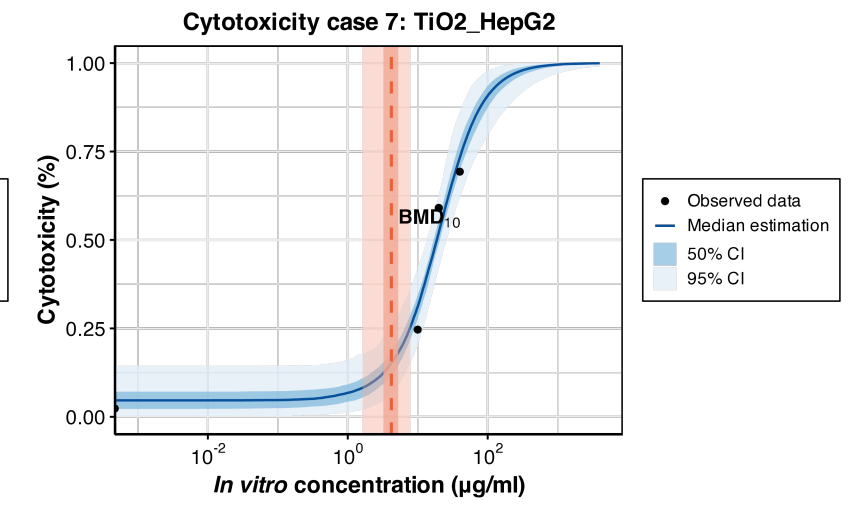
Cytotoxicity case 7: TiO2_HepG2
BMD10
Cytotoxicity (%)
In vitro concentration (μg/ml)
Observed data
Median estimation
50% CI
95% CI

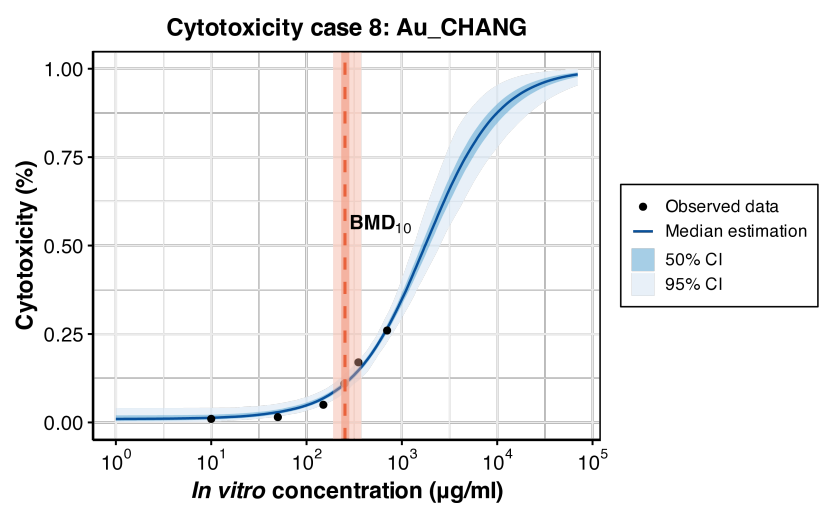
Cytotoxicity case 8: Au_CHANG
BMD10
Cytotoxicity (%)
In vitro concentration (μg/ml)
Observed data
Median estimation
50% CI
95% CI

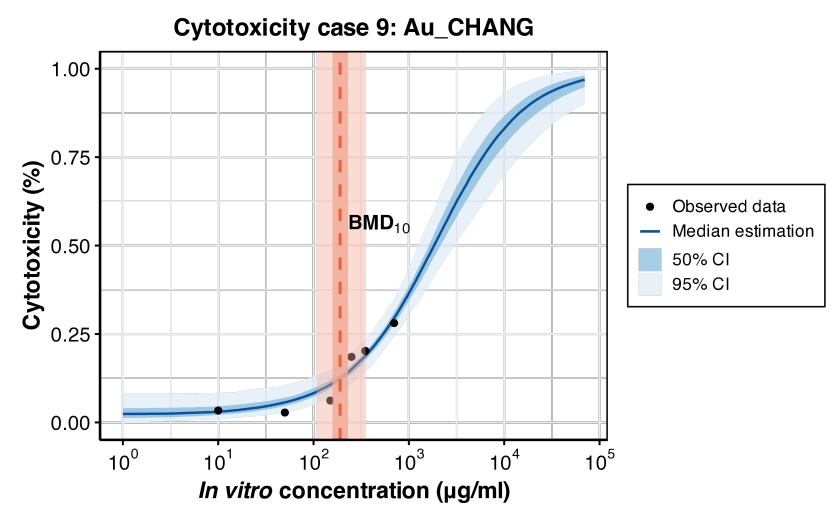
Cytotoxicity case 9: Au_CHANG
BMD10
Cytotoxicity (%)
In vitro concentration (μg/ml)
Observed data
Median estimation
50% CI
95% CI

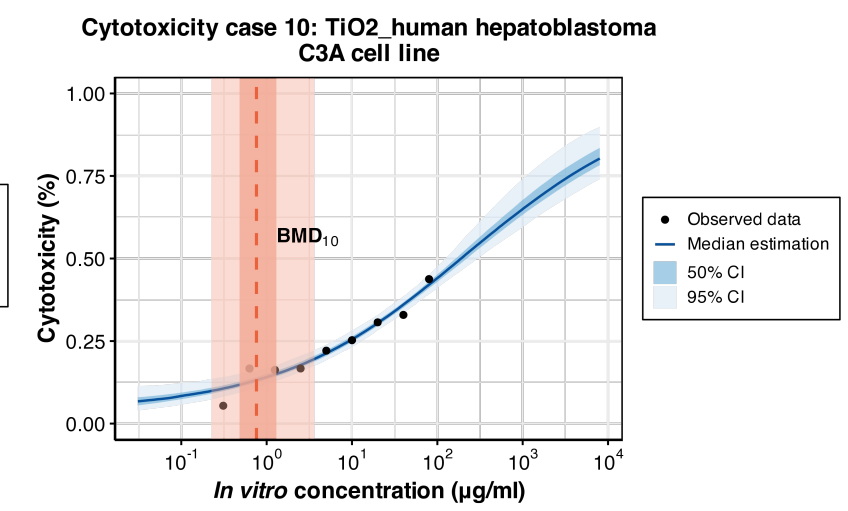
Cytotoxicity case 10: TiO2_human hepatoblastoma C3A cell line
BMD10
Cytotoxicity (%)
In vitro concentration (μg/ml)
Observed data
Median estimation
50% CI
95% CI

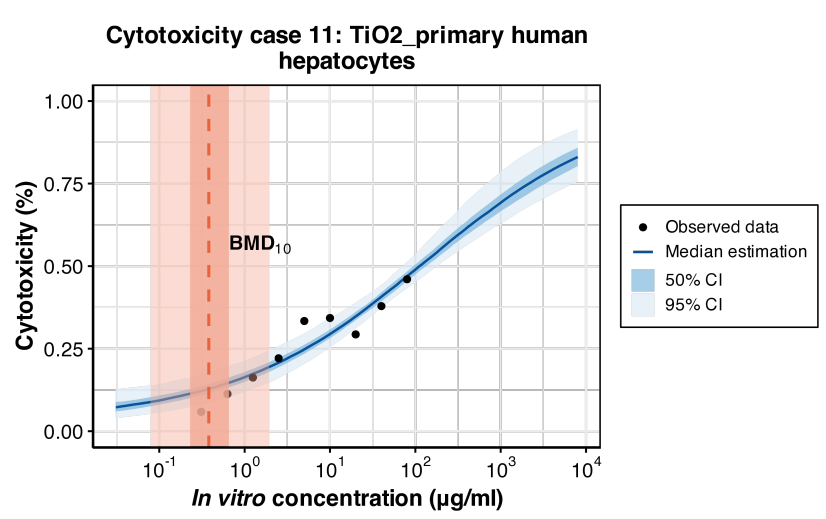
Cytotoxicity case 11: TiO2_primary human hepatocytes
BMD10
Cytotoxicity (%)
In vitro concentration (μg/ml)
Observed data
Median estimation
50% CI
95% CI

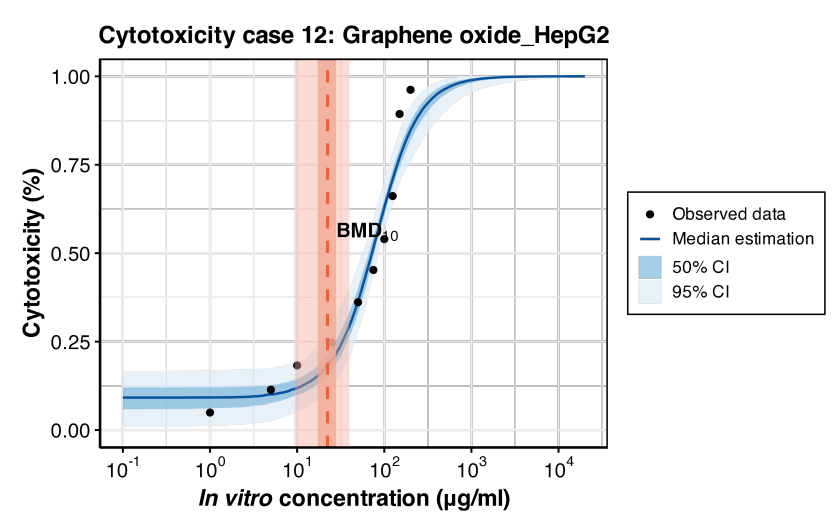
Cytotoxicity case 12: Graphene oxide_HepG2
BMD10
Cytotoxicity (%)
In vitro concentration (μg/ml)
Observed data
Median estimation
50% CI
95% CI

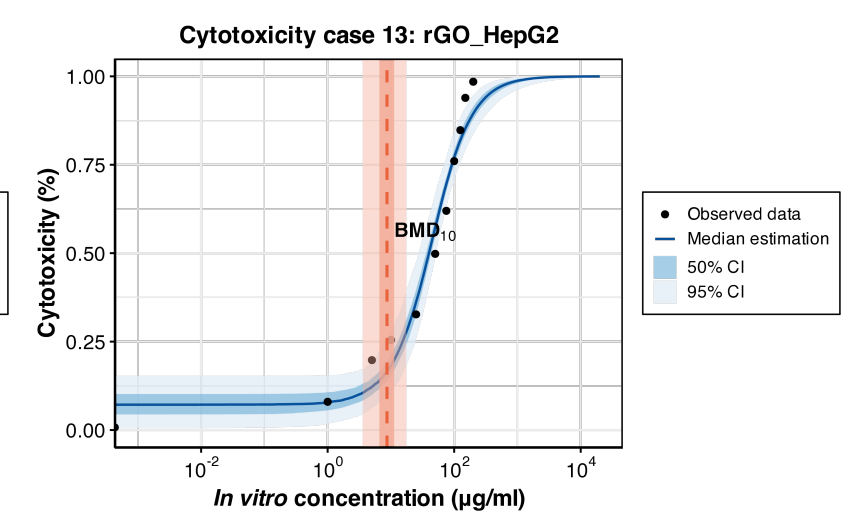
Cytotoxicity case 13: rGO_HepG2
BMD10
Cytotoxicity (%)
In vitro concentration (μg/ml)
Observed data
Median estimation
50% CI
95% CI

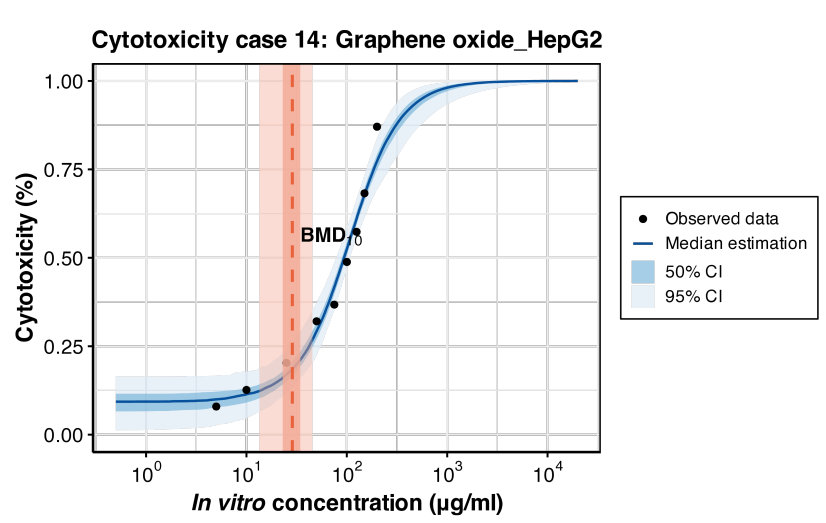
Cytotoxicity case 14: Graphene oxide_HepG2
BMD10
Cytotoxicity (%)
In vitro concentration (μg/ml)
Observed data
Median estimation
50% CI
95% CI

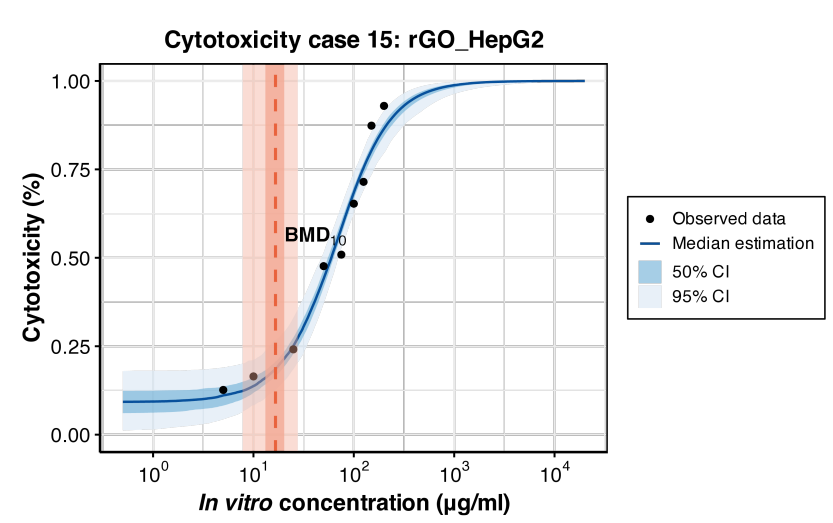
Cytotoxicity case 15: rGO_HepG2
BMD10
Cytotoxicity (%)
In vitro concentration (μg/ml)
Observed data
Median estimation
50% CI
95% CI

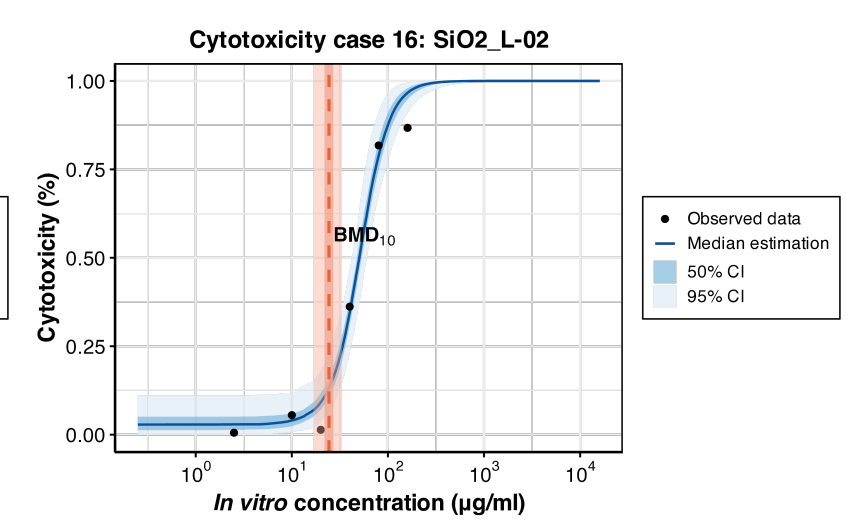
Cytotoxicity case 16: SiO2_L-02
BMD10
Cytotoxicity (%)
In vitro concentration (μg/ml)
Observed data
Median estimation
50% CI
95% CI

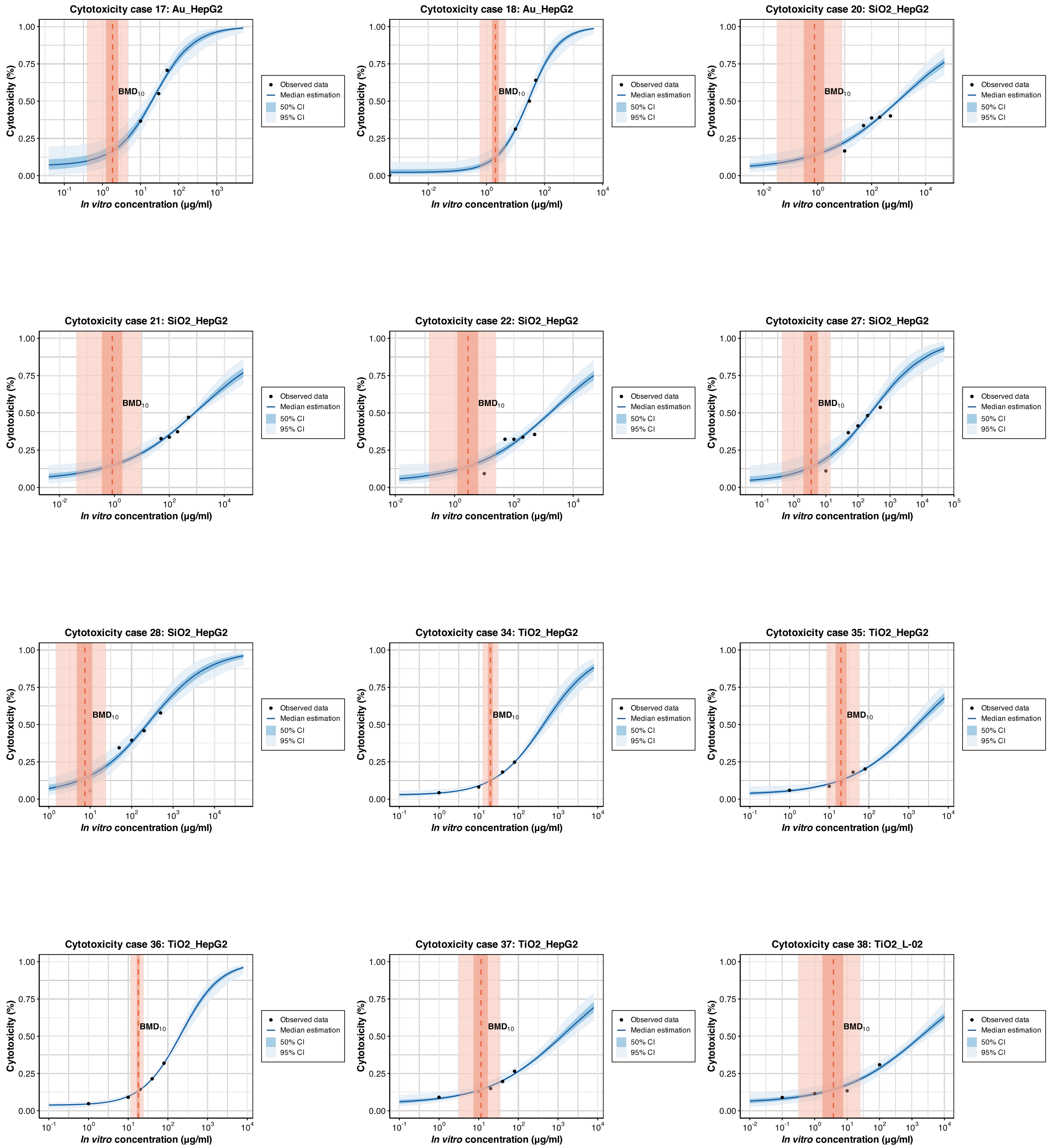
Cytotoxicity case 17: Au_HepG2
Cytotoxicity case 18: Au_HepG2
Cytotoxicity case 20: SiO2_HepG2
Cytotoxicity case 21: SiO2_HepG2
Cytotoxicity case 22: SiO2_HepG2
Cytotoxicity case 27: SiO2_HepG2
Cytotoxicity case 28: SiO2_HepG2
Cytotoxicity case 34: TiO2_HepG2
Cytotoxicity case 35: TiO2_HepG2
Cytotoxicity case 36: TiO2_HepG2
Cytotoxicity case 37: TiO2_HepG2
Cytotoxicity case 38: TiO2_L-02
BMD10
Cytotoxicity (%)
In vitro concentration (µg/ml)
Observed data
Median estimation
50% CI
95% CI

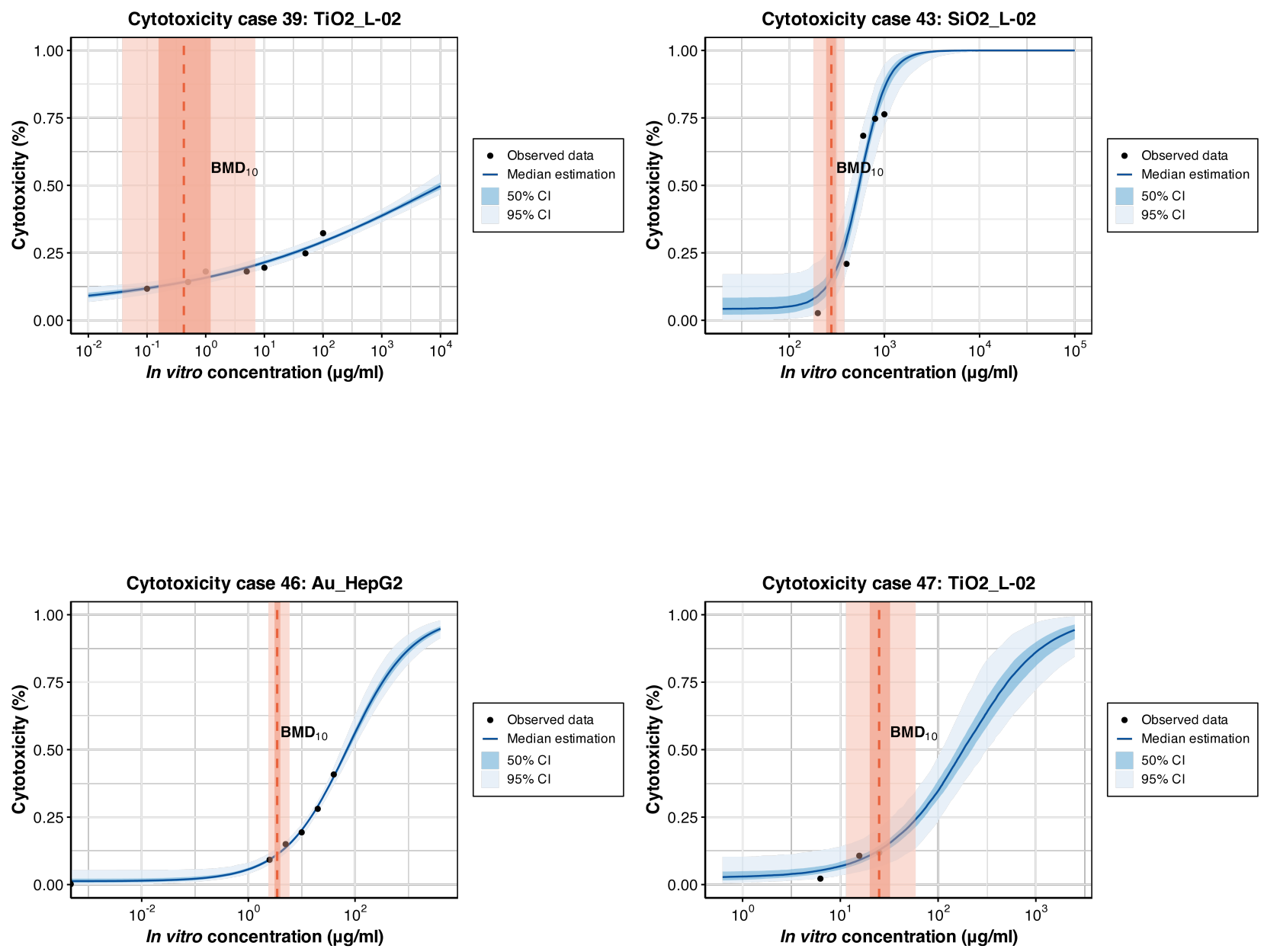

Figure S2. *Dose response curves for human cytotoxicity experiments retained for in vitro to in vivo extrapolation. Each panel shows the fitted three-parameter Hill model for a specific experiment, with the median estimate (solid line) and 50% and 95% credible intervals (shaded ribbons). Black dots represent observed data points. The vertical dashed line and shaded band indicate the median BMD_{10} estimate and its credible interval, respectively.*

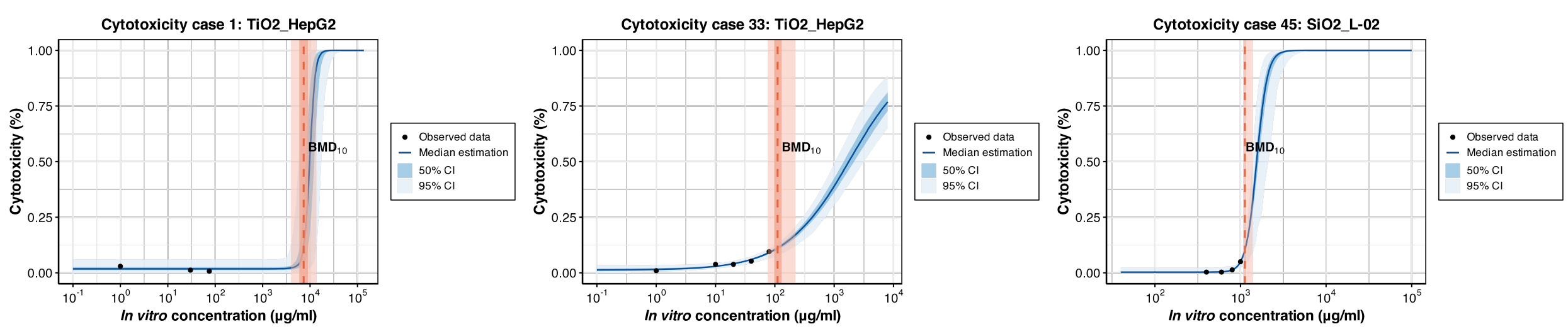

Figure S3. *Dose response curves for human cytotoxicity experiments excluded from further extrapolation due to insufficient response magnitude. These experiments showed less than 10% change from the negative control across all tested concentrations, precluding reliable BMD_{10} estimation. Formatting follows Figure S1.*

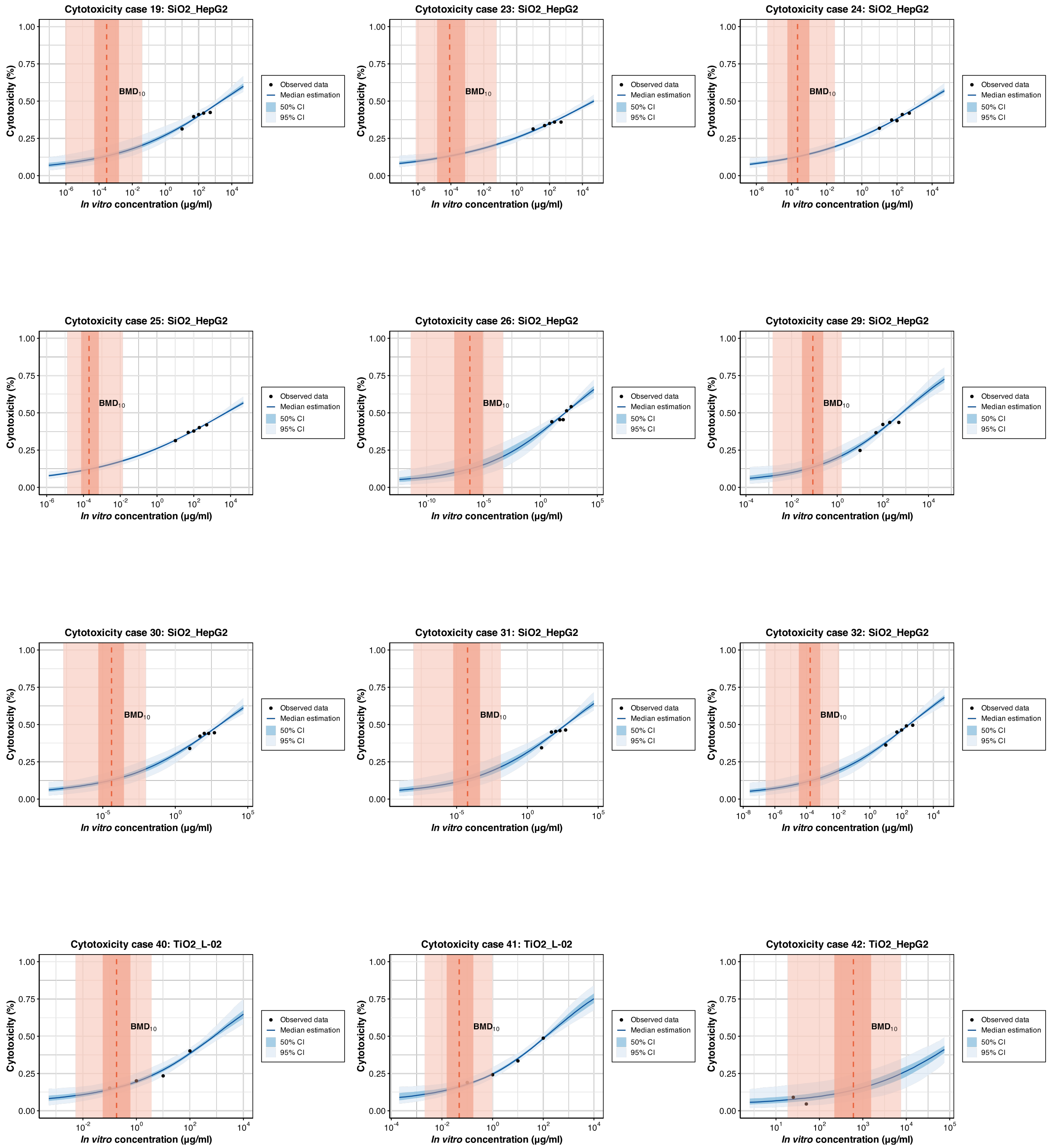

Figure S4. *Dose response curves for human cytotoxicity experiments excluded from further extrapolation due to excessive BMD_{10} uncertainty. These experiments yielded BMD_{10} credible intervals spanning more than three orders of magnitude, indicating insufficient constraint on the dose–response relationship. Formatting follows Figure S1.*

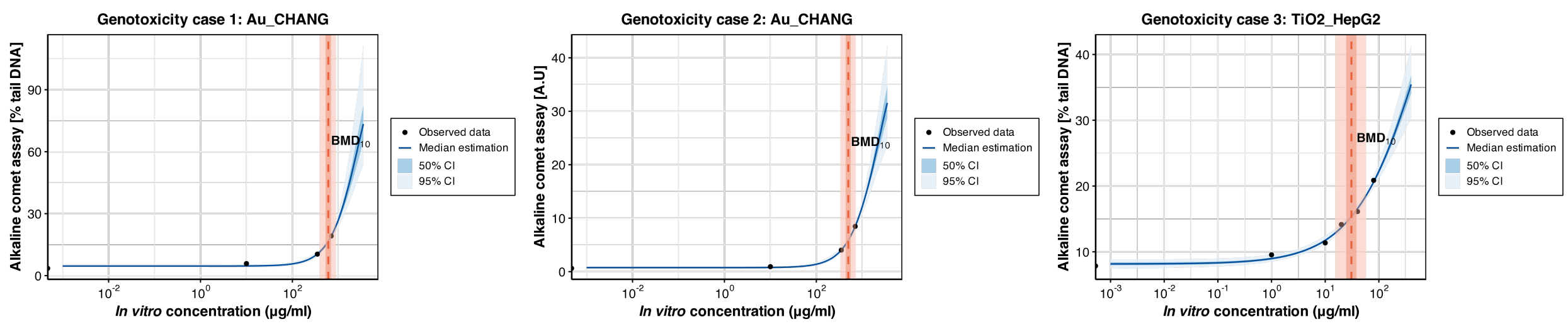

Figure S5. *Dose response curves for human genotoxicity experiments retained for in vitro to in vivo extrapolation. Formatting follows Figure S1.*

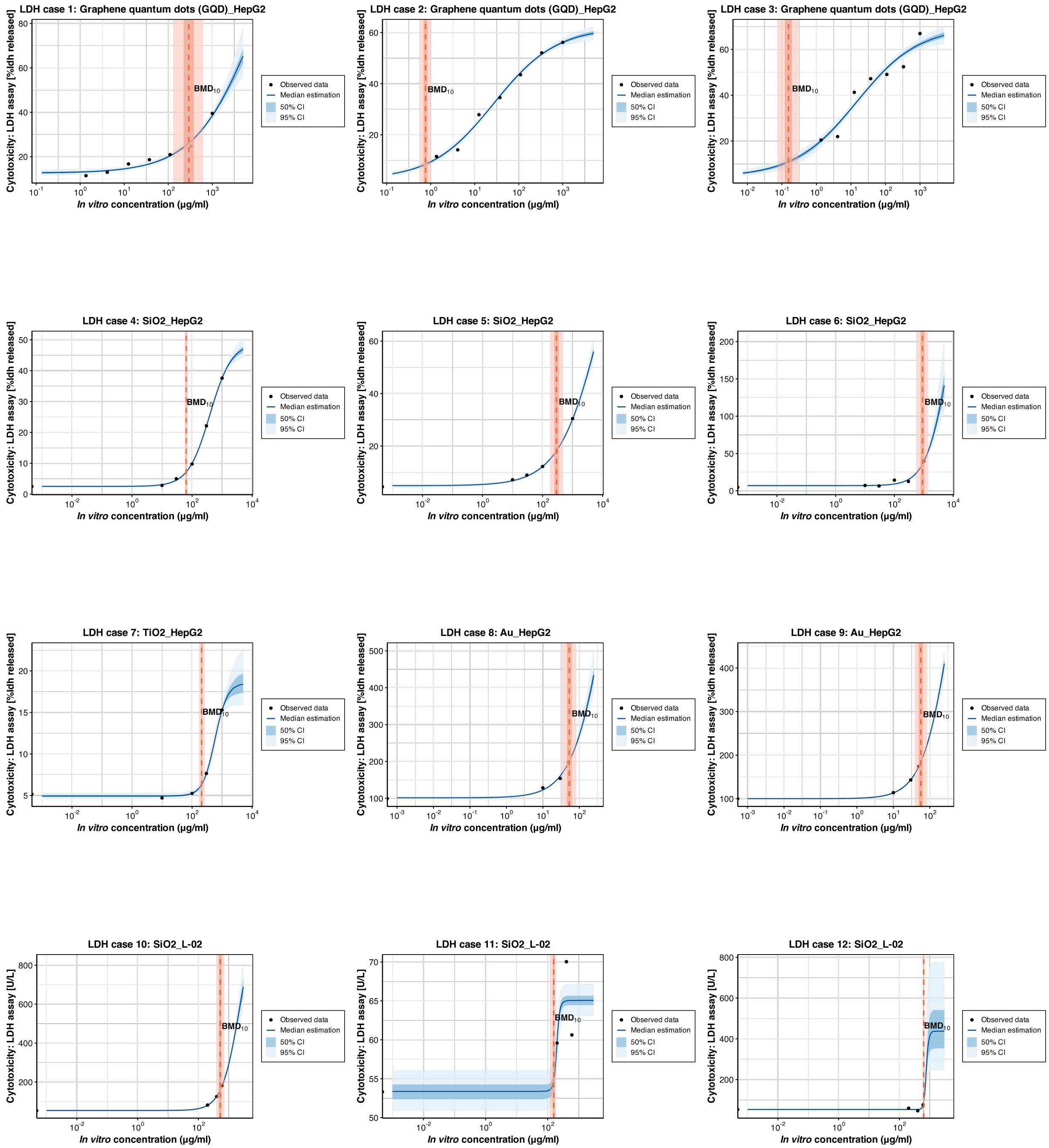

Figure S6. *Dose response curves for human LDH experiments retained for in vitro to in vivo extrapolation. Formatting follows Figure S1.*

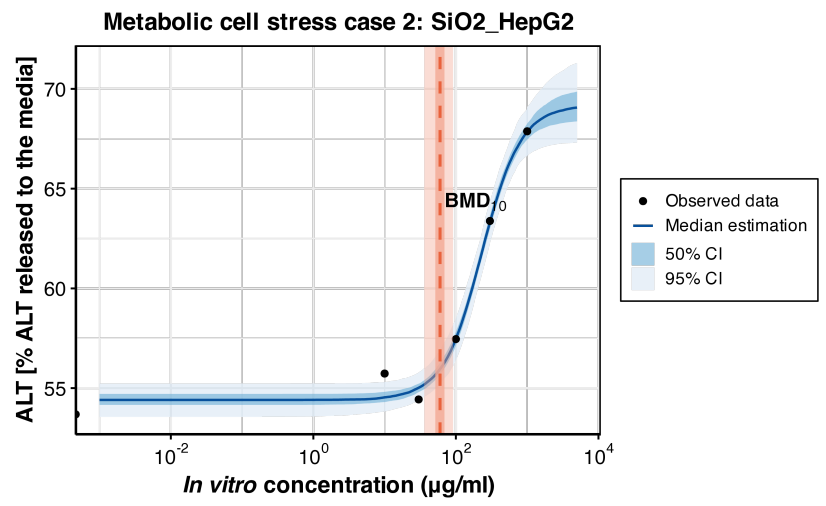
Metabolic cell stress case 2: SiO2_HepG2
ALT [% ALT released to the media]
BMD10
In vitro concentration (µg/ml)
Observed data
Median estimation
50% CI
95% CI

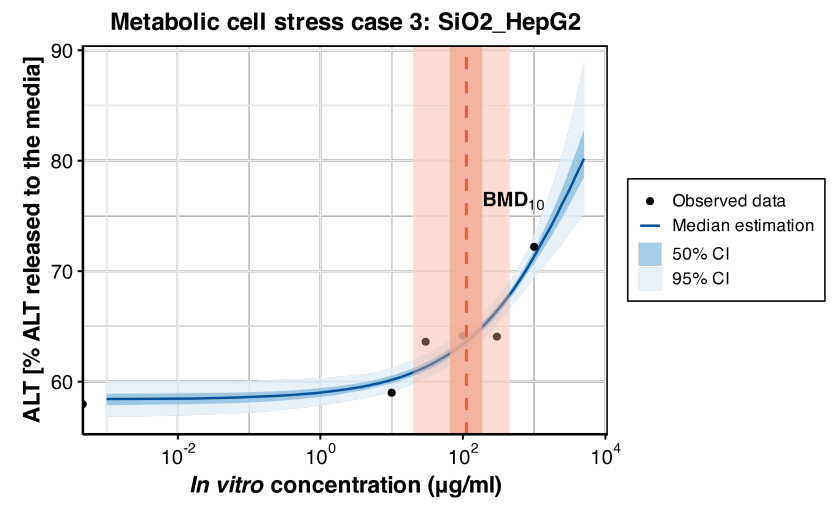
Metabolic cell stress case 3: SiO2_HepG2
ALT [% ALT released to the media]
BMD10
In vitro concentration (µg/ml)
Observed data
Median estimation
50% CI
95% CI

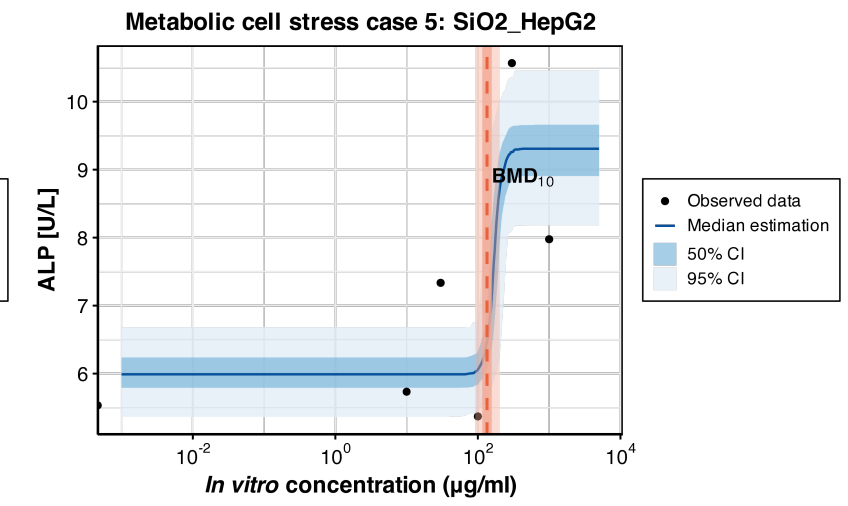
Metabolic cell stress case 5: SiO2_HepG2
ALP [U/L]
BMD10
In vitro concentration (µg/ml)
Observed data
Median estimation
50% CI
95% CI

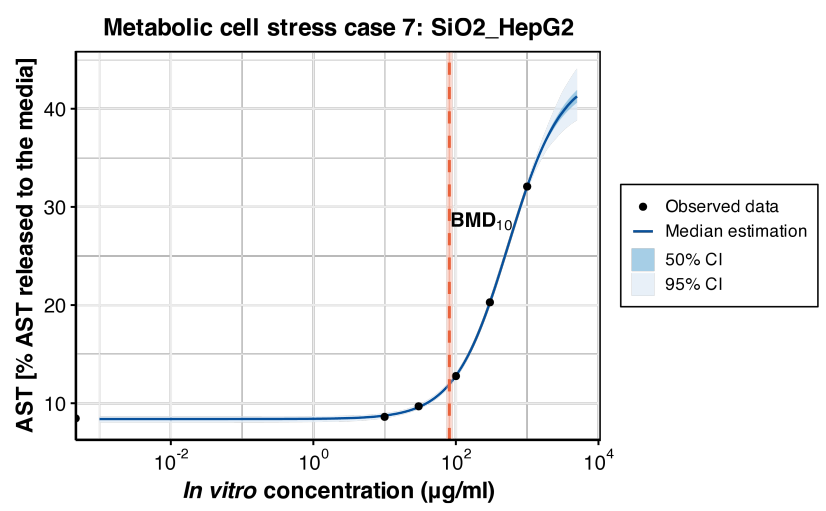
Metabolic cell stress case 7: SiO2_HepG2
AST [% AST released to the media]
BMD10
In vitro concentration (µg/ml)
Observed data
Median estimation
50% CI
95% CI

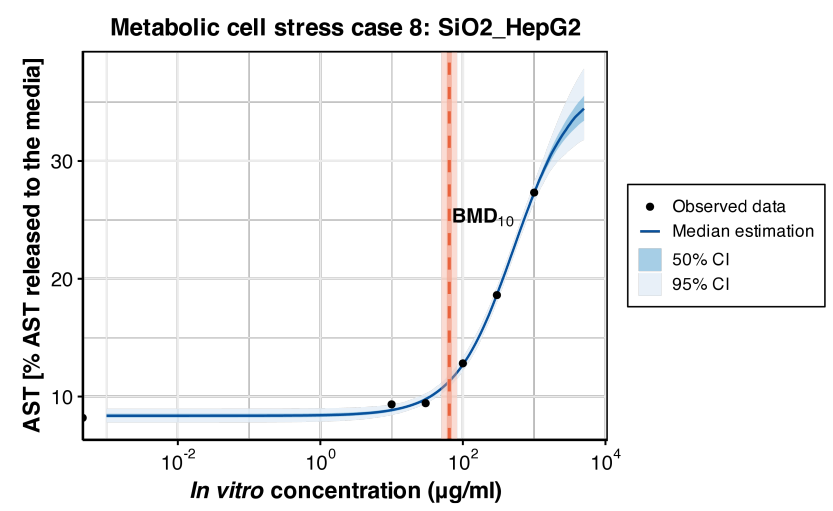
Metabolic cell stress case 8: SiO2_HepG2
AST [% AST released to the media]
BMD10
In vitro concentration (µg/ml)
Observed data
Median estimation
50% CI
95% CI

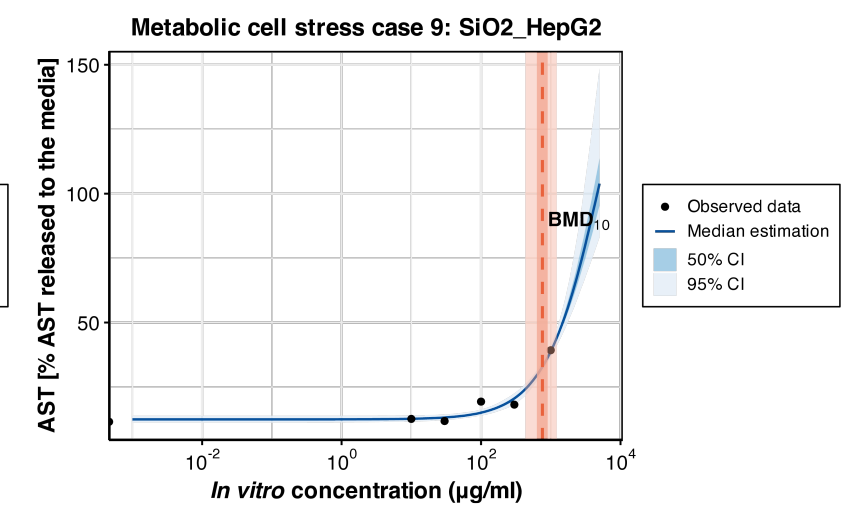
Metabolic cell stress case 9: SiO2_HepG2
AST [% AST released to the media]
BMD10
In vitro concentration (µg/ml)
Observed data
Median estimation
50% CI
95% CI

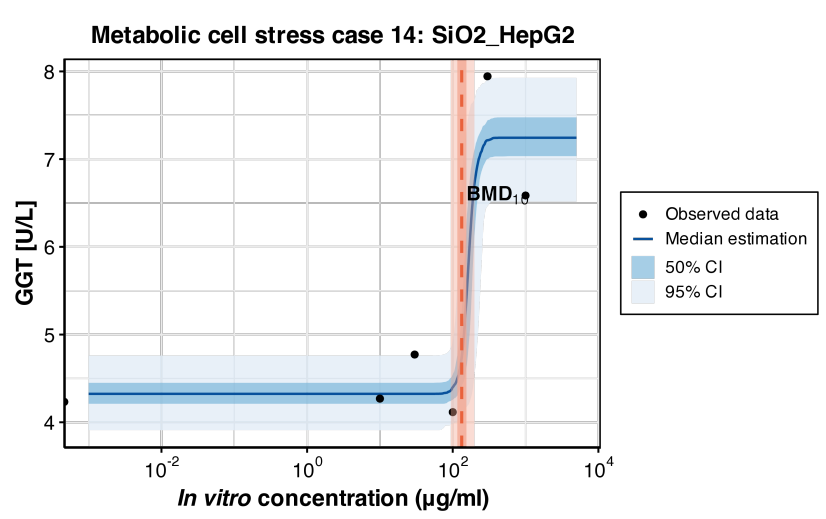
Metabolic cell stress case 14: SiO2_HepG2
GGT [U/L]
BMD10
In vitro concentration (µg/ml)
Observed data
Median estimation
50% CI
95% CI

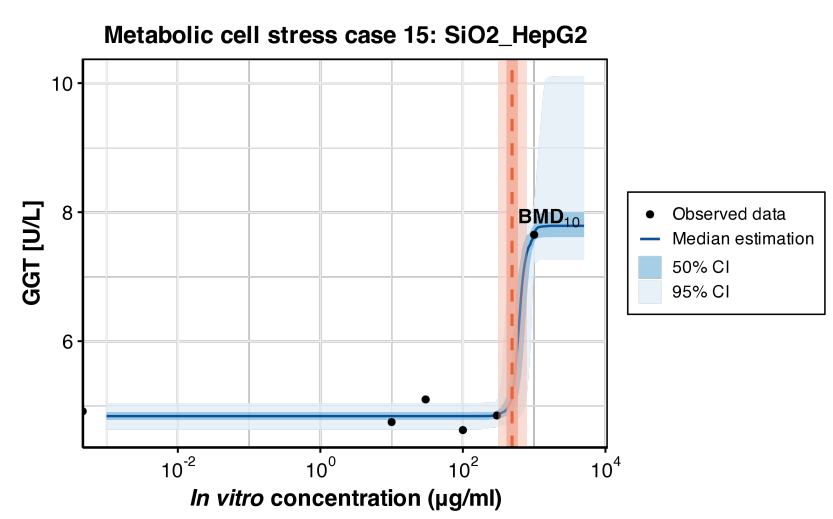
Metabolic cell stress case 15: SiO2_HepG2
GGT [U/L]
BMD10
In vitro concentration (µg/ml)
Observed data
Median estimation
50% CI
95% CI

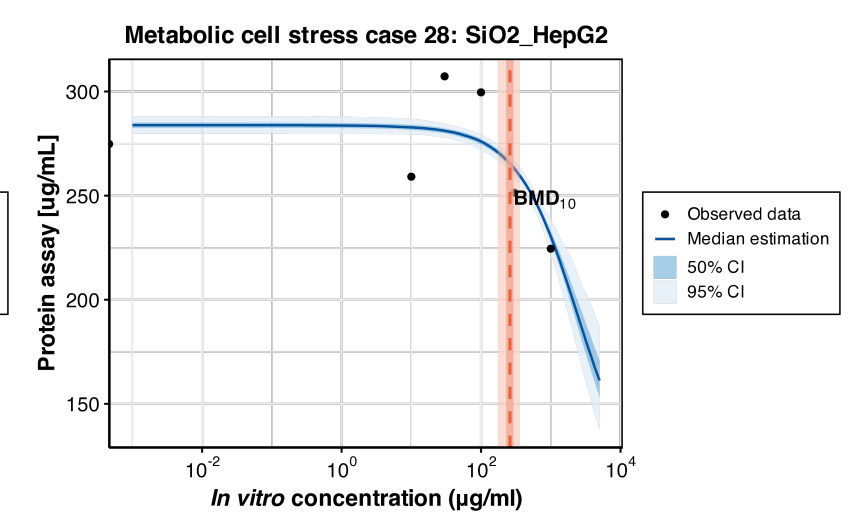
Metabolic cell stress case 28: SiO2_HepG2
Protein assay [ug/mL]
BMD10
In vitro concentration (µg/ml)
Observed data
Median estimation
50% CI
95% CI

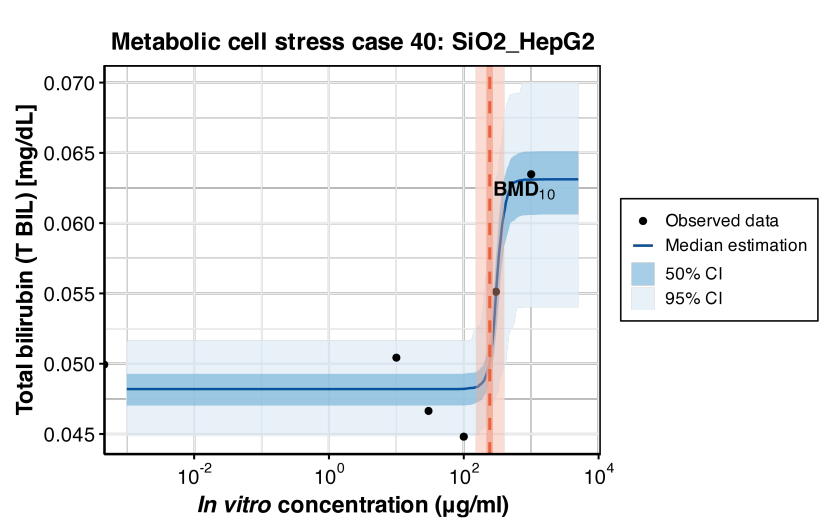
Metabolic cell stress case 40: SiO2_HepG2
Total bilirubin (T BIL) [mg/dL]
BMD10
In vitro concentration (µg/ml)
Observed data
Median estimation
50% CI
95% CI

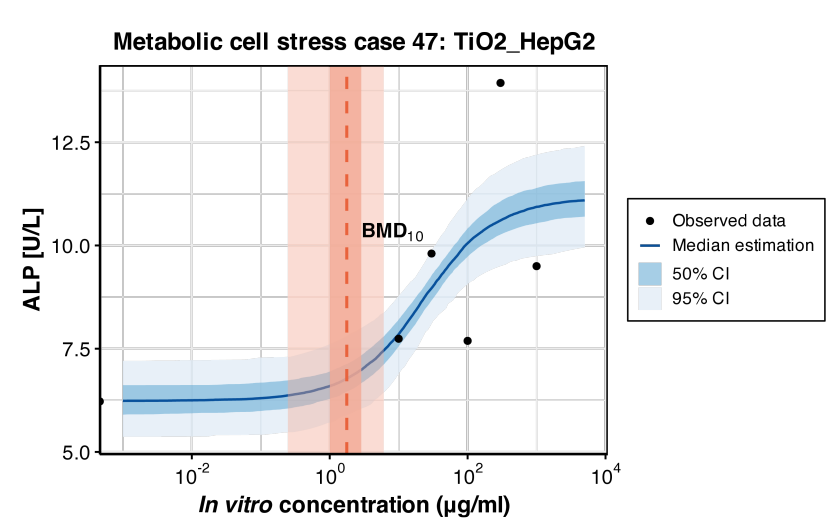
Metabolic cell stress case 47: TiO2_HepG2
ALP [U/L]
BMD10
In vitro concentration (µg/ml)
Observed data
Median estimation
50% CI
95% CI

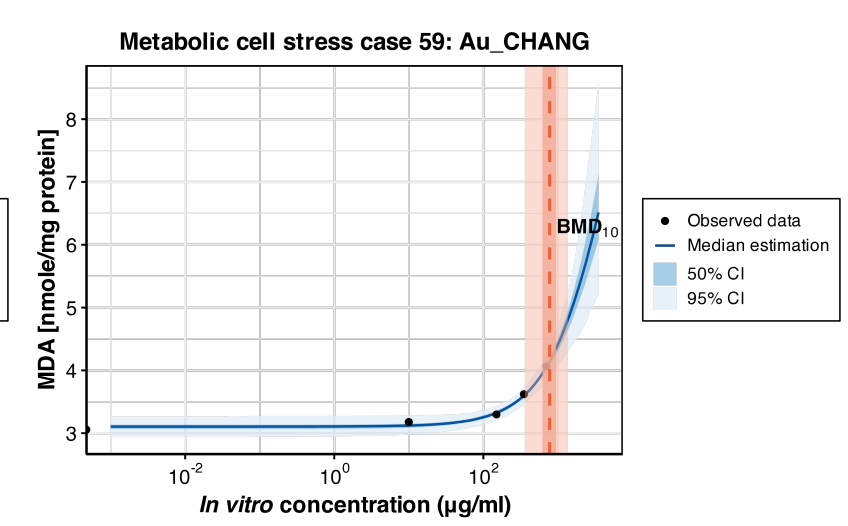
Metabolic cell stress case 59: Au_CHANG
MDA [nmole/mg protein]
BMD10
In vitro concentration (µg/ml)
Observed data
Median estimation
50% CI
95% CI

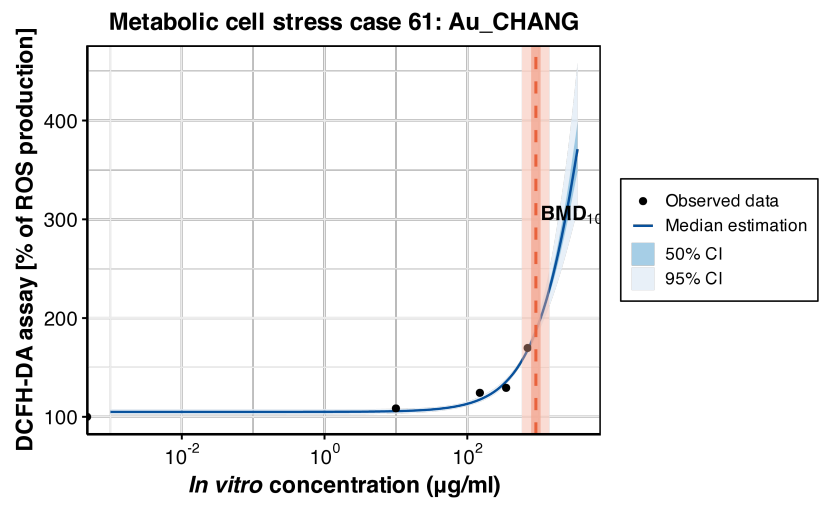
Metabolic cell stress case 61: Au_CHANG
DCFH-DA assay [% of ROS production]
In vitro concentration (µg/ml)
BMD10
Observed data
Median estimation
50% CI
95% CI

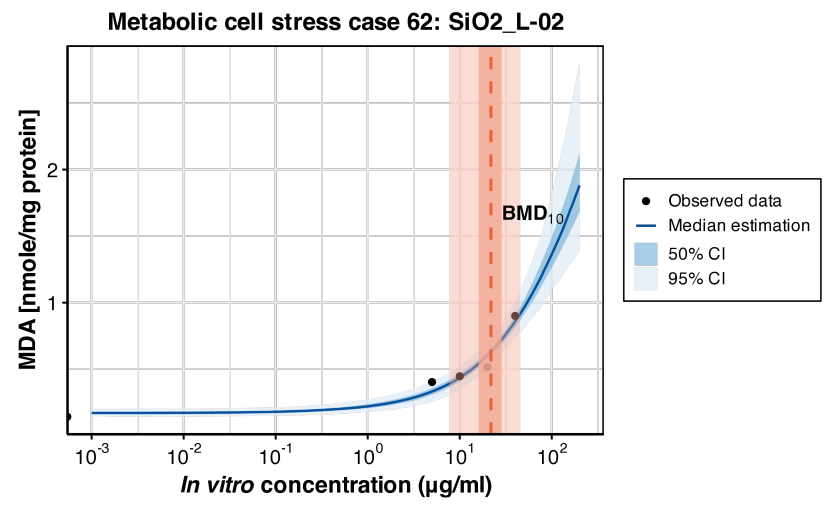
Metabolic cell stress case 62: SiO2_L-02
MDA [nmole/mg protein]
In vitro concentration (µg/ml)
BMD10
Observed data
Median estimation
50% CI
95% CI

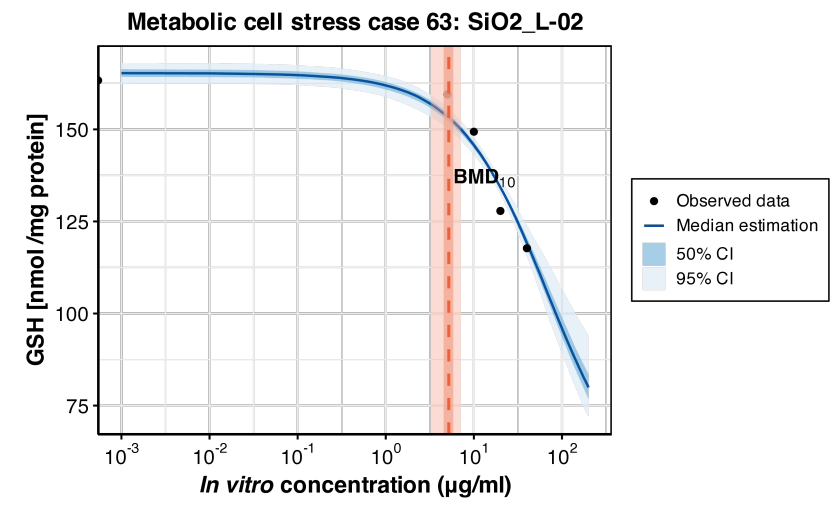
Metabolic cell stress case 63: SiO2_L-02
GSH [nmol /mg protein]
In vitro concentration (µg/ml)
BMD10
Observed data
Median estimation
50% CI
95% CI

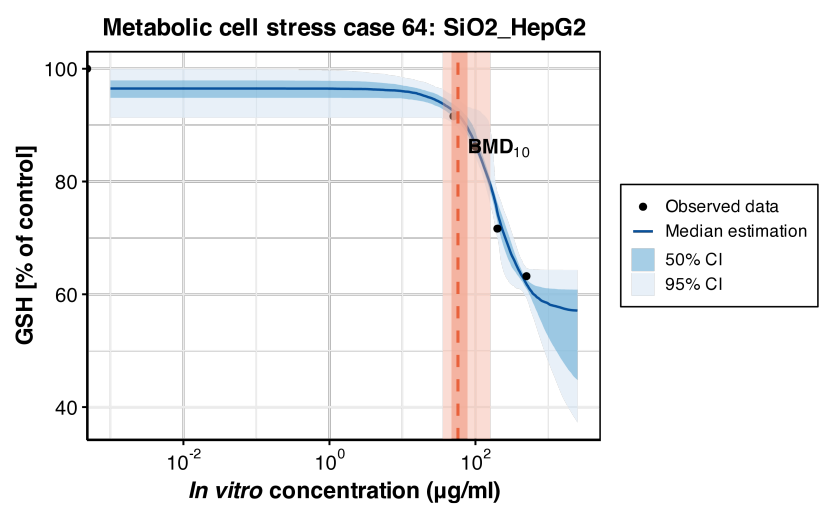
Metabolic cell stress case 64: SiO2_HepG2
GSH [% of control]
In vitro concentration (µg/ml)
BMD10
Observed data
Median estimation
50% CI
95% CI

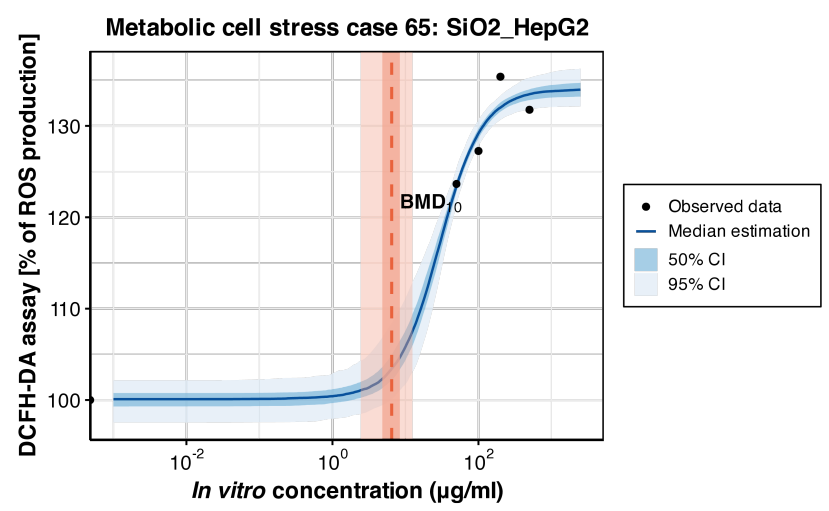
Metabolic cell stress case 65: SiO2_HepG2
DCFH-DA assay [% of ROS production]
In vitro concentration (µg/ml)
BMD10
Observed data
Median estimation
50% CI
95% CI

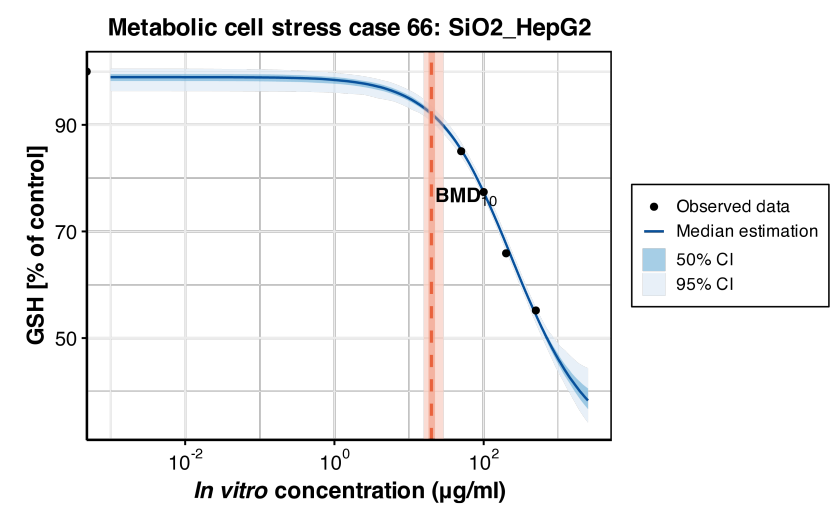
Metabolic cell stress case 66: SiO2_HepG2
GSH [% of control]
In vitro concentration (µg/ml)
BMD10
Observed data
Median estimation
50% CI
95% CI

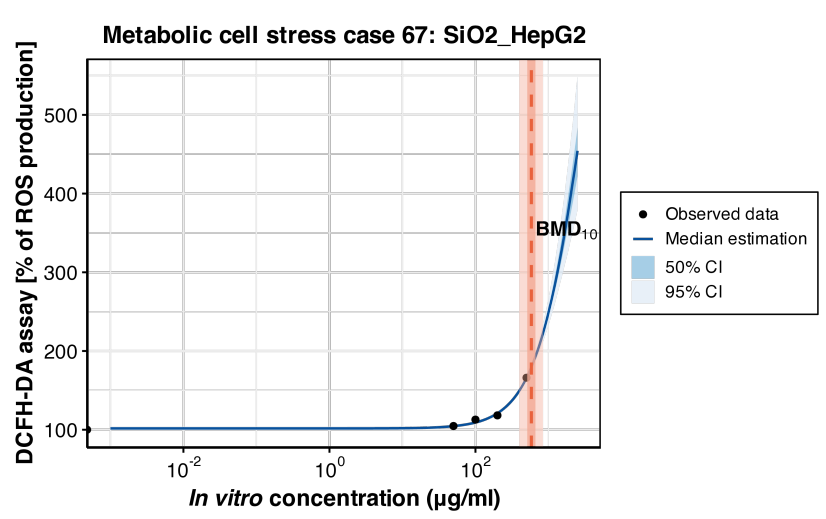
Metabolic cell stress case 67: SiO2_HepG2
DCFH-DA assay [% of ROS production]
In vitro concentration (µg/ml)
BMD10
Observed data
Median estimation
50% CI
95% CI

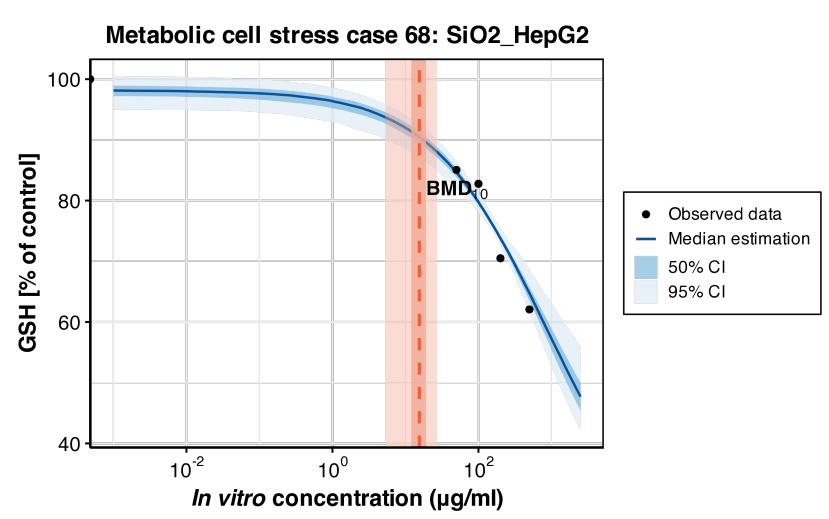
Metabolic cell stress case 68: SiO2_HepG2
GSH [% of control]
In vitro concentration (µg/ml)
BMD10
Observed data
Median estimation
50% CI
95% CI

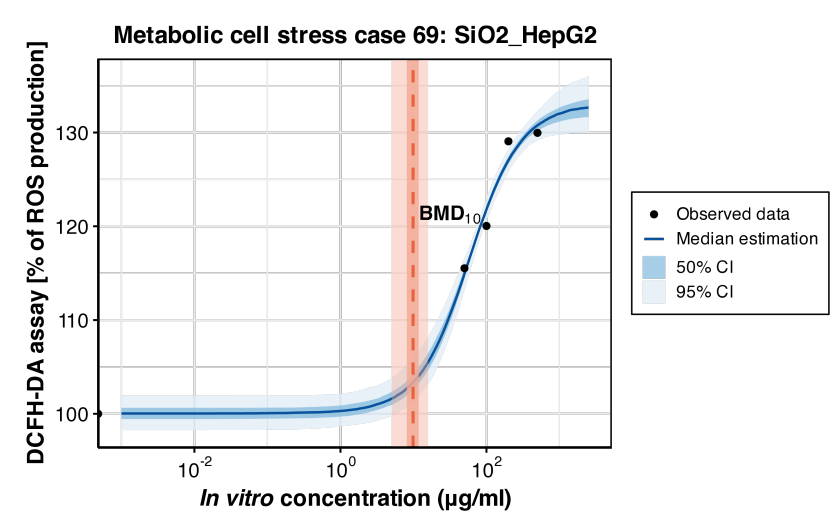
Metabolic cell stress case 69: SiO2_HepG2
DCFH-DA assay [% of ROS production]
In vitro concentration (µg/ml)
BMD10
Observed data
Median estimation
50% CI
95% CI

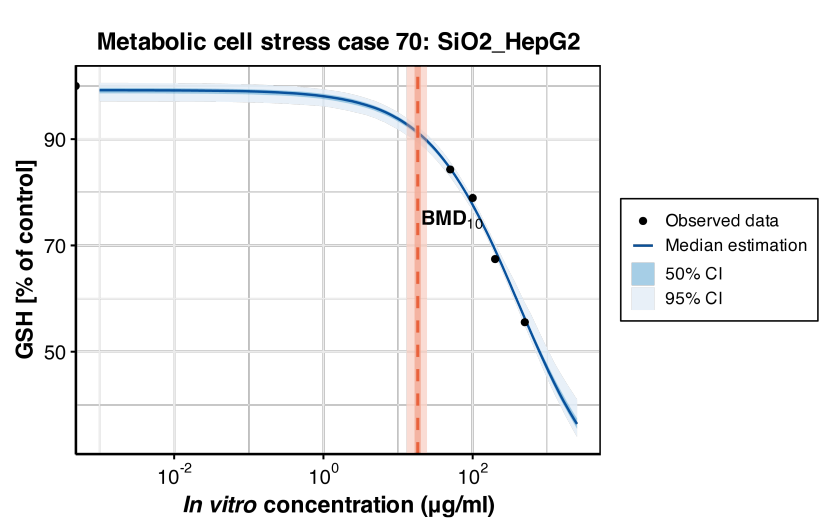
Metabolic cell stress case 70: SiO2_HepG2
GSH [% of control]
In vitro concentration (µg/ml)
BMD10
Observed data
Median estimation
50% CI
95% CI

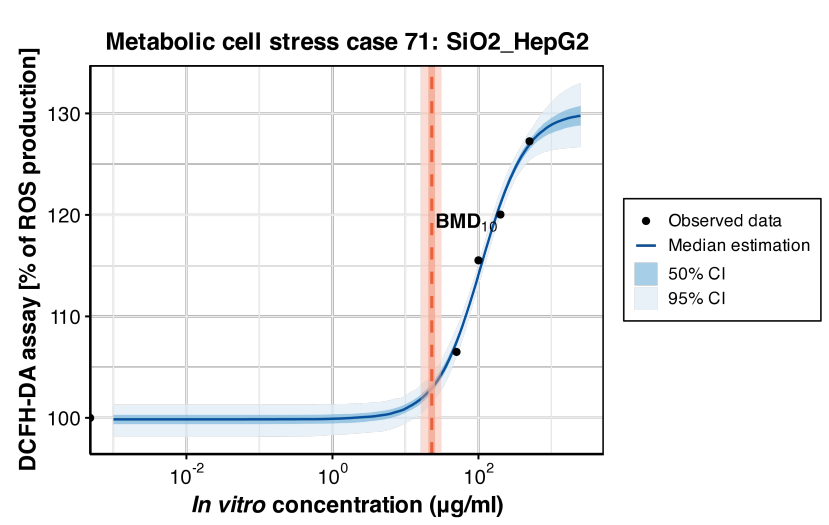
Metabolic cell stress case 71: SiO2_HepG2
DCFH-DA assay [% of ROS production]
In vitro concentration (µg/ml)
BMD10
Observed data
Median estimation
50% CI
95% CI

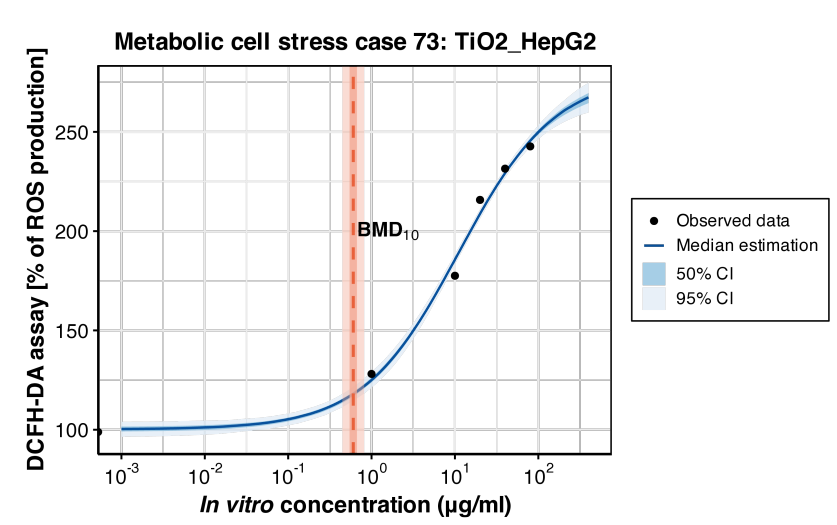
Metabolic cell stress case 73: TiO2_HepG2
DCFH-DA assay [% of ROS production]
In vitro concentration (µg/ml)
BMD10
Observed data
Median estimation
50% CI
95% CI

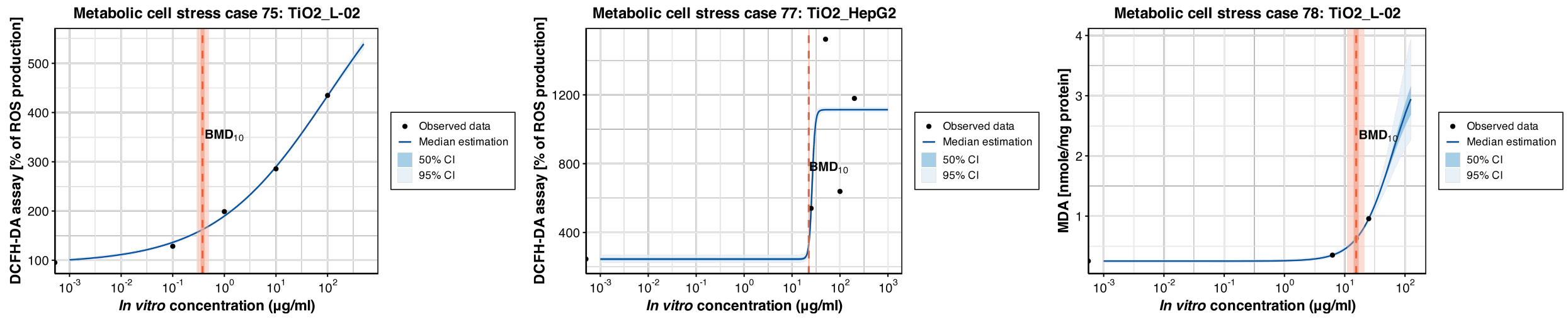

Figure S7. *Dose response curves for human metabolic cell stress experiments retained for in vitro to in vivo extrapolation. Formatting follows Figure S1.*

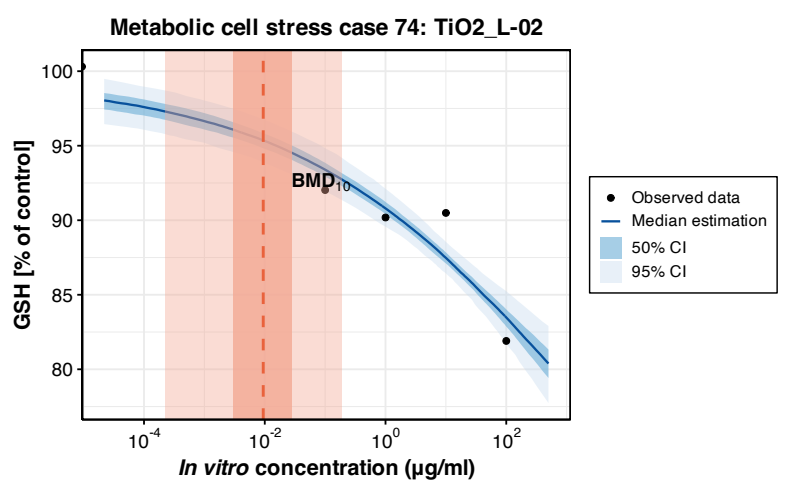

Figure S8. *Dose–response curves for human metabolic cell stress experiments excluded from further extrapolation due to excessive BMD_{10} uncertainty. These experiments yielded BMD_{10} credible intervals spanning more than three orders of magnitude, indicating insufficient constraint on the dose–response relationship. Formatting follows Figure S1.*

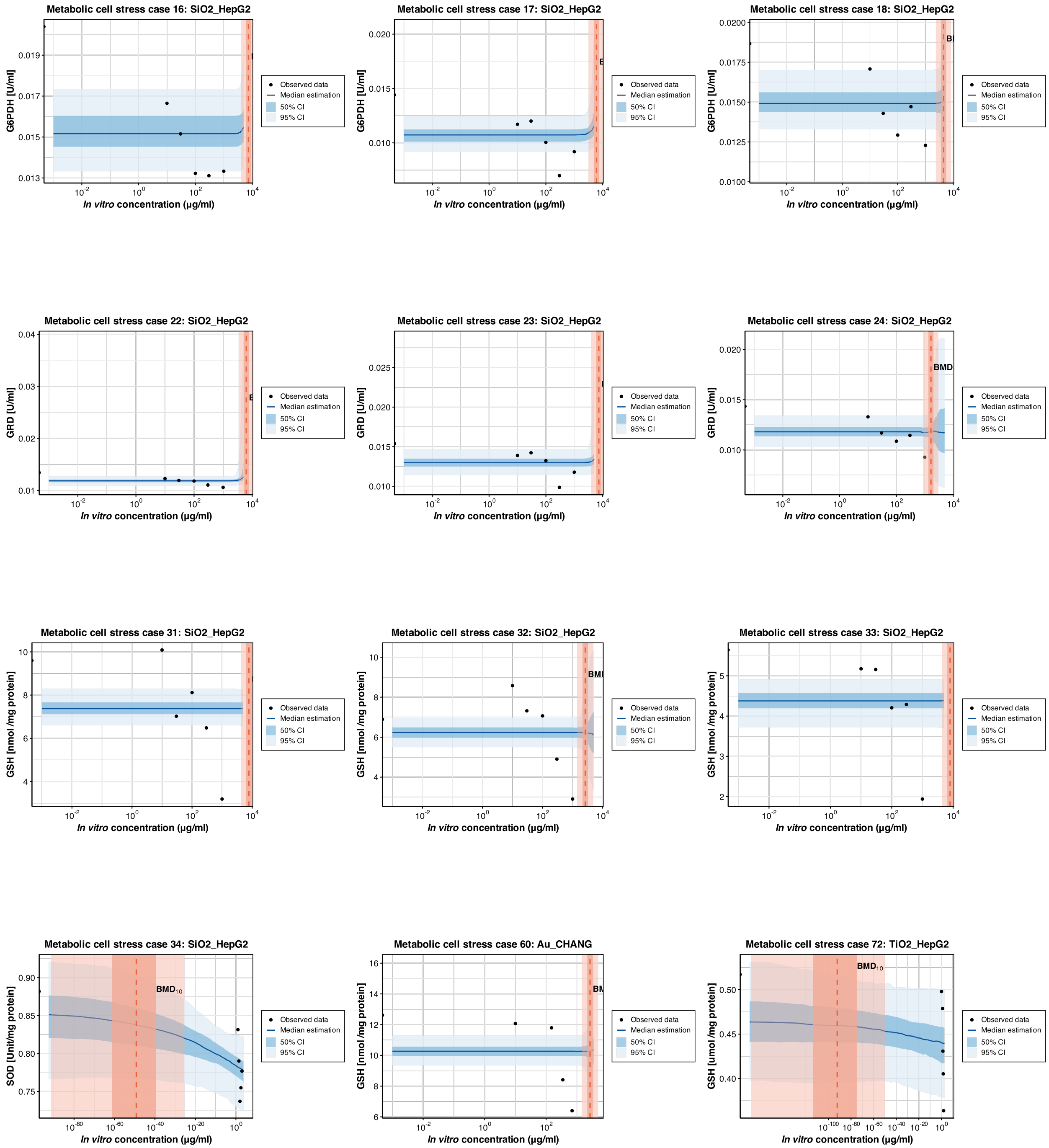

Figure S9. *Dose–response curves for human metabolic cell stress experiments excluded from further extrapolation due to insufficient informative content. These experiments exhibited inconsistent or non-informative response patterns that did not support reliable dose–response characterization despite passing initial data point and response magnitude criteria. Formatting follows Figure S1.*

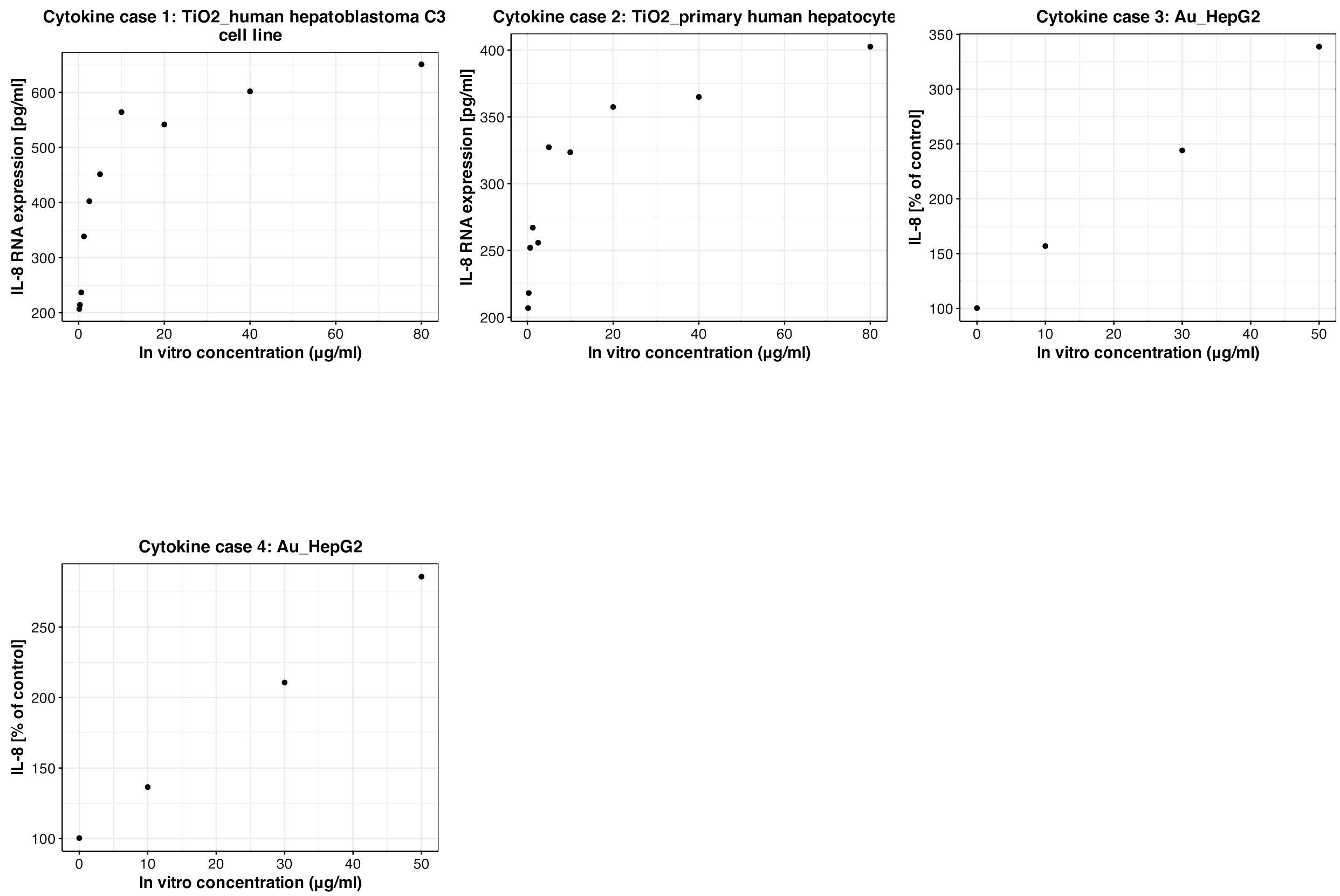

Figure S10. *Cytokine endpoint data for human in vitro experiments. Each panel shows measured cytokine responses (e.g., TNF-α, IL-6, IL-8, IL-1β) as a function of nanoparticle concentration. Classical dose response curve fitting was not applied to these endpoints due to their non-monotonic or threshold-triggered release profiles.*

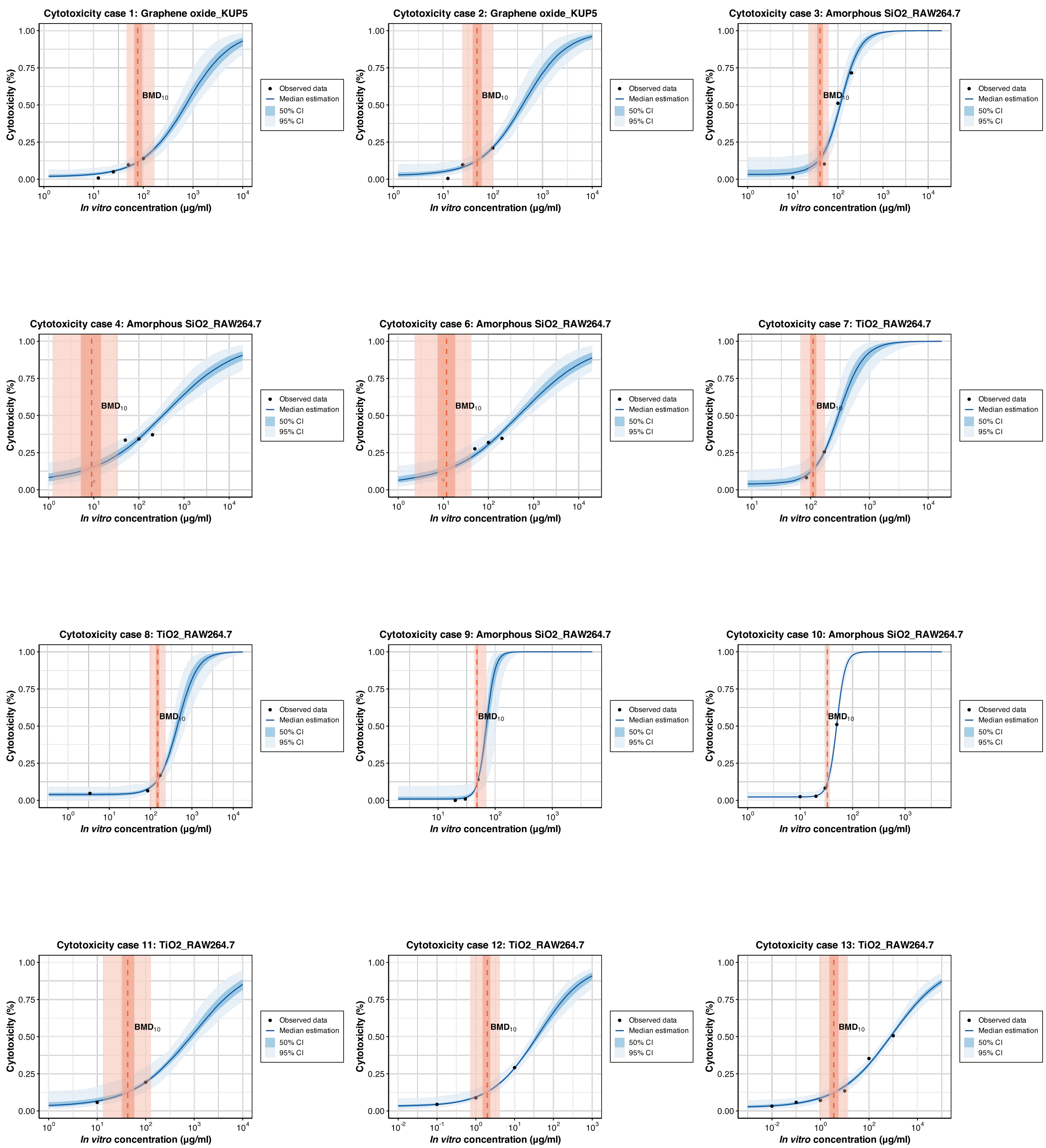
Cytotoxicity case 1: Graphene oxide_KUP5
Cytotoxicity case 2: Graphene oxide_KUP5
Cytotoxicity case 3: Amorphous SiO2_RAW264.7
Cytotoxicity case 4: Amorphous SiO2_RAW264.7
Cytotoxicity case 6: Amorphous SiO2_RAW264.7
Cytotoxicity case 7: TiO2_RAW264.7
Cytotoxicity case 8: TiO2_RAW264.7
Cytotoxicity case 9: Amorphous SiO2_RAW264.7
Cytotoxicity case 10: Amorphous SiO2_RAW264.7
Cytotoxicity case 11: TiO2_RAW264.7
Cytotoxicity case 12: TiO2_RAW264.7
Cytotoxicity case 13: TiO2_RAW264.7
Cytotoxicity (%)
In vitro concentration (μg/ml)
BMD10
Observed data
Median estimation
50% CI
95% CI

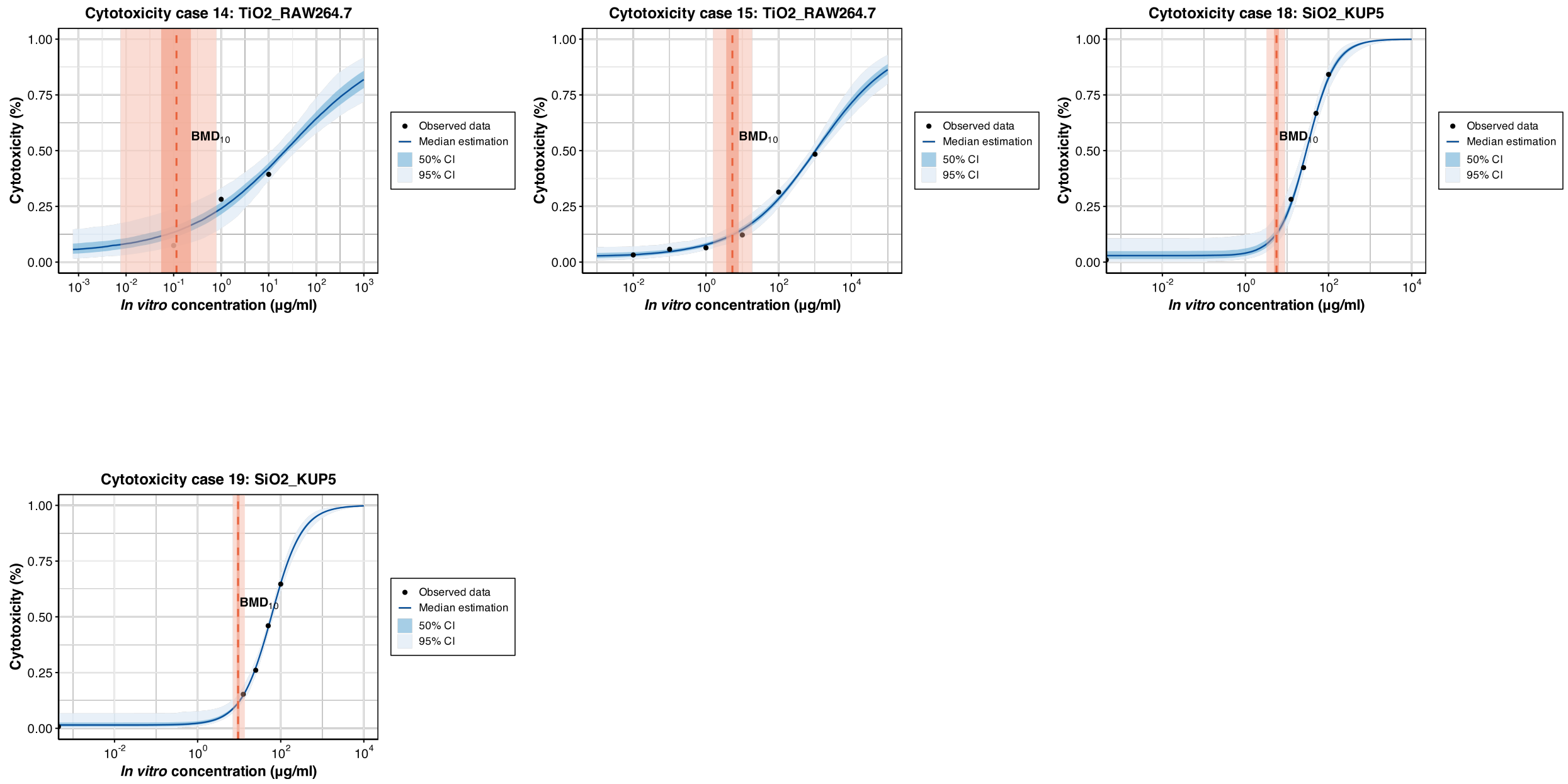

Figure S11. *Dose response curves for mouse cytotoxicity experiments retained for in vitro to in vivo extrapolation. Formatting follows Figure S1.*

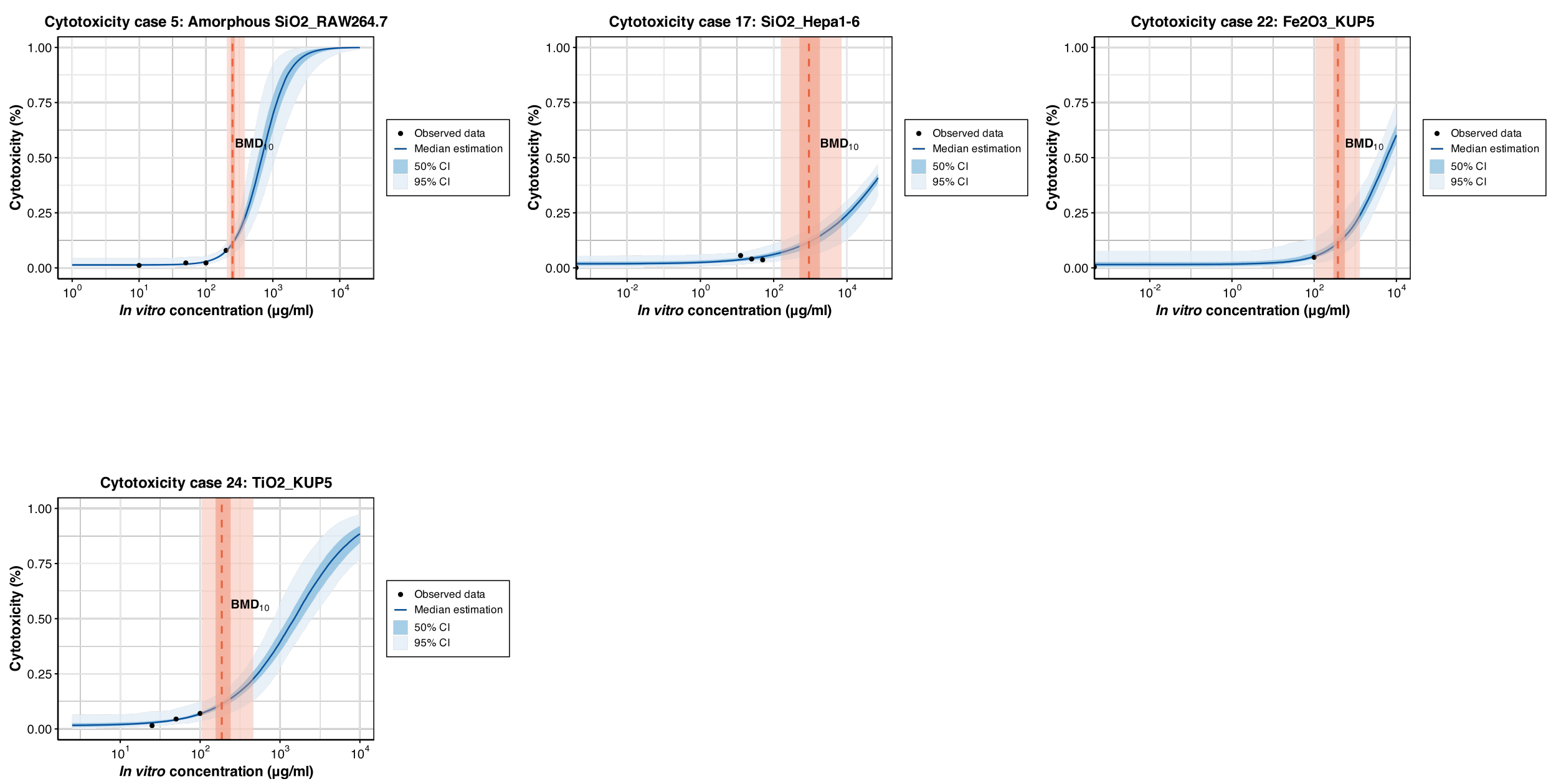

Figure S12. *Dose response curves for mouse cytotoxicity experiments excluded from further extrapolation due to insufficient response magnitude. These experiments showed less than 10% change from the negative control across all tested concentrations, precluding reliable BMD_{10} estimation. Formatting follows Figure S1.*

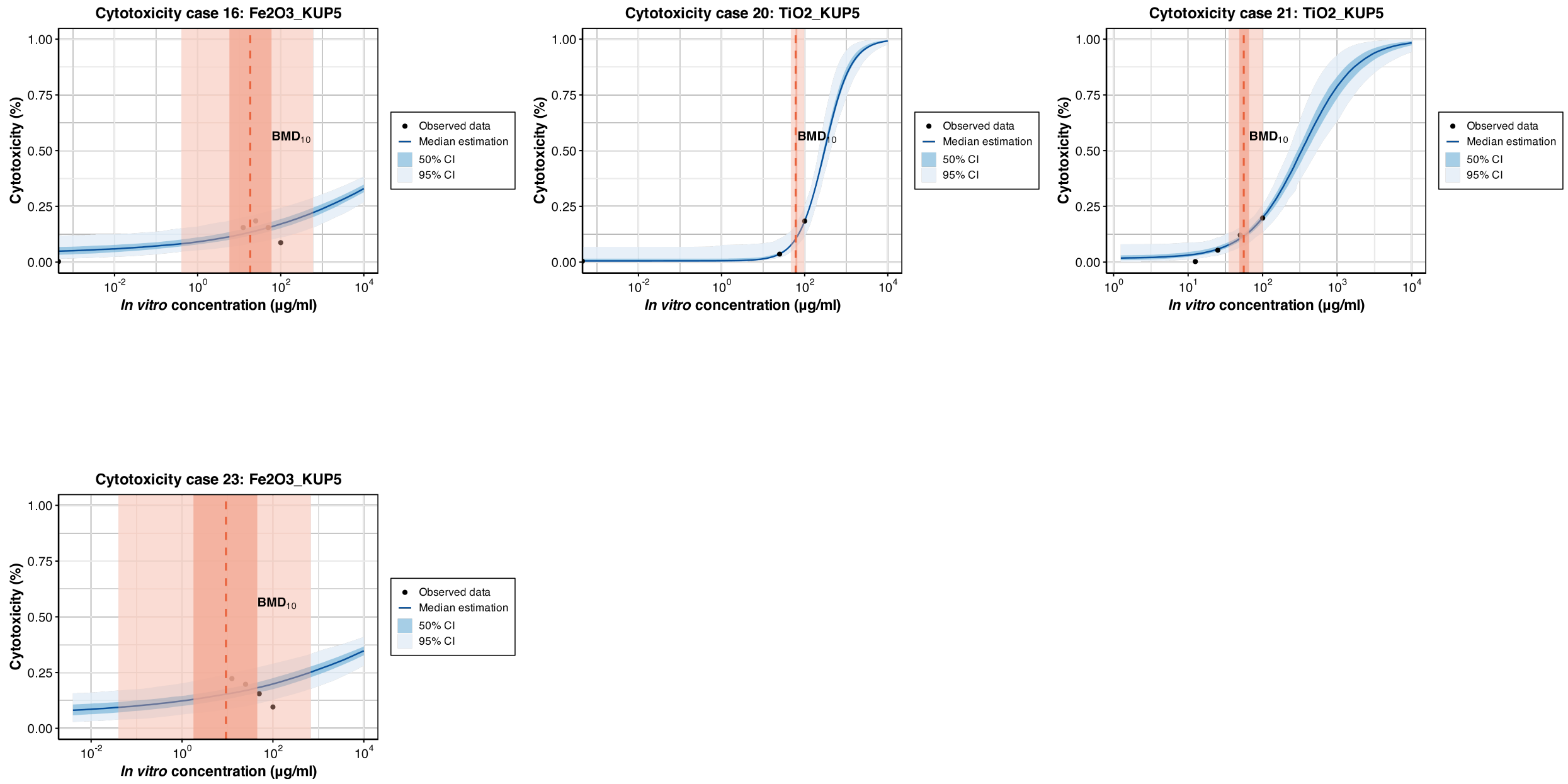

Figure S13. *Dose response curves for mouse cytotoxicity experiments excluded from further extrapolation due to excessive BMD_{10} uncertainty. These experiments yielded BMD_{10} credible intervals spanning more than three orders of magnitude, indicating insufficient constraint on the dose–response relationship. Formatting follows Figure S1.*

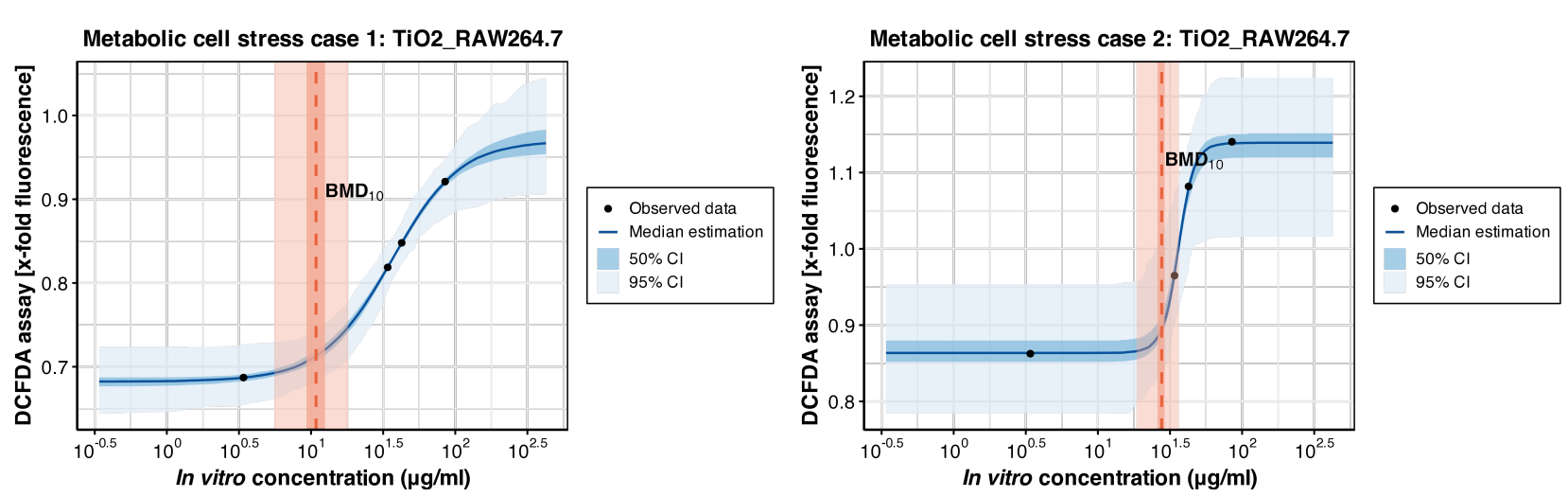

Figure S14. *Dose response curves for mouse metabolic cell stress experiments retained for in vitro to in vivo extrapolation. Formatting follows Figure S1.*

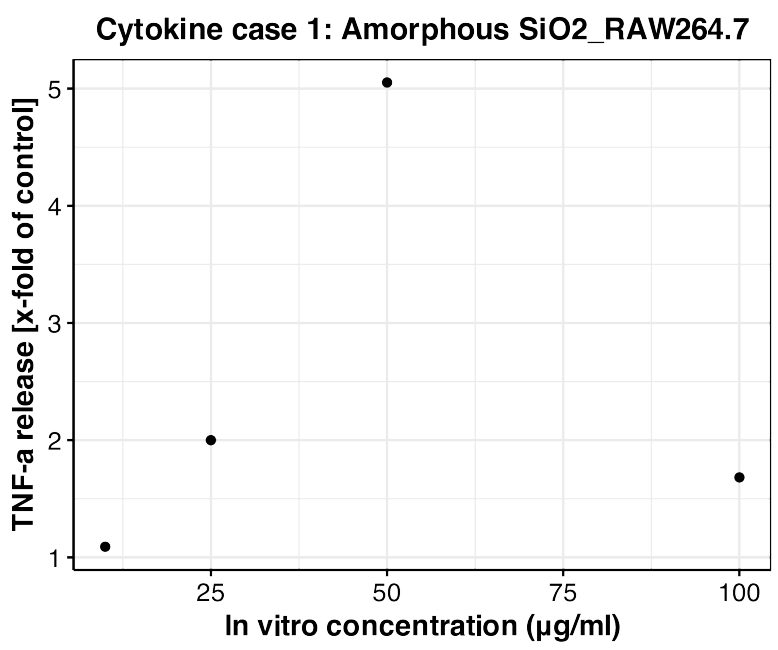
Cytokine case 1: Amorphous SiO2_RAW264.7
TNF-a release [x-fold of control]
1
2
3
4
5
25
50
75
100
In vitro concentration (µg/ml)

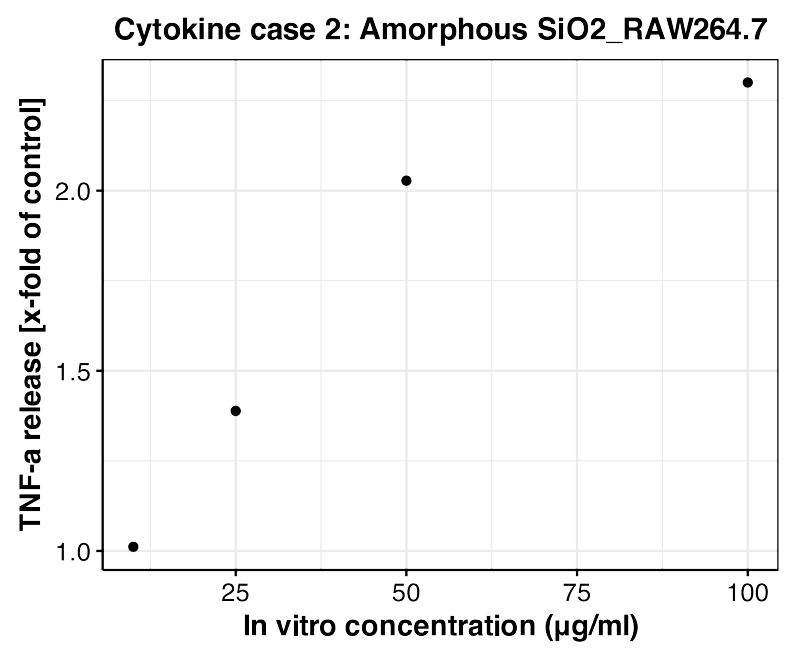
Cytokine case 2: Amorphous SiO2_RAW264.7
TNF-a release [x-fold of control]
1.0
1.5
2.0
25
50
75
100
In vitro concentration (µg/ml)

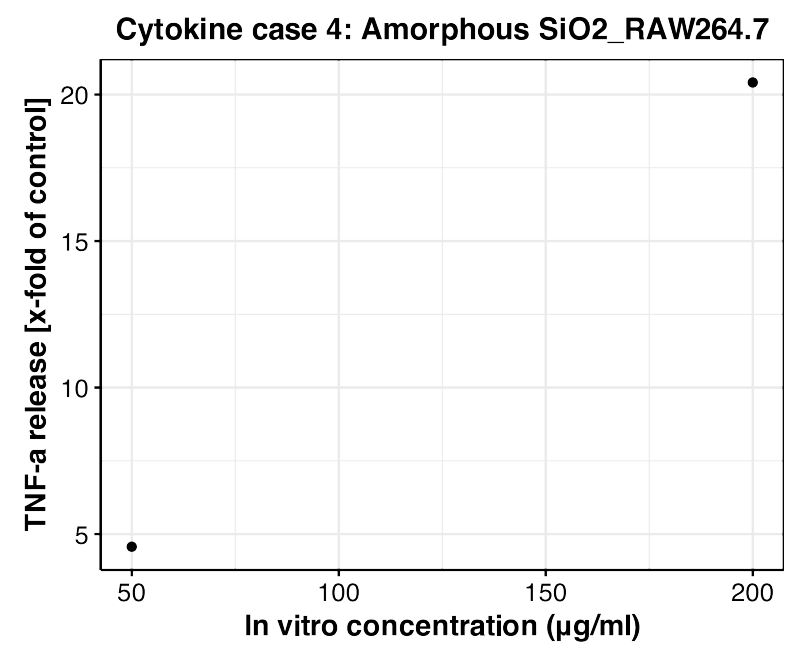
Cytokine case 4: Amorphous SiO2_RAW264.7
TNF-a release [x-fold of control]
5
10
15
20
50
100
150
200
In vitro concentration (µg/ml)

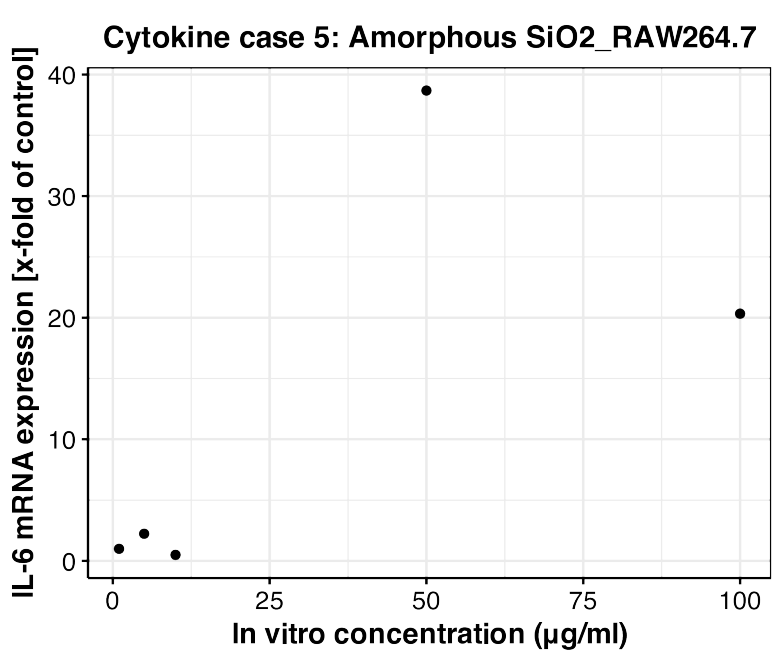
Cytokine case 5: Amorphous SiO2_RAW264.7
IL-6 mRNA expression [x-fold of control]
0
10
20
30
40
0
25
50
75
100
In vitro concentration (µg/ml)

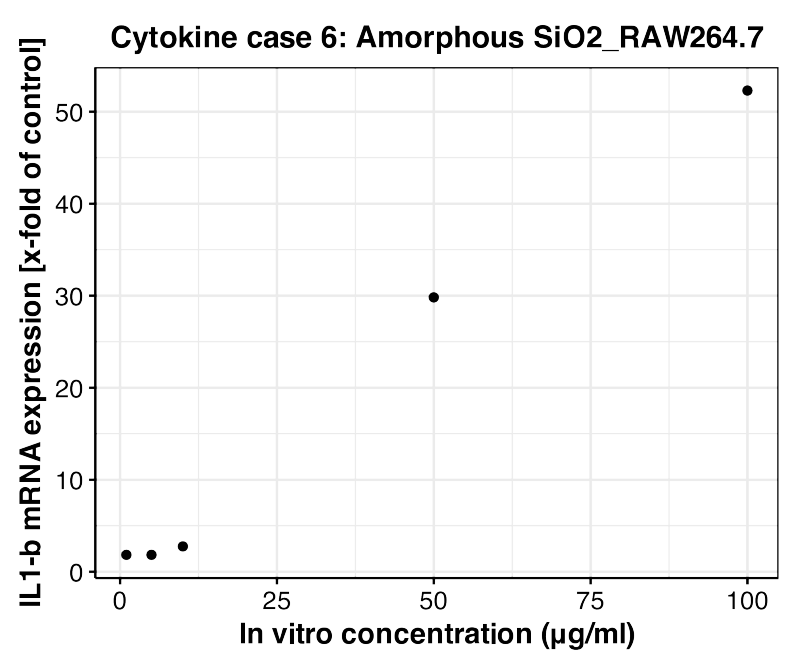
Cytokine case 6: Amorphous SiO2_RAW264.7
IL1-b mRNA expression [x-fold of control]
0
10
20
30
40
50
0
25
50
75
100
In vitro concentration (µg/ml)

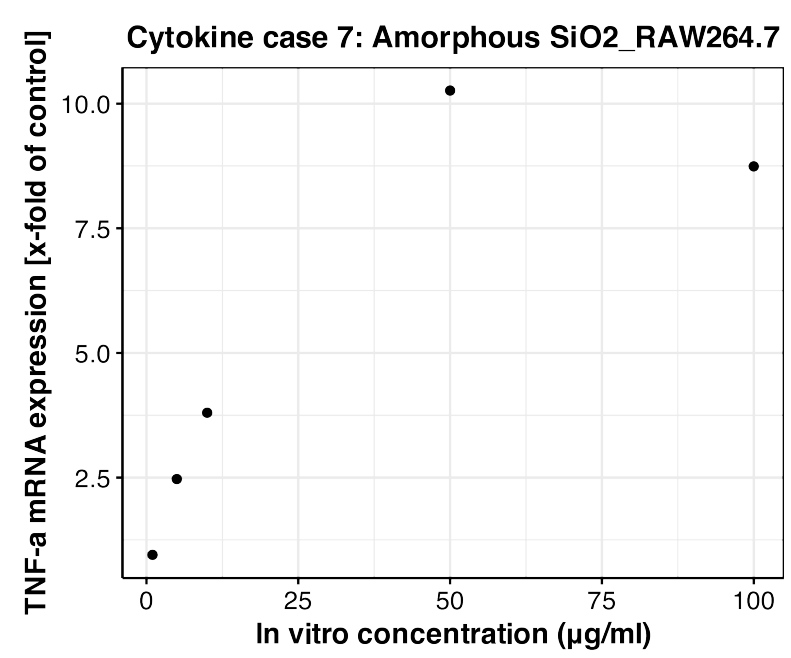
Cytokine case 7: Amorphous SiO2_RAW264.7
TNF-a mRNA expression [x-fold of control]
2.5
5.0
7.5
10.0
0
25
50
75
100
In vitro concentration (µg/ml)

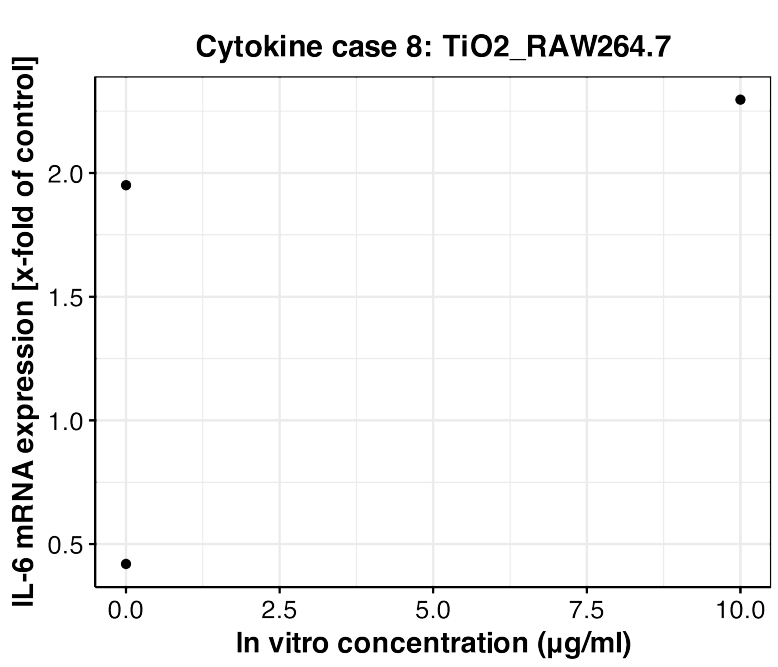
Cytokine case 8: TiO2_RAW264.7
IL-6 mRNA expression [x-fold of control]
0.5
1.0
1.5
2.0
0.0
2.5
5.0
7.5
10.0
In vitro concentration (µg/ml)

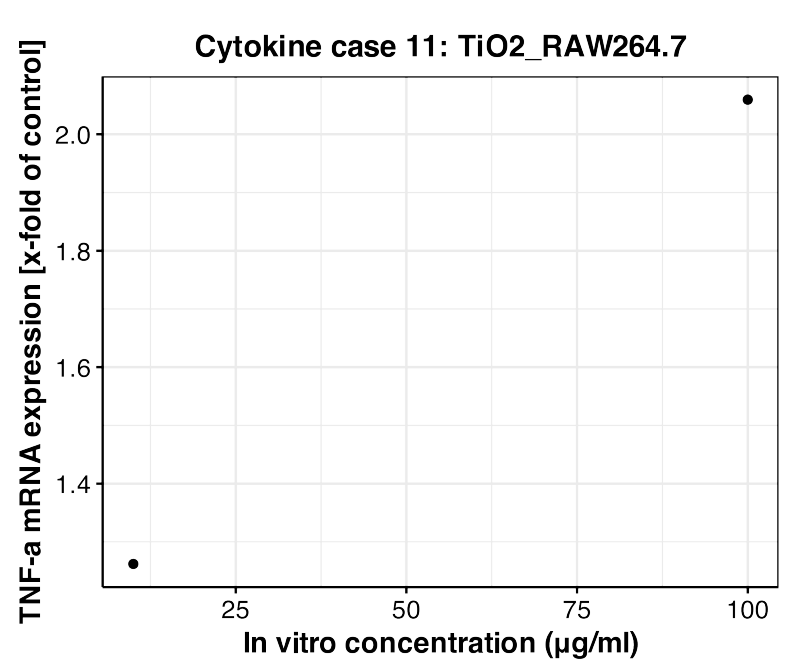
Cytokine case 11: TiO2_RAW264.7
TNF-a mRNA expression [x-fold of control]
1.4
1.6
1.8
2.0
25
50
75
100
In vitro concentration (µg/ml)

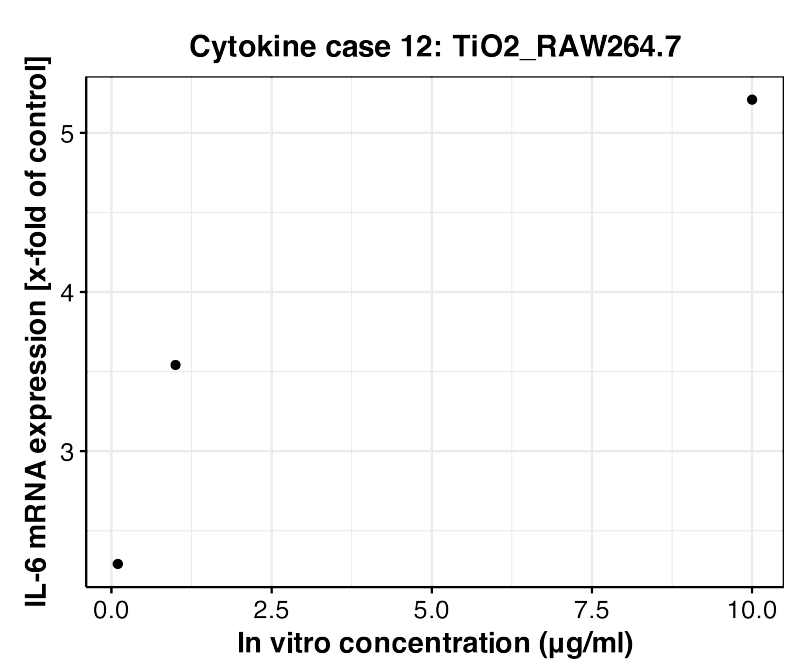
Cytokine case 12: TiO2_RAW264.7
IL-6 mRNA expression [x-fold of control]
3
4
5
0.0
2.5
5.0
7.5
10.0
In vitro concentration (µg/ml)

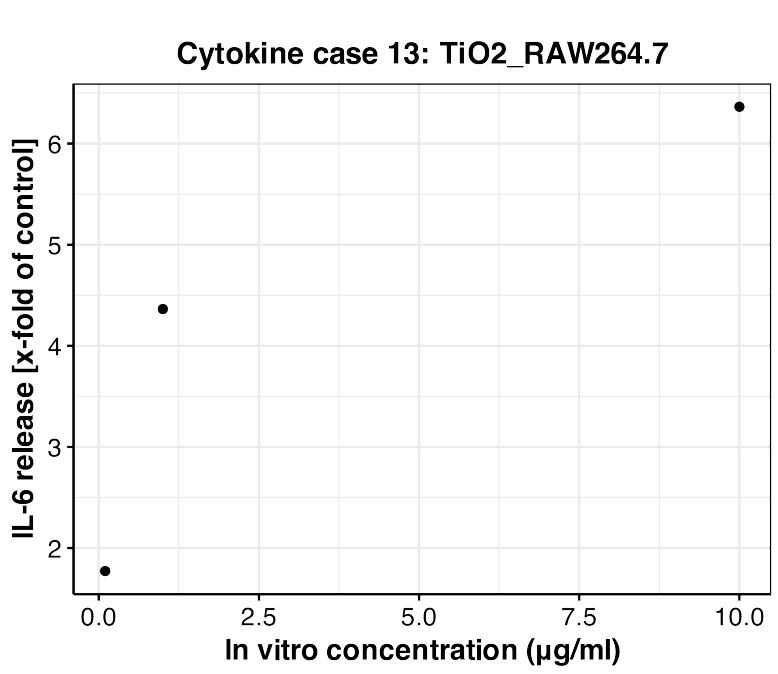
Cytokine case 13: TiO2_RAW264.7
IL-6 release [x-fold of control]
2
3
4
5
6
0.0
2.5
5.0
7.5
10.0
In vitro concentration (µg/ml)

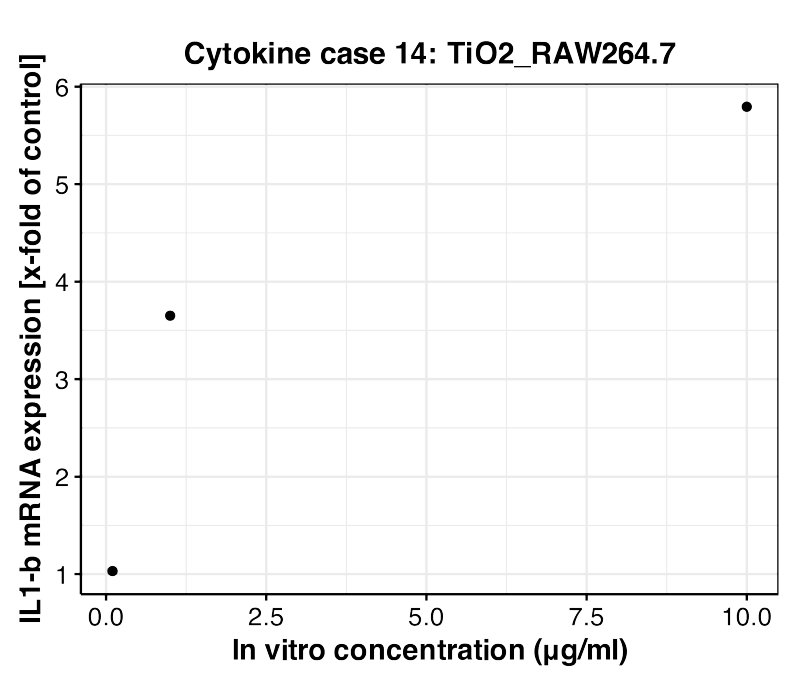
Cytokine case 14: TiO2_RAW264.7
IL1-b mRNA expression [x-fold of control]
1
2
3
4
5
6
0.0
2.5
5.0
7.5
10.0
In vitro concentration (µg/ml)

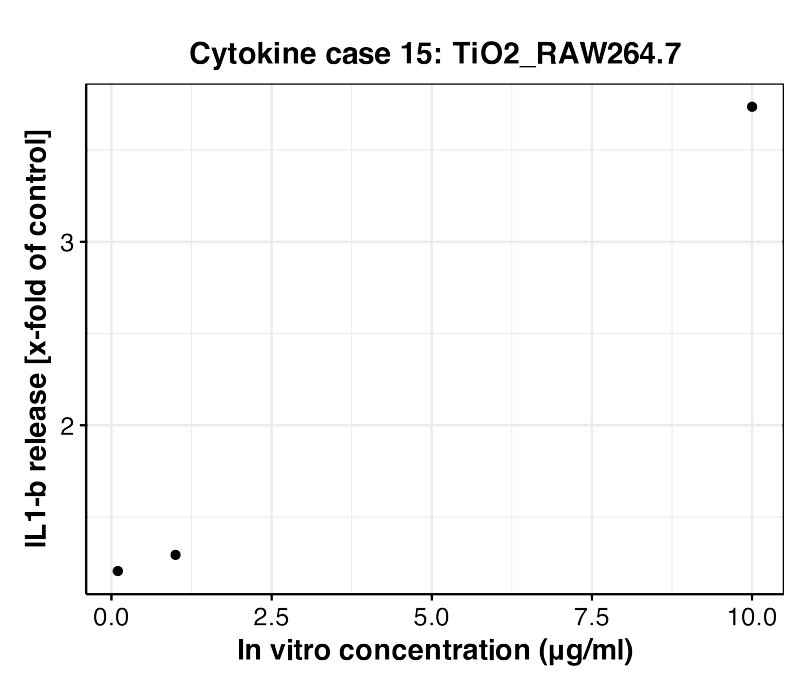
Cytokine case 15: TiO2_RAW264.7
IL1-b release [x-fold of control]
2
3
0.0
2.5
5.0
7.5
10.0
In vitro concentration (µg/ml)

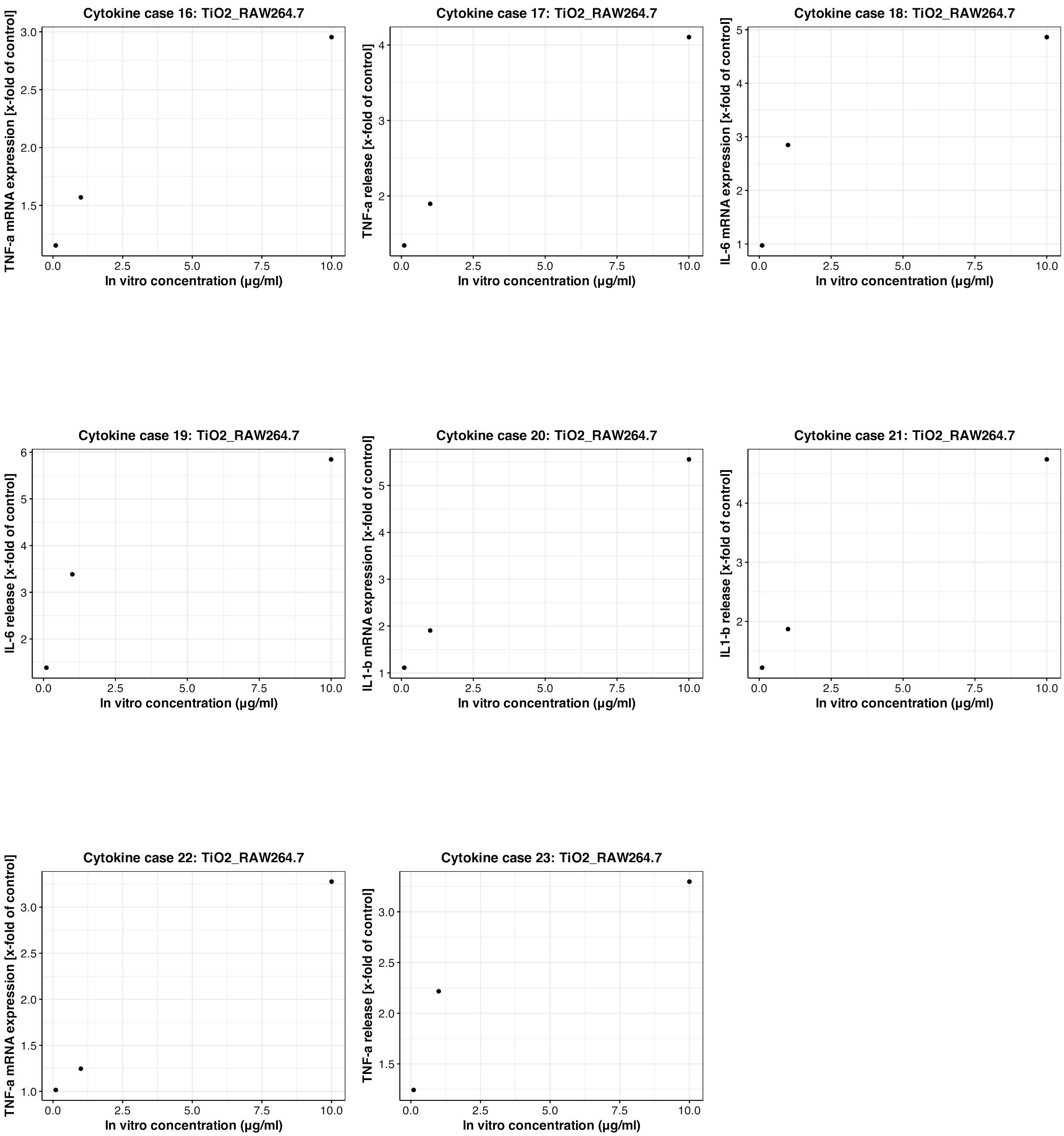

Figure S15. *Cytokine endpoint data for mouse in vitro experiments. Each panel shows measured cytokine responses (e.g., TNF-α, IL-6, IL-8, IL-1β) as a function of nanoparticle concentration. Classical dose response curve fitting was not applied to these endpoints due to their non-monotonic or threshold-triggered release profiles.*

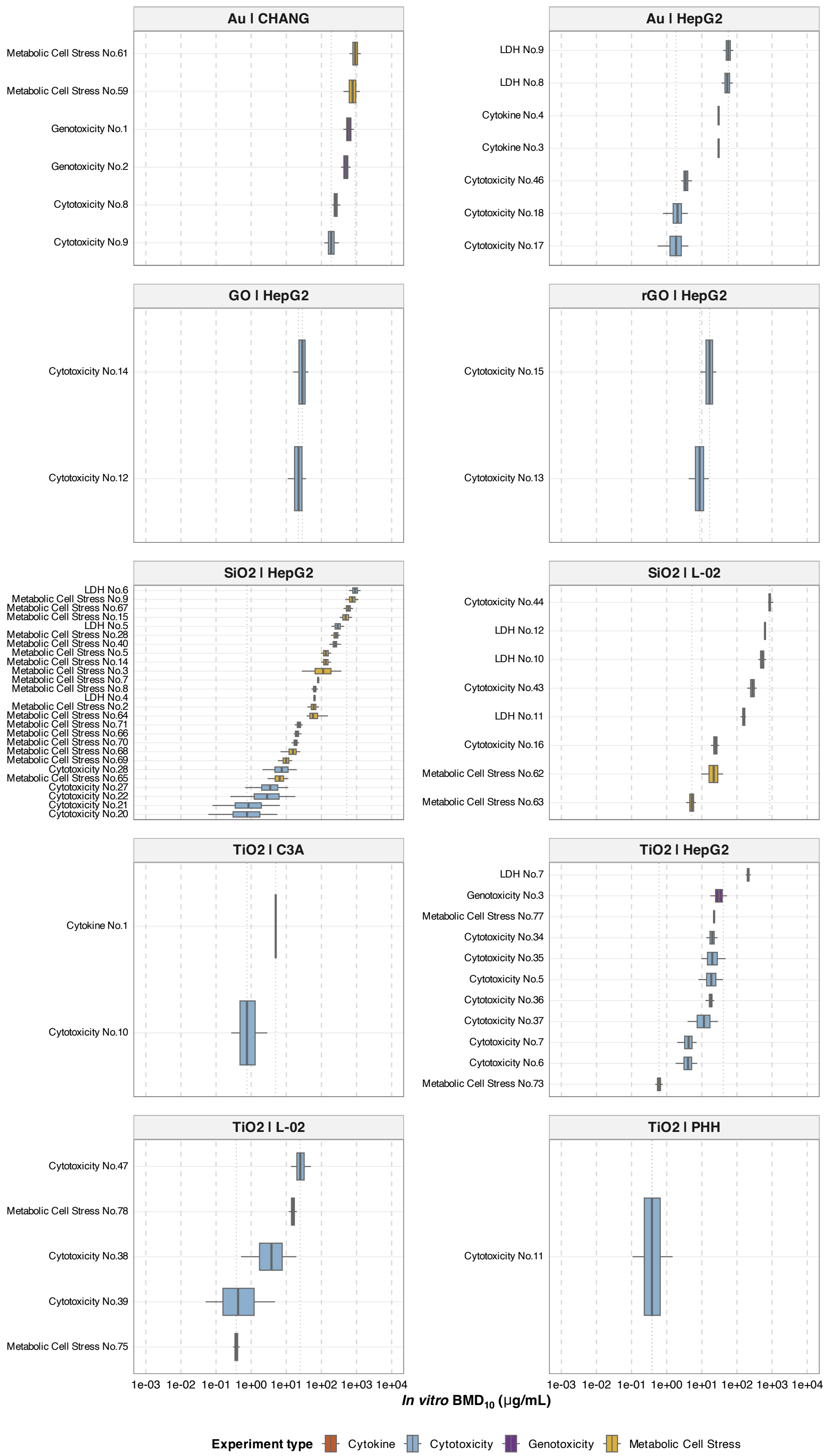
Au | CHANG
Metabolic Cell Stress No.61
Metabolic Cell Stress No.59
Genotoxicity No.1
Genotoxicity No.2
Cytotoxicity No.8
Cytotoxicity No.9
Au | HepG2
LDH No.9
LDH No.8
Cytokine No.4
Cytokine No.3
Cytotoxicity No.46
Cytotoxicity No.18
Cytotoxicity No.17
GO | HepG2
Cytotoxicity No.14
Cytotoxicity No.12
rGO | HepG2
Cytotoxicity No.15
Cytotoxicity No.13
SiO2 | HepG2
SiO2 | L-02
TiO2 | C3A
Cytokine No.1
Cytotoxicity No.10
TiO2 | HepG2
TiO2 | L-02
TiO2 | PHH
Cytotoxicity No.11
1e-03 1e-02 1e-01 1e+00 1e+01 1e+02 1e+03 1e+04
In vitro BMD10 (µg/mL)
Experiment type
Cytokine
Cytotoxicity
Genotoxicity
Metabolic Cell Stress

Figure S16. *Experiment-level BMD_{10} credible intervals (µg/mL) for human in vitro liver toxicity datasets, faceted by nanoparticle core and cell type. Each horizontal interval summarises the posterior BMD_{10} distribution for one experiment: the box spans the 50% credible interval (25th–75th percentiles), the vertical line marks the posterior median, and whiskers span the 90% credible interval (5th–95th percentiles). Box fill color indicates the assay category. Row labels indicate the assay category and study identifier.*

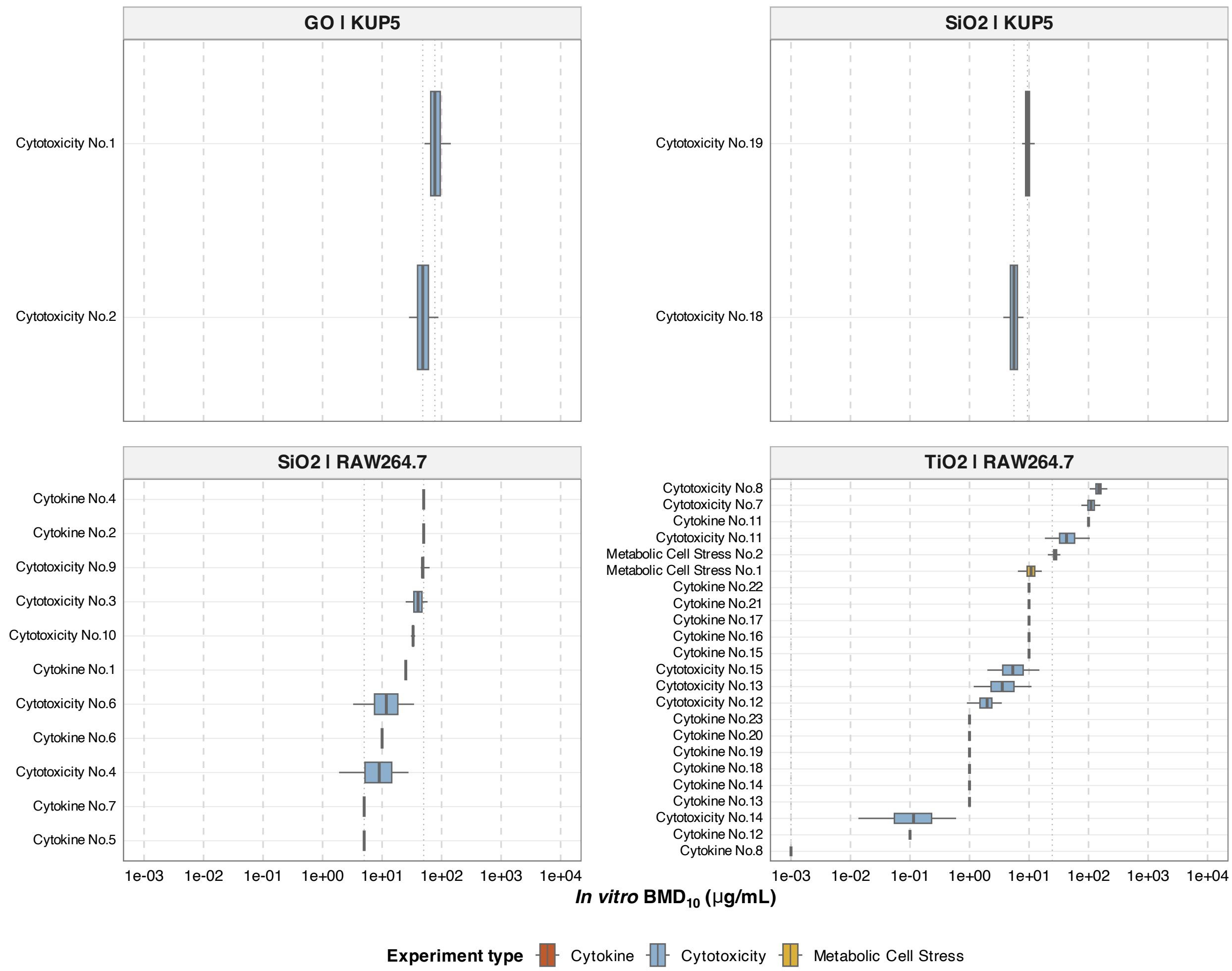

Figure S17. *Experiment-level BMD_{10} credible intervals (µg/mL) for mouse in vitro liver toxicity datasets, faceted by nanoparticle core and cell type. Each horizontal interval summarises the posterior BMD_{10} distribution for one experiment: the box spans the 50% credible interval (25th–75th percentiles), the vertical line marks the posterior median, and whiskers span the 90% credible interval (5th–95th percentiles). Box fill color indicates the assay category. Row labels indicate the assay category and study identifier.*

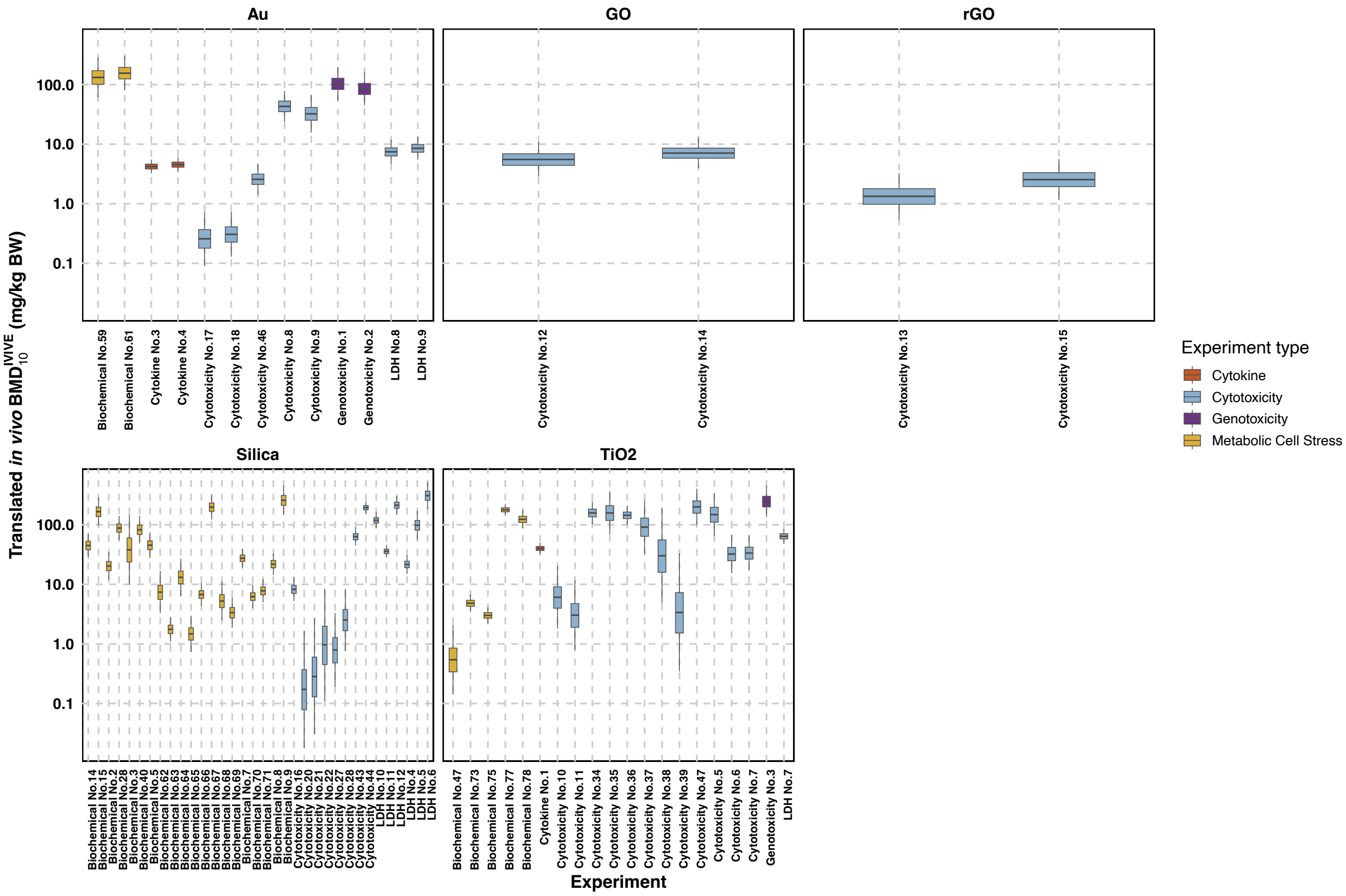

Figure S18. *Experiment-level human* BMD_{10}^{IVIVE} *credible intervals (mg/kg body weight) derived from in vitro liver toxicity datasets, faceted by nanoparticle core and cell type. Each horizontal box-whisker plot represents a single experiment: the box spans the 25th–75th percentile, the vertical line marks the median, and whiskers extend to the 5th and 95th percentiles of the PBPK-translated safety limit distribution. Box fill color indicates the assay category. Row labels indicate the assay category and study identifier.*

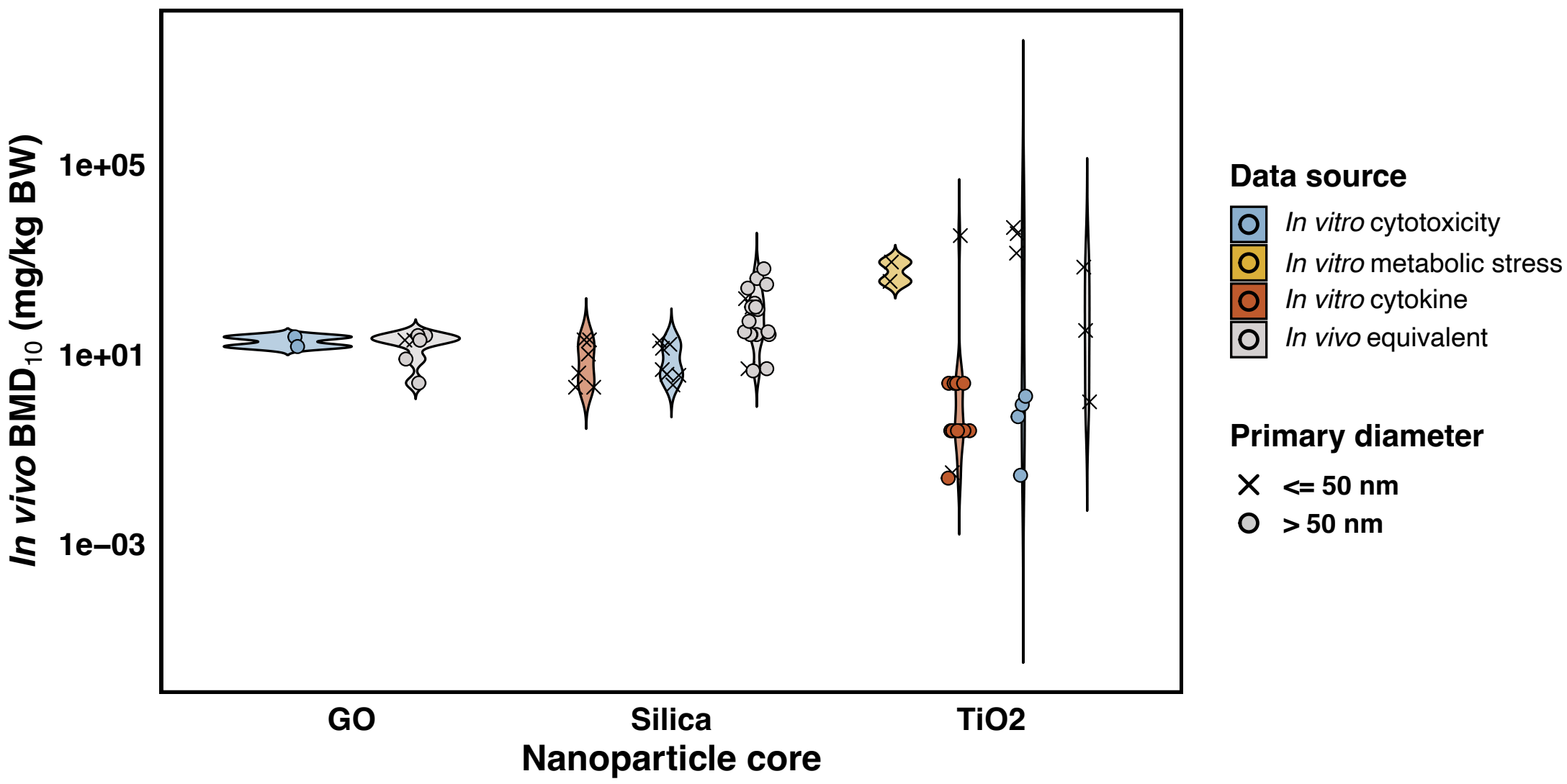

Figure S19. *Distribution of in vivo BMD*$_{10}$ *distribution from different data sources by nanoparticle type stratified by primary diameter. Small symbols indicate individual experiments. Gray color represents translated* BMD_{10}^{eq} *from the literature in vivo studies, while the other colored points represent in vivo* BMD_{10}^{IVIVE} *translated from in vitro BMD*$_{10}$ *using the PBPK model.*

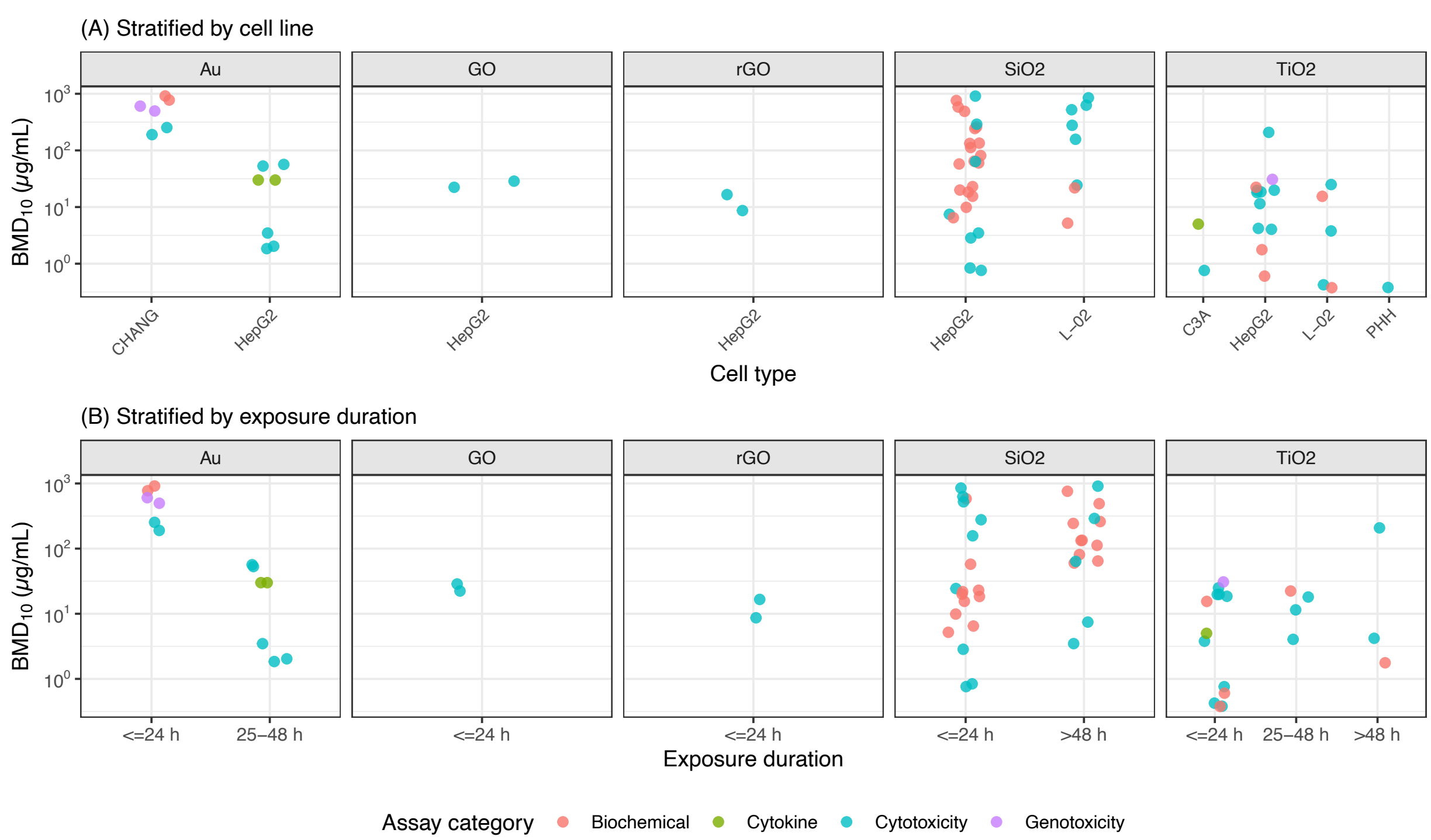

Figure S20. *Subgroup analysis of human in vitro BMD*$_{10}$*.*

References

[1] Kim E, Kim S-H, Kim H-C, Lee SG, Lee SJ, Jeong SW, 2011. Growth inhibition of aquatic plant caused by silver and titanium oxide nanoparticles. Toxicol Environ Health Sci 3, 1–6. https://doi.org/10.1007/s13530-011-0071-8

[2] Schwab F, Bucheli TD, Lukhele LP, Magrez A, Nowack B, Sigg L, Knauer K, 2011. Are Carbon Nanotube Effects on Green Algae Caused by Shading and Agglomeration? Environ Sci Technol 45, 6136–6144. https://doi.org/10.1021/es200506b

[3] European Chemicals Agency, 2008. Guidance on information requirements and chemical safety assessment :guidance for the implementation of REACH. Volume 8, Dose (concentration), response characterisation. Publications Office of the European Union

[4] Beasley A, Belanger SE, Brill JL, Otter RR, 2015. Evaluation and comparison of the relationship between NOEC and EC10 or EC20 values in chronic Daphnia toxicity testing. Environ Toxicol Chem 34, 2378–2384. https://doi.org/10.1002/etc.3086

[5] Al A et, 2015. Comparison of NOEC values to EC10/EC20 values, including confidence intervals, in aquatic and terrestrial ecotoxicological risk assessment. EFSA Support Publ 12, 906E. https://doi.org/10.2903/sp.efsa.2015.EN-906

[6] Wen K-P, Chen Y-C, Chuang C-H, Chang H-Y, Lee C-Y, Tai N-H, 2015. Accumulation and toxicity of intravenously-injected functionalized graphene oxide in mice. J Appl Toxicol 35, 1211–1218. https://doi.org/10.1002/jat.3187

[7] Li B, Zhang X-Y, Yang J-Z, Zhang Y-J, Li W-X, Fan C-H, Huang Q, 2014. Influence of polyethylene glycol coating on biodistribution and toxicity of nanoscale graphene oxide in mice after intravenous injection. Int J Nanomedicine 9, 4697–4707. https://doi.org/10.2147/IJN.S66591

[8] Xie G, Sun J, Zhong G, Shi L, Zhang D, 2010. Biodistribution and toxicity of intravenously administered silica nanoparticles in mice. Arch Toxicol 84, 183–190. https://doi.org/10.1007/s00204-009-0488-x

[9] Fu C, Liu T, Tang F, Chen D, Li L, Liu H, Li X, 2012. Acute toxicity and oxidative damage induced by silica nanorattle in vivo. Chin Sci Bull 57, 2525–2532. https://doi.org/10.1007/s11434-012-5187-y

[10] Fu C, Liu T, Li L, Liu H, Chen D, Tang F, 2013. The absorption, distribution, excretion and toxicity of mesoporous silica nanoparticles in mice following different exposure routes. Biomaterials 34, 2565–2575. https://doi.org/10.1016/j.biomaterials.2012.12.043

[11] Yu Y, Li Y, Wang W, Jin M, Du Z, Li Y, Duan J, Yu Y, Sun Z, 2013. Acute Toxicity of Amorphous Silica Nanoparticles in Intravenously Exposed ICR Mice. PLOS ONE 8, e61346. https://doi.org/10.1371/journal.pone.0061346

[12] Greish K, Thiagarajan G, Herd H, Price R, Bauer H, Hubbard D, Burckle A, Sadekar S, Yu T, Anwar A, Ray A, Ghandehari H, 2012. Size and surface charge significantly influence the toxicity of silica and dendritic nanoparticles. Nanotoxicology 6, 713–723. https://doi.org/10.3109/17435390.2011.604442

[13] Xu J, Shi H, Ruth M, Yu H, Lazar L, Zou B, Yang C, Wu A, Zhao J, 2013. Acute Toxicity of Intravenously Administered Titanium Dioxide Nanoparticles in Mice. PLOS ONE 8, e70618. https://doi.org/10.1371/journal.pone.0070618

[14] Yang K, Wan J, Zhang S, Zhang Y, Lee S-T, Liu Z, 2011. In Vivo Pharmacokinetics, Long-Term Biodistribution, and Toxicology of PEGylated Graphene in Mice. ACS Nano 5, 516–522. https://doi.org/10.1021/nn1024303

[15] Nabeshi H, Yoshikawa T, Matsuyama K, Nakazato Y, Arimori A, Isobe M, Tochigi S, Kondoh S, Hirai T, Akase T, Yamashita T, Yamashita K, Yoshida T, Nagano K, Abe Y, Yoshioka Y, Kamada H, Imazawa T, Itoh N, Kondoh M, Yagi K, Mayumi T, Tsunoda S, Tsutsumi Y, 2012. Amorphous nanosilicas induce consumptive coagulopathy after systemic exposure. Nanotechnology 23, 045101. https://doi.org/10.1088/0957-4484/23/4/045101

[16] Liu T, Li L, Teng X, Huang X, Liu H, Chen D, Ren J, He J, Tang F, 2011. Single and repeated dose toxicity of mesoporous hollow silica nanoparticles in intravenously exposed mice. Biomaterials 32, 1657–1668. https://doi.org/10.1016/j.biomaterials.2010.10.035

[17] Liang S, Xu S, Zhang D, He J, Chu M, 2015. Reproductive toxicity of nanoscale graphene oxide in male mice. Nanotoxicology 9, 92–105. https://doi.org/10.3109/17435390.2014.893380

[18] Chan W-T, Liu C-C, Chiau J-SC, Tsai S-T, Liang C-K, Cheng M-L, Lee H-C, Yeung C-Y, Hou S-Y, 2017. In vivo toxicologic study of larger silica nanoparticles in mice. Int J Nanomedicine 12, 3421–3432. https://doi.org/10.2147/IJN.S126823

[19] Yang K, Wan J, Zhang S, Tian B, Zhang Y, Liu Z, 2012. The influence of surface chemistry and size of nanoscale graphene oxide on photothermal therapy of cancer using ultra-low laser power. Biomaterials 33, 2206–2214. https://doi.org/10.1016/j.biomaterials.2011.11.064

[20] Qu G, Wang X, Liu Q, Liu R, Yin N, Ma J, Chen L, He J, Liu S, Jiang G, 2013. The *ex vivo* and *in vivo* biological performances of graphene oxide and the impact of surfactant on graphene oxide's biocompatibility. J Environ Sci 25, 873–881. https://doi.org/10.1016/S1001-0742(12)60252-6

[21] Yamashita K, Yoshioka Y, Higashisaka K, Mimura K, Morishita Y, Nozaki M, Yoshida T, Ogura T, Nabeshi H, Nagano K, Abe Y, Kamada H, Monobe Y, Imazawa T, Aoshima H, Shishido K, Kawai Y, Mayumi T, Tsunoda S, Itoh N, Yoshikawa T, Yanagihara I, Saito S, Tsutsumi Y, 2011. Silica and titanium dioxide nanoparticles cause pregnancy complications in mice. Nat Nanotechnol 6, 321–328. https://doi.org/10.1038/nnano.2011.41

[22] Bihari P, Holzer M, Praetner M, Fent J, Lerchenberger M, Reichel CA, Rehberg M, Lakatos S, Krombach F, 2010. Single-walled carbon nanotubes activate platelets and accelerate thrombus formation in the microcirculation. Toxicology 269, 148–154. https://doi.org/10.1016/j.tox.2009.08.011
